# Reactivity of a Strictly T‐Shaped Phosphine Ligated by an Acridane Derived NNN Pincer Ligand

**DOI:** 10.1002/chem.202300818

**Published:** 2023-05-23

**Authors:** Aaron J. King, Josh Abbenseth, Jose M. Goicoechea

**Affiliations:** ^1^ Department of Chemistry University of Oxford Chemistry Research Laboratory 12 Mansfield Road Oxford OX1 3TA UK; ^2^ Institut für Chemie Humboldt-Universität zu Berlin Brook-Taylor-Straße 2 12489 Berlin Germany; ^3^ Department of Chemistry Indiana University 800 E. Kirkland Ave. Bloomington, In 47401 USA

**Keywords:** cooperativity, phosphorus, pincer ligands, small molecule activation, water activation

## Abstract

The steric tuning of a tridentate acridane‐derived NNN pincer ligand allows for the isolation of a strictly T‐shaped phosphine that exhibits ambiphilic reactivity. Well‐defined phosphorus‐centered reactivity towards nucleophiles and electrophiles is reported, contrasting with prior reports on this class of compounds. Reactions towards oxidants are also described. The latter result in the two‐electron oxidation of the phosphorus atom from +III to +V and are accompanied by a strong geometric distortion of the NNN pincer ligand. By contrast, cooperative activation of E−H (HCl, HBcat, HOMe) bonds proceeds with retention of the phosphorus redox state. When using H_2_O as a substrate, the reaction results in the full disassembly of H_2_O to its constituent atoms, highlighting the potential of this platform for small molecule activation reactions.

## Graphical abstract

The synthesis and reactivity of a strictly T‐shaped phosphine derived from an NNN acridane pincer ligand is reported. Phosphorus‐centered reactivity towards nucleophiles and electrophiles is observed, and the activation of hydridic and protic E−H bonds is demonstrated, including complete disassembly of water.

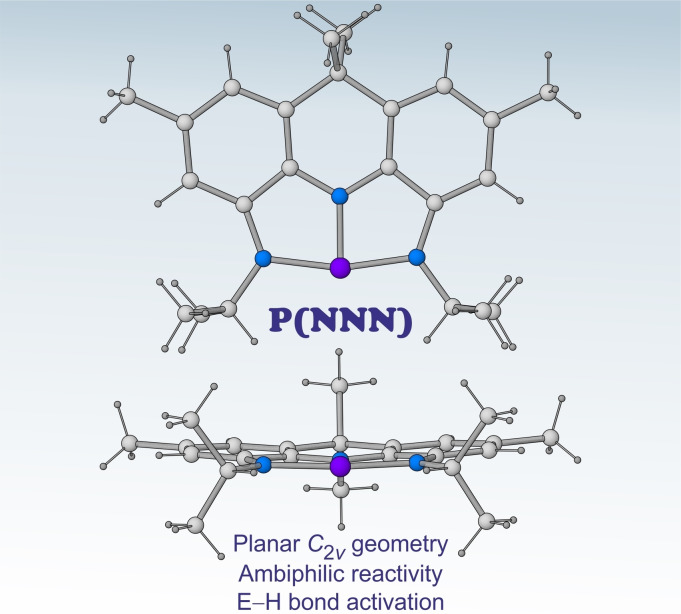

## Introduction

**Figure 1:**
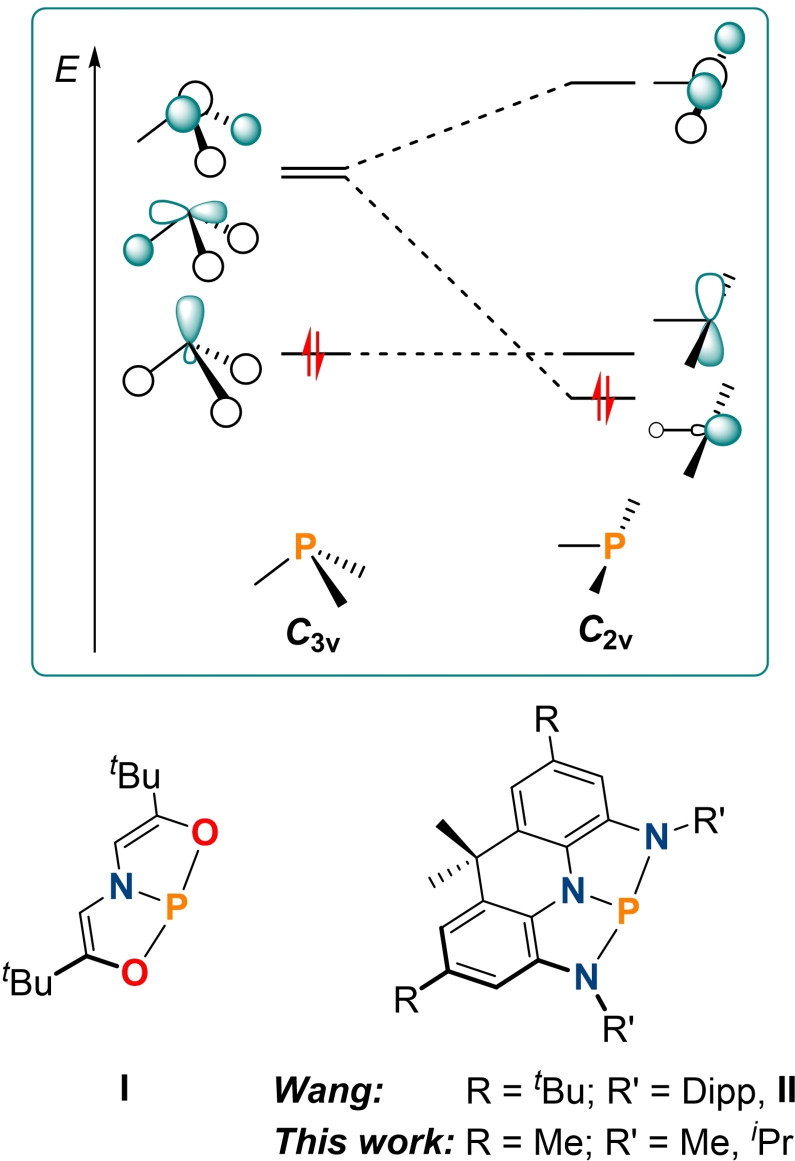
The effect of changing the geometry of a phosphine from *C*_3*v*_ towards *C*_2*v*_ upon geometric constraint (top); *C*_2*v*_ symmetric geometrically constrained phosphines reported in the literature (bottom). Dipp=2,6‐diisopropylphenyl.

The ability of certain main group elements to mimic reactivity inherently associated with transition metals has recently become a concept of significant interest.[^1^, ^2^, ^3^, ^4^, ^5^] Amongst the elements of the p‐block, phosphorus has emerged as a highly viable candidate for such a purpose due to its accessible phosphorus(III/V) redox couple, which facilitates the required electron transfer processes for oxidative‐addition and reductive‐elimination reactions.[^6^, ^7^, ^8^, ^9^] Judicious choice of a co‐ligand can be used to facilitate cooperative substrate activation as well.^[10]^ While phosphines predominantly exhibit nucleophilic reactivity, the incorporation of phosphorus into tridentate pincer ligands enforces a non‐VSEPR symmetry, and permits phosphorus‐based ambiphilic behavior. This is a consequence of lifting the degeneracy of the σ‐antibonding LUMOs (lowest unoccupied molecular orbitals) found in trigonal phosphines, resulting in a reduced HOMO‐LUMO separation that enables electron donor *and* acceptor properties at phosphorus. Full perturbation towards a T‐shape (or *C*_2*v*_ symmetry) results in a frontier‐orbital arrangement that is highly reminiscent of those found in tetrylenes, albeit with a significantly reduced directionality of the s‐type lone pair situated at the phosphorus center (Figure 1, top).[^11^, ^12^, ^13^, ^14^] Several geometrically constrained phosphines (and phosphenium cations, i. e. PR_2_^+^ species) with ONO,[^15^, ^16^, ^17^, ^18^, ^19^, ^20^, ^21^, ^22^, ^23^, ^24^, ^25^, ^26^, ^27^, ^28^, ^29^] OON,^[30]^ CCC,[^31^, ^32^, ^33^] NNN,[^34^, ^35^, ^36^, ^37^, ^38^, ^39^, ^40^, ^41^, ^42^, ^43^, ^44^, ^45^, ^46^, ^47^, ^48^, ^49^] NPN,[^50^, ^51^] SPS,[^52^, ^53^, ^54^] or NNS^[55]^ donor sets have been reported, each showing unusual reactivity towards small molecules and unique spectroscopic and electronic properties.^[37]^

Due to the lack of rigidity of many of these reported pincer ligands, these species adopt a *bent* geometry. However, on account of the reduced HOMO‐LUMO gap, electrophilic substrate activation is most efficient at the *planar* T‐shaped conformer. Consequently, kinetic barriers for bond activation reactions are influenced by the required geometric distortion to access this more reactive state, impairing the activity of systems that cannot fully distort to a *C*_2*v*_ geometry. The strictly T‐shaped phosphine **I** (Figure 1, bottom), initially reported by the Arduengo group, and further investigated by Driess, Radosevich and co‐workers, has been shown to react with a plethora of small molecules.[^18^, ^25^, ^36^] To date, this is still the only strictly T‐shaped phosphine for which reactivity studies have been reported. The bis‐enolate ligand in **I**, however, proved to be highly reactive, especially towards protic E−H bonds, and was shown to participate in substrate activation reactions resulting in ligand derivatization or complete disassembly of the ligand scaffold.[^24^, ^25^, ^29^, ^36^] The redox chemistry of the acridane derived T‐shaped phosphine **II** was recently investigated by Wang and co‐workers (Figure 1, bottom).^[46]^ Although further chemistry was not reported, this system holds immense potential for phosphorus‐based reactivity and possible redox activity due to the increased chemical inertness and high rigidity of the ligand backbone.[^56^, ^57^, ^58^] In addition, extensive computational studies by Zhu, Zeng, Maeda, Sakaki and co‐workers have suggested that phosphines ligated by NNN donor sets, in particular acridane derived systems, could be highly active in small molecule activation reactions, for example catalytic CO_2_ reduction to formid acid,^[59]^ ammonia‐borane dehydrogenation,^[60]^ and even dinitrogen splitting.^[61]^

**Scheme 1:**
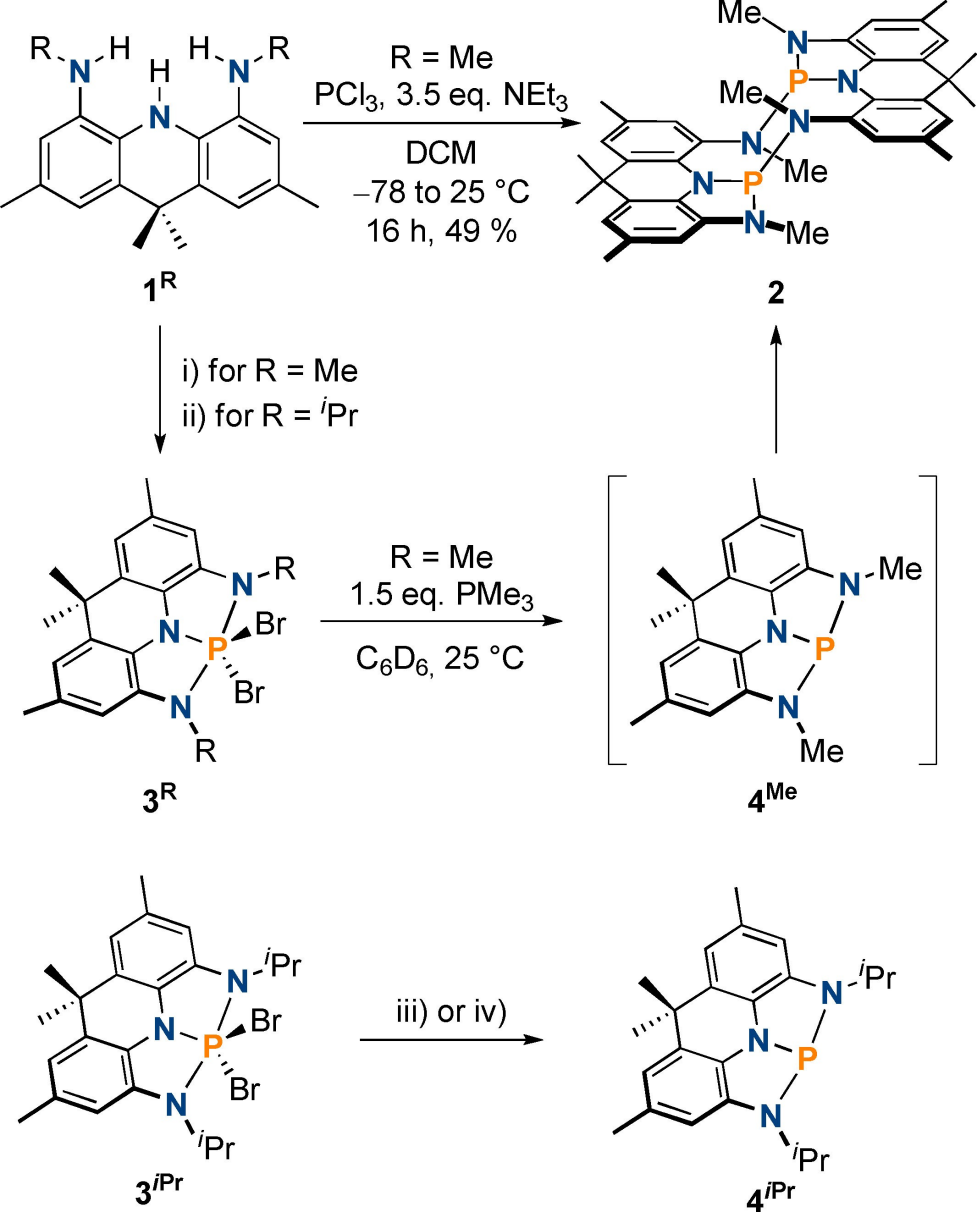
Reactivity of the protio‐ligand **1^R^** towards PCl_3_ and PBr_5_ in the presence of NEt_3_ to generate **2** and **3^R^** as well as conversion of **3^Me^** towards **2** via transient **4^Me^** (top); Generation of **4** ^***i*** **Pr**^ from **3** ^***i*** **Pr**^ upon dehalogenation with PMe_3_ or Mg (bottom); i) PBr_5_, 3 equiv. NEt_3_, toluene, −78 to 25 °C, 16 h, 64 % isolated yield; ii) PBr_5_, 5 equiv. NEt_3_, toluene, −78 to 25 °C, 16 h, 54 % isolated yield; iii) 5 equiv. PMe_3_, C_6_H_6_, 25 °C, 15 min, 90 % isolated yield; iv) 20 equiv. Mg, THF, 25 °C, 30 min, 90 % isolated yield.

We recently reported the synthesis of NNN pincer ligand **1** ^***i*** **Pr**^ (Scheme 1) and its use in the synthesis of tantalum(V) complexes for the activation of small molecule substrates.^[62]^ The scalable synthesis and the possibility of easily varying the flanking nitrogen donors of this pincer scaffold motivated us to prepare strictly T‐shaped phosphines based on this ligand framework. The work described herein aims to realize the phosphorus centered ambiphilic reactivity patterns predicted for such systems and evaluate their performance in small molecule activation reactions.

## Results and Discussion

### Synthesis of NNN ligated phosphines and phosphoranes

We synthesized the trisamine **1^Me^** in a multi‐step procedure, starting from 2,7,9,9‐tetramethyl‐9,10‐dihydroacridine in 79 % isolated yield.^[63]^

**Figure 2:**
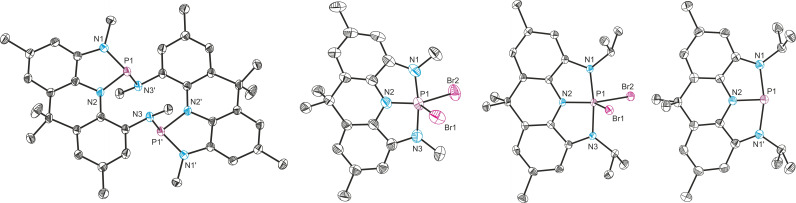
From left to right: single crystal X‐ray structures of **2**, **3^Me^**, **3** ^***i*** **Pr**^ and **4** ^***i*** **Pr**^. Thermal ellipsoids pictured at 50 % probability level. Hydrogen atoms and solvent of crystallization removed for clarity.

Incorporation of phosphorus was attempted by reacting **1^Me^** with PCl_3_ in the presence of NEt_3_ (Scheme 1). The ^31^P{^1^H} NMR spectrum of the reaction mixture reveals the formation of a major product at 112.1 ppm (49 % isolated yield). However, the ^1^H NMR spectrum shows that the formed product is not *C*_2*v*_ symmetric as would be expected for the targeted T‐shaped phosphine, but rather that it exhibits *C*_1_ symmetry on the NMR timescale. Single crystal X‐ray diffraction (SXRD) shows that the bisphosphine **2** was instead formed (Figure 2), which supports the recorded ^1^H NMR spectrum. **2** features a central 10‐membered ring linking both acridane fragments. The SXRD structure is consistent with retention of the phosphorus(III) redox state over the course of the reaction, in line with NMR spectroscopic analysis. **2** is likely formed by dimerization of a transient highly electrophilic T‐shaped phosphine, akin to reports by Chitnis, Wolf and co‐workers for NNN ligated antimony and bismuth pincer compounds and ONO ligated phosphines, respectively_._[^16^, ^64^]

To further substantiate this claim, we aimed to access the targeted species via reduction of an NNN ligated phosphorus(V) precursor. The dibromo‐phosphorane **3^Me^** can be accessed by reaction of the protio‐ligand **1^Me^** with PBr_5_ in the presence of NEt_3_ (Scheme 1, 64 % isolated yield). **3^Me^** is *C*_2*v*_ symmetric on the NMR timescale and features a phosphorus chemical shift of −107.6 ppm. This compares well with other recently reported dibromophosphoranes bearing pincer‐type ligands and is within the chemical shift region expected for a phosphorus(V) species.^[21]^ The P−N bond distances (1.653(2) and 1.768(2) Å) derived by single crystal X‐ray diffraction are in line with the formation of single bonds, suggesting no oxidation of the redox non‐innocent ligand framework (Figure 2).

When **3^Me^** is reacted with 1.5 equiv. PMe_3_ in benzene, a bright orange solution is generated and PMe_3_Br_2_ immediately precipitates out of the reaction mixture due to phosphine‐mediated dehalogenation of **3^Me^**.[^65^, ^66^, ^67^, ^68^, ^69^] A new resonance at 150.9 ppm is observed in the ^31^P{^1^H} NMR spectrum (Figure S59). This chemical shift (and color, see below) are as expected for geometrically constrained phosphines, allowing the tentative assignment of this species as T‐shaped phosphine **4^Me^** (Scheme 1). However, over the course of minutes the solution turns yellow and the aforementioned ^31^P{^1^H} NMR signal converts to that of diphosphine **2**.

The crystal structure of **2** suggests that a slight increase in the steric bulk of the flanking nitrogen substituents might be sufficient to stabilize a monomeric T‐shaped phosphine. Therefore, the previously reported trisamine **1** ^***i*** **Pr**^ (which possesses bulkier isopropyl substituents) was employed. However, reaction of **1** ^***i*** **Pr**^ with PCl_3_ in the presence of NEt_3_ resulted in the formation of numerous species as observed by ^31^P{^1^H} NMR spectroscopy (Figure S61). As a clean and direct synthesis of **4** ^***i*** **Pr**^ was not possible from PCl_3_, we sought to access a phosphorus(V) species which can, in turn, be reduced to the phosphine. Dibromophosphorane **3** ^***i*** **Pr**^ could be accessed in 54 % isolated yield upon reaction of **1** ^***i*** **Pr**^ with PBr_5_ and NEt_3_ (Scheme 1). The molecular structure of **3** ^***i*** **Pr**^ as determined by SXRD and ^1^H and ^31^P{^1^H} NMR spectroscopy closely resemble the data obtained for **3^Me^** (Figure 2). In stark contrast to the reactivity of **3^Me^** upon dehalogenation, treatment of **3** ^***i*** **Pr**^ with PMe_3_ or Mg powder results in the clean formation of the phosphine **4** ^***i*** **Pr**^ (90 % isolated yield) which exhibits increased stability in solution when compared to its smaller congener **4^Me^** (Scheme 1); after several days only traces of additional signals appear in the ^31^P{^1^H} NMR spectrum (<5 %, Figure S63). The ^31^P{^1^H} chemical shift (150.0 ppm) and the overall *C*_2*v*_ symmetry on the NMR timescale, as observed by ^1^H NMR spectroscopy, corroborate formation of a T‐shaped phosphine.[^29^, ^46^] The molecular structure of **4** ^***i*** **Pr**^ derived by SXRD reveals strict planarity and a buried volume of %V_Bur_=63 % around the phosphorus center (angle sum of 360°, Figure 2).^[70]^ Furthermore, the contraction of the C−N bond lengths when compared to **3** ^***i*** **Pr**^ indicate partial electromorphism stemming from the redox non‐innocent nature of the NNN pincer scaffold.[^34^, ^46^]

### Ambiphilic reactivity and two‐electron oxidation reactions

**Scheme 2:**
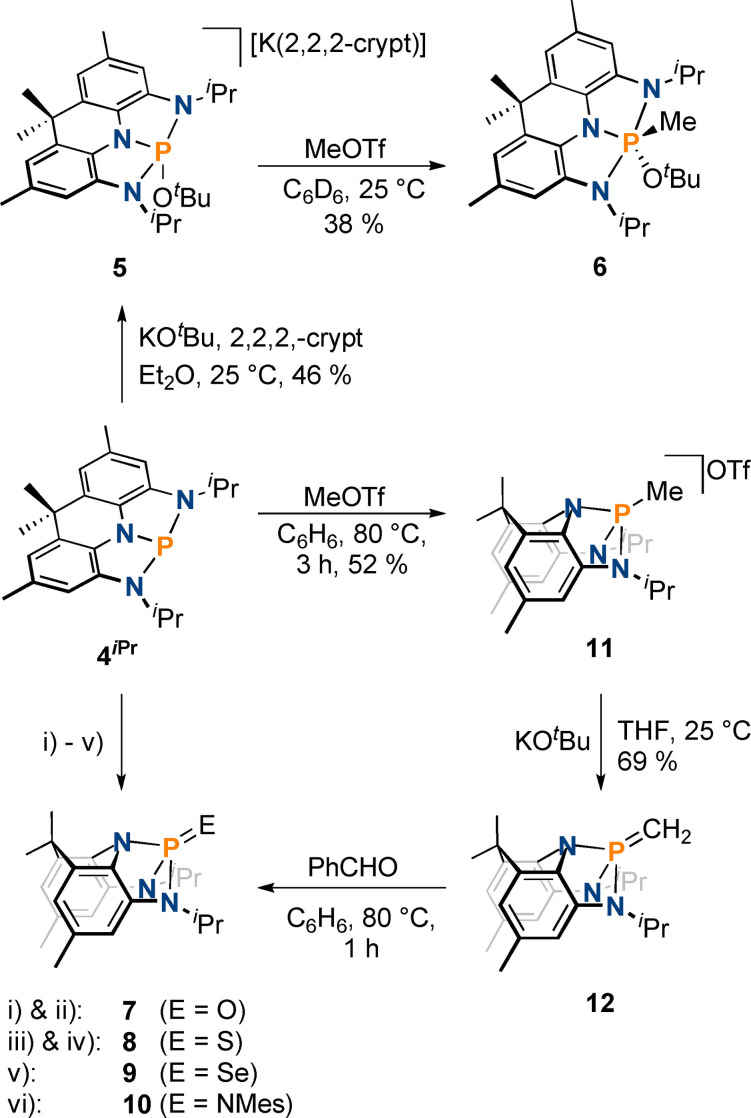
Electrophilic functionalization of **4** ^***i*** **Pr**^ to give **5** and subsequent methylation yielding **6**; Generation of phosphorus(V) chalcogenides **7–9**; nucleophilic reactivity of **4** ^***i*** **Pr**^ towards methyl triflate to yield **10**, its subsequent deprotonation to form **11**, and its conversion to **7**; i) xs. O_2_, THF, 60 °C, 16 h, 55 %; ii) 1 equiv. pyridine N‐oxide, C_6_D_6_, 25 °C, 16 h, 57 % isolated yield; iii) 5 equiv. S_8_, CD_2_Cl_2_, 25 °C, 4 h, observed spectroscopically; iv) 1 equiv. Lawesson's reagent, PhMe, 110 °C, 16 h, 96 %; v) 5 equiv. Se, CD_2_Cl_2_, 25 °C, 16 h, 54 % isolated yield; vi) 1 equiv. MesN_3_, PhMe, 110 °C, 2 h, 70 %.

**Figure 3:**
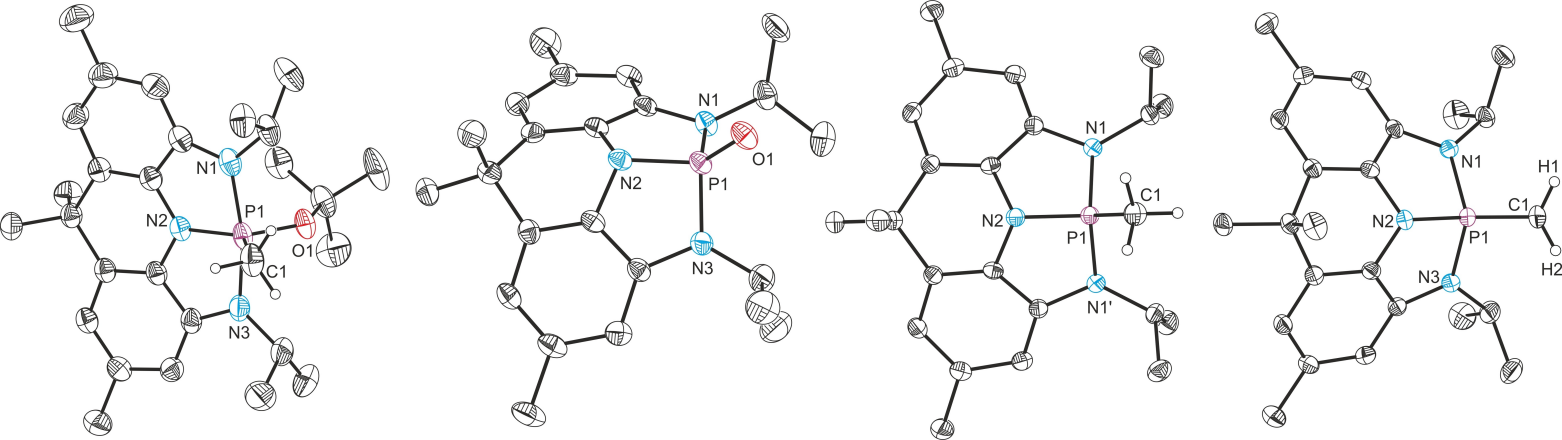
From left to right: single crystal X‐ray structures of **6**, **7**, **11** and **12**. Thermal ellipsoids pictured at 50 % probability level. Hydrogen atoms (except for those associated with C1), solvent of crystallization, and the OTf^−^ anion in **11** removed for clarity.

The non‐VSEPR structure of pincer type phosphines affords a low‐lying empty p‐type orbital situated on the phosphorus atom, enabling electrophilic reactivity.^[7]^ We aimed to address this property by formation of the anionic phosphorus alkoxide **5** upon reaction of **4** ^***i*** **Pr**^ with KO^*t*^Bu in the presence of 4,7,13,16,21,24‐hexaoxa‐1,10‐diazabicyclo[8.8.8]hexacosan (2,2,2‐crypt; Scheme 2, 46 % isolated yield). A low‐resolution single crystal X‐ray diffraction structure reveals that the planarity of the pincer ligand is maintained on coordination of the alkoxide to the phosphorus center (Figure S74). Unfortunately, the low quality of the crystals prevents a meaningful discussion of bond metric parameters. The ^31^P{^1^H} chemical shift of 50.0 ppm compares well with related anionic phosphorus alkoxides.^[20]^ We were interested if subsequent treatment with the strong electrophile MeOTf would result in phosphorus‐centered reactivity or functionalization of the ligand periphery as demonstrated by Aldridge, Goicoechea and co‐workers.^[20]^ Addition of MeOTf to **5** results in the instant formation of a new resonance at −39.4 ppm in the ^31^P{^1^H} NMR spectrum, indicative of phosphorus oxidation to phosphorus(V), reminiscent of recently described nucleophilic reactivity of a geometrically constrained phosphoranide.^[71] 1^H NMR spectroscopy and SXRD further confirm that the *C_s_* symmetric phosphorane **6** was formed (Scheme 2, Figure 3, 38 % isolated yield). NMR spectroscopic analysis of the reaction mixture gave no indications of competing methylation of the adjacent nitrogen atoms in stark contrast to previous findings.[^20^, ^51^]

An established reactivity pattern of transition metals towards chalcogens consists of formation of M=E double bonds accompanied by two electron oxidation of the metal center. This type of reactivity is surprisingly rare for phosphorus pincer compounds in the case of oxygen and sulfur, while selenophosphorane formation has been reported numerous times.[^8^, ^10^] Uhl and co‐workers demonstrated that the reaction of a phosphine ligated by a CCC pincer ligand with selenium and sulfur triggered P−C bond insertion reactions.^[31]^ Reaction of ONO and NPN pincer compounds towards oxygen and oxygen atom transfer reagents was reported to result in ligand disassembly and ligand oxidation, respectively.[^21^, ^51^] By contrast, a CCC ligated phosphenium cation showed phosphorus centered oxidation.^[33]^ When **4** ^***i*** **Pr**^ is reacted with oxygen at elevated temperatures, the phosphorus oxide **7** is formed as evidenced by ^31^P{^1^H} and ^1^H NMR spectroscopy (Scheme 2, ^31^P{^1^H} NMR=37.4 ppm, 55 % isolated yield). **7** can also be prepared upon reaction of **4** ^***i*** **Pr**^ with pyridine N‐oxide (57 % isolated yield). NMR spectroscopy further reveals that **7** exhibits *C_s_* symmetry on the NMR timescale. The molecular structure of **7** in the solid state shows that the presence of the P=O double bond (1.468(2) Å) leads to a significant geometric distortion of the acridane ligand backbone (Figure 3). Instead of being planar, the acridane ligand adopts a bent geometry to accommodate the tetrahedral phosphorus center which exhibits a τ′_4_ value of 0.76.^[72]^ The corresponding thiophosphorane **8** can be either accessed via reaction of **4** ^***i*** **Pr**^ with elemental sulfur or upon reaction of **7** with Lawesson's reagent (Scheme 2, 96 % isolated yield). Finally, the selenophosphorane **9** can be cleanly obtained upon reaction of **4** ^***i*** **Pr**^ with grey selenium (Scheme 2, 54 % isolated yield). Both transformations proceed with no indications of competing P−N insertion reactions. The measured coupling constant of ^1^ *J*_P‐Se_=919 Hz suggest a high s‐character of the phosphorus lone‐pair in the parent phosphine.^[73]^ This value however does not necessarily provide accurate information about the s‐character of the phosphorus lone pair present in **4** ^***i*** **Pr**^ since the acridane ligand heavily distorts over the course of the reaction. While the C−C and C−N bond lengths of the NNN ligand for **8** and **9** are almost identical to those of **7**, the P=E double bonds and P−N single bonds show a gradual increase upon descending group 16 (Figure 3). The ^31^P{^1^H} chemical shifts show significant differences, (**7**: 37.4 ppm; **8**: 83.8 ppm, **9**: 75.7 ppm) while the ^1^H NMR signals are almost unchanged.

**Scheme 3:**
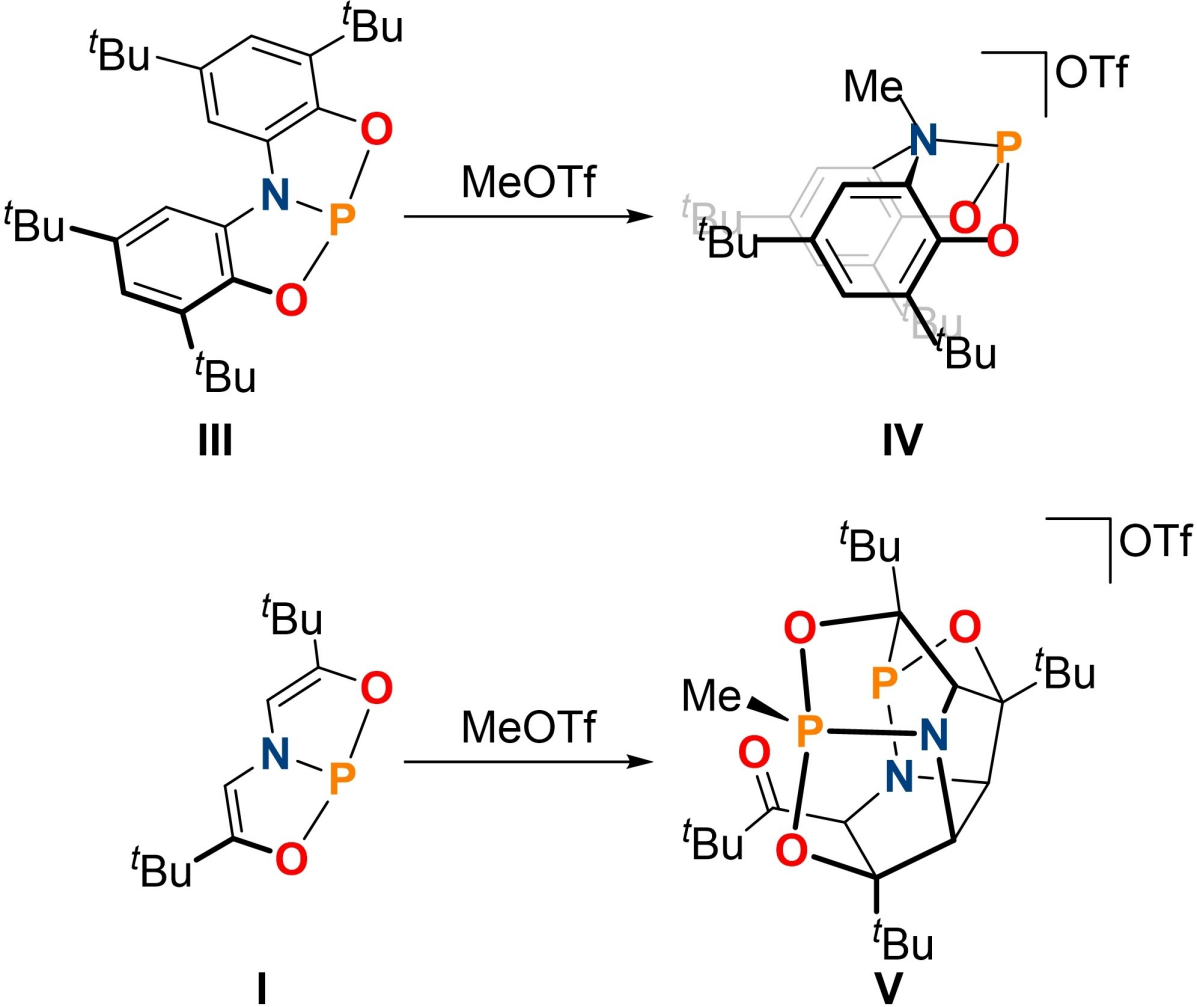
Previously reported ligand centered (top) and domino reactivity (bottom) of the phosphorus pincer compounds **I** and **III** upon reaction with MeOTf.

Two‐electron oxidation of the phosphorus center was also observed upon reaction with MesN_3_ (Mes=2,4,6‐Me_3_‐C_6_H_2_) to give the iminophosphorane **10** in 70 % isolated yield (^31^P{^1^H} NMR 18.1 ppm, Scheme 3). As with **7–9**, a geometrical distortion of the ligand backbone towards a bent shape is observed. The P=NMes bond length of 1.533(1) Å compares well with structurally related geometrically constrained iminophosphoranes.^[39]^

Motivated by this phosphorus centered reactivity, particularly the formation of **6** through subsequent reaction with nucleophilic/electrophilic reagents, we investigated whether **6** can also be obtained via the reverse addition of these reagents. Namely, subsequent reaction of **4** ^***i*** **Pr**^ with MeOTf followed by addition of KO^t^Bu. It has been shown that treatment of geometrically constrained phosphines with strong electrophiles such as MeOTf resulted in ligand functionalization as was the case with bent (*C_s_* symmetric) phosphine **III** and T‐shaped (*C*_2*v*_ symmetric) **I** (Scheme 3).[^20^, ^25^, ^51^]

In the latter case, initial phosphorus‐centered reactivity was followed by disintegration of the pincer framework to provide **V**; a detrimental reactivity pathway that we aimed to avoid using the more chemically inert acridane backbone of **4** ^***i*** **Pr**^_._ In stark contrast, **4** ^***i*** **Pr**^ reacts cleanly with MeOTf at elevated temperatures to give the phosphonium cation **11** as indicated by multinuclear NMR spectroscopy and single crystal X‐ray diffraction (^31^P{^1^H} NMR 80.5 ppm, Scheme 2, Figure 3, 52 % isolated yield). As observed for compounds **7–9**, the NNN pincer scaffolds exhibits a high degree of distortion to accommodate the cationic phosphorus center. This is accompanied by a significant shortening of the P−N bonds which lie between the values measured for of **4** ^***i*** **Pr**^ and **7**. We attribute this unique electrophilic phosphorus‐centered reactivity to the effective quenching of the nucleophilicity of the adjacent nitrogen due to the high rigidity of the acridane ligand framework (and thus delocalization of the nitrogen lone pairs).

Interestingly, reacting **11** with KO^*t*^Bu does not give **6** but rather yields a new product with a ^31^P{^1^H} NMR chemical shift of 61.2 ppm. The presence of a new resonance in the ^1^H NMR spectrum at 1.20 ppm displaying strong coupling to phosphorus (^2^ *J*_H‐P_=18.5 Hz) confirms the formation of the geometrically constrained ylid **12**. This is further corroborated by single crystal X‐ray diffraction (Scheme 2, 69 % isolated yield, Figure 3). To accommodate the ylid functionality, the acridane ligand maintains its bowl shape (Figure 3). The ylidic P−CH_2_ bond length (1.638(2) Å) compares well with other reported acyclic ylids,[^74^, ^75^] and is notably shorter than the P−CH_3_ bond in **11** (1.778(2) Å), lying between the expected values for single and double bonds. The typical reactivity of phosphorus ylids is maintained upon geometric constraint as evidenced by reaction with benzaldehyde (Scheme 2); ^31^P{^1^H} NMR spectroscopy reveals the formation of phosphine oxide **7**, and the ^1^H NMR spectrum displays signals corresponding to styrene (Figures S67 and S68) along with a mixture of unknown secondary products.

### Reactivity towards E‐H bonds

**Scheme 4:**
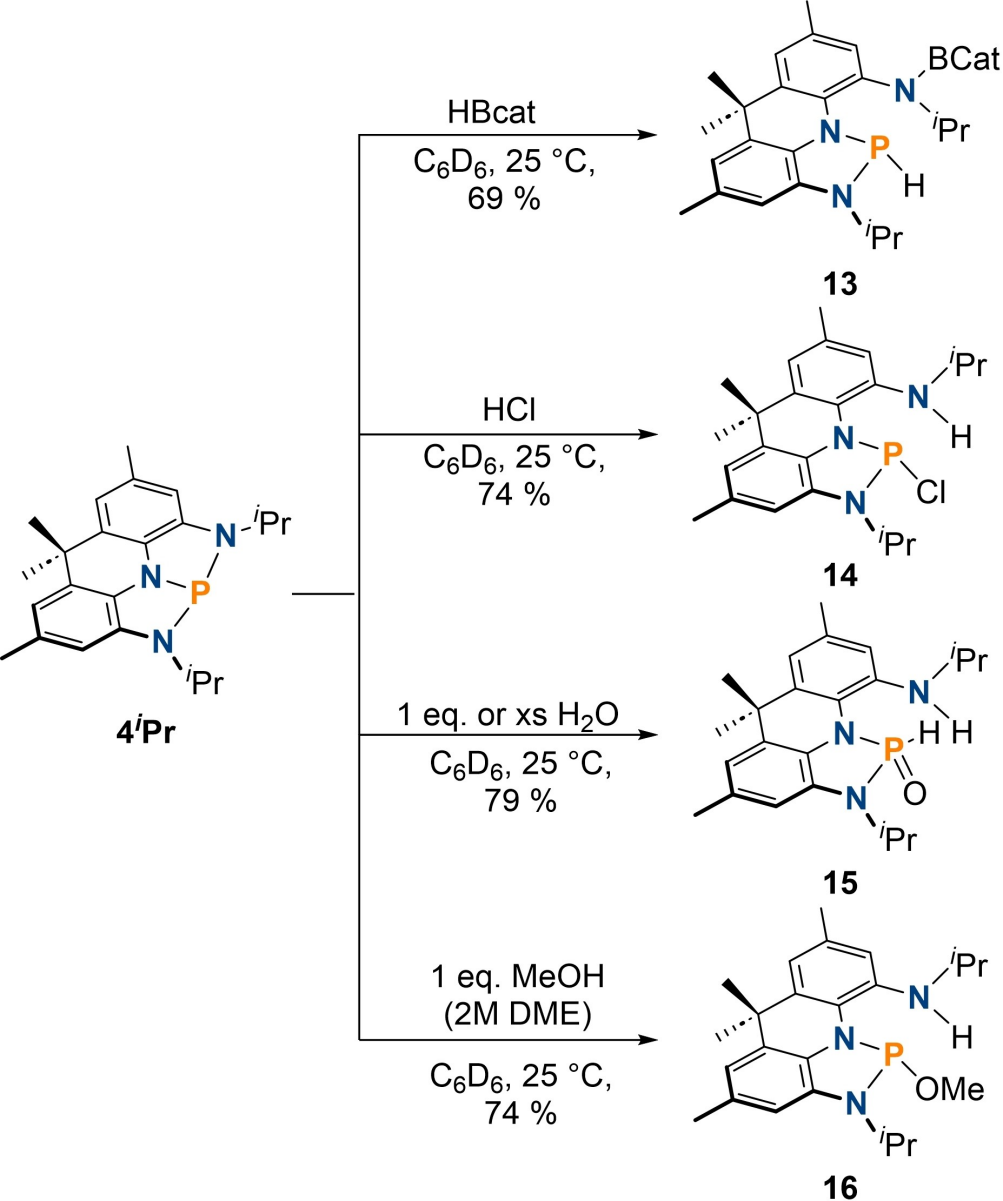
Reactivity of **4** ^***i*** **Pr**^ towards hydridic and protic E−H bonds to generate **13** (only one isomer shown), **14**, **15** and **16**.

Having established the phosphorus‐centered reactivity and accessibility of phosphorus(V) species derived from **4** ^***i*** **Pr**^, we set out to evaluate its reactivity towards hydridic and protic E−H bonds. Transition metal pincer complexes are well known to undergo redox neutral, cooperative substrate activation,[^76^, ^77^, ^78^, ^79^, ^80^, ^81^, ^82^] while pincer type phosphines tend to favor oxidative addition product formation due to the high stability of the phosphorus(V) redox state.[^6^, ^7^, ^8^, ^9^, ^10^] Reacting **4** ^***i*** **Pr**^ with catecholborane (HBcat) results in the formation of two new signals in the ^31^P{^1^H} NMR spectrum at 67.1 ppm and 61.8 ppm in a 1:0.6 ratio. These signals display doublet of doublet multiplicity, with a large coupling of ^1^ *J*_H‐P_=135 and 148 Hz for the major and minor isomers respectively, and a smaller coupling of ^3^ *J*_H‐P_=11 and 12 Hz corresponding to ligand protons. This multiplicity, along with ^1^H NMR spectroscopy suggests that cooperative addition along a P−N bond occurred producing two isomers of **13** containing a P−H bond (Scheme 4). This compares well with prior results obtained for bent NNN pincer compounds.[^40^, ^41^] Heating a solution of **13** in benzene at 80 °C for several days does not result in the formation of phosphorus(V) products, but rather in formation of PH_3_ and **4** ^***i*** **Pr**^ suggesting that formal oxidative addition of HBcat is thermodynamically unfavored (Figure S70). Despite numerous attempts, suitable crystals for X‐ray diffraction could not be obtained.

**Figure 4:**
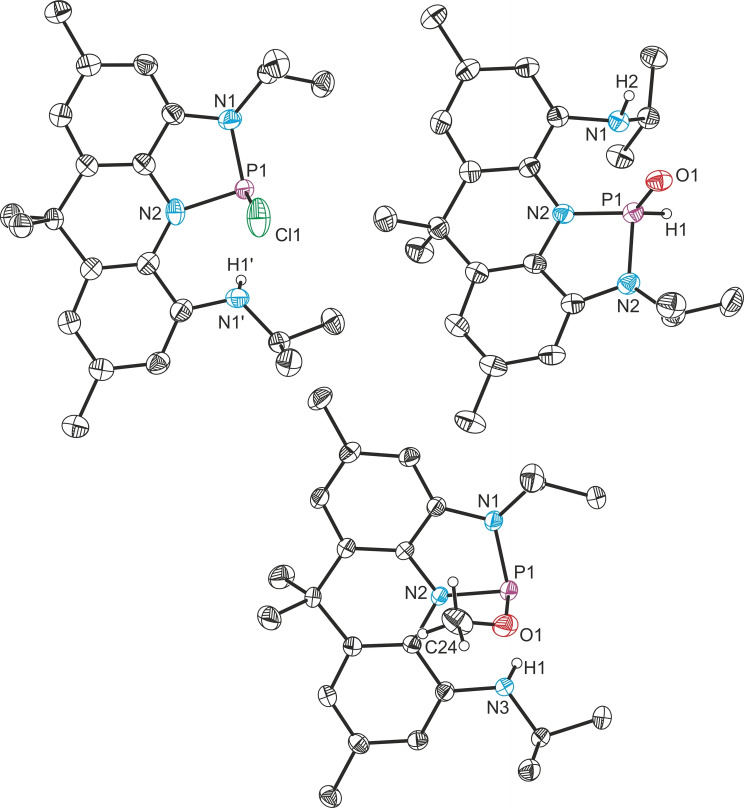
Clockwise from top left: single crystal X‐ray structures of **14**, **15** and **16**. Thermal ellipsoids pictured at 50 % probability level. Hydrogen atoms (except for those associated with activated substrates) removed for clarity.

Regarding reactivity with protic E−H bonds, the reaction of **4** ^***i*** **Pr**^ with one equivalent ethereal HCl results in clean conversion to a single new product as observed by ^31^P{^1^H} NMR spectroscopy (130.6 ppm, 74 % isolated yield). This distinct chemical shift and the presence of a highly asymmetric ligand environment indicated by ^1^H NMR spectroscopy suggests oxidative addition did not take place bu rather that redox neutral substrate activation upon scission of a P−N bond occurred (Scheme 4). Single crystal X‐ray diffraction corroborates these results and reveals that the reaction yielded the chlorophosphine **14** under retention of the planarity of the acridane scaffold (Figure 4). Extended heating of solutions of **14** do not result in formation of phosphorus(V) products confirming that **14** is the thermodynamically favored reaction product.

Since cooperative activation of E−H bonds along the side‐arm P−N bond of **4** ^***i*** **Pr**^ appears to be the favored mode of substrate activation, we were interested if E−H bond activation can further lead to phosphorus oxidation when multiple bonds of a substrate are cleaved. Consequently, we chose water as a simple substrate containing two acidic O−H bonds. Arduengo reported that **I** undergoes complete disassembly upon reaction with water resulting in the formation of H_3_PO_3_ and the fully protonated H_3_ONO ligand.^[29]^ In contrast, Aldridge, Goicoechea and co‐workers demonstrated oxidative addition of water to the bent ONO ligated phosphine **III**, yielding a hydroxo hydrido phosphorane.^[19]^ When **4** ^***i*** **Pr**^ is exposed to an excess or one equivalent of water, formation of a new species in the ^31^P{^1^H} NMR spectrum at 1.3 ppm is observed suggesting the formation of a phosphorus(V) species. Furthermore, the signal is split into a doublet of doublets with observed coupling constants of ^1^ *J*_H‐P_=715 Hz and ^3^ *J*_H‐P_=18 Hz indicative of the presence of a P−H bond. ^1^H NMR spectroscopy (δ_H_=9.42 ppm) confirms this assignment. A broad singlet resonance in the ^1^H NMR spectra at 3.85 ppm also indicated the formation of an N−H bond. The reaction product has an unsymmetrical ^1^H NMR spectra suggesting *C*_1_ symmetry which is not in line with simple oxidative addition of water by the phosphorus center. Characteristic bands for N−H (3278 cm^−1^) and P−H (2441 cm^−1^) stretching vibrations are observed in the IR spectrum.

Single crystal X‐ray diffraction shows that the phosphorus(V) oxide **14** was formed (Scheme 4, Figure 4, 79 % isolated yield). The P=O bond length (1.4827(13) Å) is comparable with that present in **7** (cf. 1.468(2) Å). This represents a rare example of complete H_2_O disassembly at a pincer‐type phosphine to form a P(O)HR_2_ species utilizing the ambiphilic reactivity of **4** ^***i*** **Pr**^.[^11^, ^19^, ^83^, ^84^, ^85^] Reaction of **4** ^***i*** **Pr**^ with D_2_O leads to a disappearance of the P−H and N−H resonances in the ^1^H NMR spectra. The formation of **15** over a formal oxidative addition of water is likely to be attributed to initial cooperative splitting of an O−H bond, a reactivity typically observed for NNN phosphorus pincer compounds.[^26^, ^28^, ^45^, ^47^, ^50^, ^55^]

Consequently, we propose initial water coordination towards the highly electrophilic phosphorus center and subsequent deprotonation by the flanking nitrogen atom to afford a transient phosphinous acid. Tautomerization of R_2_POH derivatives towards the corresponding oxides (to produce the final product **15** in this case) is well established if these species are not stabilized by strongly electron withdrawing ligands.[^86^, ^87^, ^88^, ^89^, ^90^, ^91^, ^92^] To probe this mechanism, **4** ^***i*** **Pr**^ is reacted with MeOH – a substrate with only one available O−H bond. Cooperative addition along a flanking P−N bond is observed to give the phosphorus(III) alkoxide **16** (^31^P{^1^H} NMR: 97.3 ppm, Scheme 4, Figure 4, 74 % isolated yield), in line with the mechanistic proposal of water activation. As in all other cases of redox neutral substrate activation, the overall planarity of the acridane ligand scaffold is maintained as evidenced by SCXRD.

## Conclusion

We have reported the synthesis of the highly reactive, strictly T‐shaped phosphine **4** ^***i*** **Pr**^ ligated by a NNN pincer ligand. This was enabled by careful steric tuning of the employed acridane ligand scaffold. **4** ^***i*** **Pr**^ exhibits pronounced ambiphilicity as evidenced by phosphorus centered reactivity towards nucleophiles and electrophiles. Furthermore, clean two electron oxidation upon reaction with chalcogens and formation of heavily distorted tetrahedral phosphorus(V) species featuring single, double and ylidic bonds was demonstrated. Finally, the reactivity of **4** ^***i*** **Pr**^ towards hydridic and protic E−H bonds was evaluated which proceeded under retention of the phosphorus(III) redox state in case of HBcat, HCl and methanol. Finally, sequential O−H bond scission of a water molecule was observed, demonstrating both ligand‐cooperative bond scission and phosphorus oxidation at a single substrate. Further studies will be directed to utilize this platform for catalytic applications.

## Experimental Section

For spectra and more detailed spectral assignments, please refer to the Supporting Information document.

**Synthetic methods**: All reactions and product manipulations were carried out using standard Schlenk‐line techniques under an inert atmosphere of argon, or in a dinitrogen filled glovebox (MBraun UNIlab glovebox maintained at <0.1 ppm H_2_O and <0.1 ppm O_2_). Hexane (Sigma Aldrich, HPLC grade), pentane (Sigma Aldrich, HPLC grade), benzene (Rathburn, HPLC grade), dichloromethane (Sigma Aldrich, HPLC grade), acetonitrile (Sigma Aldrich, HPLC grade) diethyl ether (Sigma Aldrich, HPLC grade) and toluene (Sigma Aldrich, HPLC grade) were purified using an MBraun SPS‐800 solvent system. Ethyl acetate (Sigma Aldrich, HPLC grade) and methanol (Sigma Aldrich anhydrous, 99.8 %) was used as provided. THF (Sigma Aldrich, HPLC grade) was distilled over sodium/benzophenone. C_6_D_6_ (Sigma Aldrich, 99.5 %) and CD_2_Cl_2_ (Sigma Aldrich, 99.5 %) was degassed and dried over CaH_2_. *d*_8_‐THF (Sigma Aldrich, 99.5 %) was degassed and dried over sodium/potassium alloy. All dry solvents were stored under argon in gas‐tight ampoules over activated 3 Å molecular sieves. 2,7,9,9‐tetramethyl‐4,5‐dinitro‐9,10‐dihydroacridine,^[62]^ **1** ^***i*** **Pr**[62]^ and mesityl azide^[93]^ were synthesised according to their referenced literature procedures. The following were used as received from their commercial supplier without further purification: palladium on carbon (10 w%, Sigma Aldrich), H_2_ (Sigma Aldrich), paraformaldehyde (Merck), sodium methoxide (Sigma Aldrich), sodium borohydride (Sigma Aldrich), phosphorus pentabromide (Acros), trimethylphosphine (Strem), magnesium powder (Sigma Aldrich), potassium *tert*‐butoxide (Sigma Aldrich), 4,7,13,16,21,24‐hexaoxa‐1,10‐diazabicyclo[8.8.8]hexacosane (Merck), methyl triflate (Apollo Scientific), oxygen (Linde), 2,4‐bis(4‐methoxyphenyl‐1,3,2,4‐dithiadiphosphetane‐2,4‐disulfide (Alfa Aesar), sulfur (Sigma Aldrich), selenium (Sigma Aldrich), 2*H*‐1,3,2‐benzodioxaborole (Alfa Aesar), 2 M hydrochloric acid in diethyl ether (diluted prior to use, Alfa Aesar) and deuterium oxide (Sigma Aldrich). Phosphorus trichloride (Sigma Aldrich) was distilled and degassed prior to use. Triethylamine (TCI) was degassed, dried over CaH_2_ and distilled prior to use. Benzaldehyde (Sigma Aldrich) was distilled prior to use. Pyridine N‐oxide (Sigma Aldrich) was sublimed prior to use.

**Characterisation techniques**: NMR samples were prepared inside an inert atmosphere glovebox in NMR tubes fitted with a gas‐tight valve. NMR spectra were acquired on either a Bruker AVIII 400 MHz NMR spectrometer (^1^H 400 MHz, ^11^B 128 MHz, ^31^P 162 MHz), a Bruker AVIII 500 spectrometer (^1^H 500 MHz, ^31^P 202 MHz, ^19^F 470 MHz, ^77^Se 95 MHz) or a Bruker Avance NEO 600 MHz NMR spectrometer with a broadband helium cryoprobe (^1^H 600 MHz, ^31^P 243 MHz, ^13^C 151 MHz). ^1^H and ^13^C NMR spectra were referenced to their respective solvent resonance (^1^H NMR C_6_D_6_: δ=7.16 ppm, CD_2_Cl_2_: δ=5.32 ppm, *d*_8_‐THF: δ=3.58 ppm; ^13^C NMR C_6_D_6_: δ=128.1 ppm, CD_2_Cl_2_: δ=53.8 ppm, *d*_8_‐THF: δ=67.6 ppm). ^11^B NMR spectra were externally referenced to BF_3_⋅Et_2_O in C_6_D_6_. ^19^F NMR spectra were externally referenced to CFCl_3_. ^31^P NMR spectra were externally referenced to an 85 % solution of H_3_PO_4_ in H_2_O. High‐resolution mass spectrometry was acquired using a Thermo Exactive High‐Resolution Orbitrap FTMS instrument equipped with a Acquity TUV Detector, with a Waters Acquity Ultraperformance LC system used for sample delivery. Elemental analyses were carried out by Elemental Analysis Services Team (London Metropolitan University, U.K.). Samples (approx. 5 mg) were submitted in flame sealed glass tubes. Infrared spectra were acquired on a Thermo Scientific iS5 FTIR spectrometer using an iD3 ATR stage.

**Synthesis of 1^Me^**: 2,7,9,9‐tetramethyl‐4,5‐dinitro‐9,10‐dihydroacridine (663 mg, 2.03 mmol, 1.00 equiv.) and palladium on carbon (108 mg, 0.102 mmol palladium, 10.0 wt.% palladium, 5.00 mol % palladium) are added to a gas tight ampoule and suspended in ethyl acetate (20 mL). The suspension is freeze‐pump‐thaw degassed four times, then refilled with dihydrogen gas (1 atm, excess) and stirred at 50 °C for 18 h. The mixture is cooled to room temperature and the solvent is removed *in vacuo*. The residue is extracted into methanol (3×10 mL) and filtered. The mixture is cooled to 0 °C then paraformaldehyde (608 mg, 20.6 mmol, 10.0 equiv.) is added followed by sodium methoxide (25 w% in methanol, 1.85 mL, 8.10 mmol, 4.00 equiv.). The mixture is warmed to reflux and stirred for 1 h. The mixture is then cooled to 0 °C and sodium borohydride (843 mg, 22.3 mmol, 11.0 equiv.) is added in three portions over the course of 15 min. The mixture is warmed to reflux and stirred for 2 h. The mixture is cooled to room temperature and the solvent is removed *in vacuo*. The residue is extracted into benzene (3×10 mL) and filtered. Lyophilisation from a concentrated benzene solution affords **1^Me^** (474 mg, 1.61 mmol, 79 %) as a white powder. ^1^H NMR (600 MHz, C_6_D_6_): δ=6.94 (2H, s, Ar), 6.46 (2H, s, Ar), 5.69 (1H, br s, NH), 2.55 (2H, br m, NH), 2.46 (6H, d, ^3^ *J*_H‐H_=5.5 Hz, NCH_3_), 2.36 (6H, s, ArCH_3_), 1.73 ppm (6H, s, CH_3_). ^13^C{^1^H} NMR (151 MHz, C_6_D_6_): δ=136.3 (s, Ar), 129.7 (s, Ar), 129.2 (s, Ar), 126.7 (s, Ar), 117.5 (s, Ar), 111.3 (s, Ar), 36.2 (s, *C*(CH_3_)_2_), 31.9 (s, CH_3_), 31.1 (s, NCH_3_), 21.5 ppm (s, ArCH_3_). HRMS (ESI) *m/z* calcd. for C_19_H_25_N_3_+H^+^: 296.2121 [*M*+H]^+^; found: 296.2116.

**Synthesis of 2**: **1^Me^** (50.0 mg, 0.169 mmol, 1.00 equiv.) is dissolved in dichloromethane (5 mL) and cooled to −78 °C. Phosphorus trichloride (0.573 M in hexane, 0.30 mL, 0.169 mmol, 1.00 equiv.) is added followed by triethylamine (83.0 μL, 0.593 mmol, 3.50 equiv.). The mixture is gradually warmed to room temperature and stirred for 16 h, resulting in the generation of a pale‐yellow solution and off‐white precipitate. The solution is then filtered, and the precipitate extracted with dichloromethane (3×2 mL). The solvent is removed from the combined extracts *in vacuo* and the resulting residue washed with acetonitrile (3×2 mL) at −30 °C. The product is dried under vacuum, providing **2** as a white powder (26.0 mg, 41.1 μmol, 49 %).^1^H NMR (600 MHz, C_6_D_6_): δ=7.19 (1H, s, Ar), 6.85 (1H, s, Ar), 6.72 (1H, s, Ar), 6.26 (1H, s, Ar), 2.79 (6H, d, ^3^ *J*_P‐H_=8.0 Hz, NCH_3_), 2.63 (6H, d, ^3^ *J*_P‐H_=1.3 Hz, NCH_3_), 2.41 (6H, s, ArCH_3_), 2.24 (6H, s, ArCH_3_), 1.78 (6H, s, CH_3_), 1.65 ppm (6H, s, CH_3_). ^1^H{^31^P} NMR (500 MHz, C_6_D_6_): as above, except δ=2.79 (6H, s, NCH_3_), 2.63 ppm (6H, s, NCH_3_). ^13^C{^1^H} NMR (151 MHz, C_6_D_6_): δ=139.2 (d, ^2^ *J*_C‐P_=7.9 Hz, Ar), 133.8 (dd, ^2^ *J*_C‐P_=5.2 Hz, ^3^ *J*_C‐P_=2.7 Hz, Ar), 133.2 (m, *C*_Ar_), 131.0 (s, Ar), 129.9 (d, ^4^ *J*_C‐P_=2.5 Hz, Ar), 129.2 (d, ^2^ *J*_C‐P_=8.2 Hz, Ar), 127.5 (d, ^3^ *J*_C‐P_=7.0 Hz, Ar), 126.3 (d, ^4^ *J*_C‐P_=2.6 Hz, Ar), 126.2 (s, *C*_Ar_), 115.3 (s, Ar), 105.4 (s, Ar), 36.8 (s, *C*(CH_3_)_2_), 36.6 (s, CH_3_), 34.7 (dd, ^5TS^ *J*_C‐P_=5.5 Hz, ^2^ *J*_C‐P_=13.7 Hz, NCH_3_), 32.1 (d, ^2^ *J*_C‐P_=32.2 Hz, NCH_3_), 31.9 (s, CH_3_), 22.1 (s, ArCH_3_), 21.0 ppm (s, ArCH_3_). ^31^P NMR (243 MHz, C_6_D_6_): δ=112.1 ppm (m). ^31^P{^1^H} NMR (162 MHz, C_6_D_6_): δ=112.1 ppm (s). Elemental analysis calcd. (%) for C_38_H_44_N_6_P_2_: C 70.57, H 6.86, N 12.99; found: C 70.63, H 7.01, N 11.45.

**Synthesis of 3^Me^**: Phosphorus pentabromide (72.9 mg, 0.169 mmol, 1.00 equiv.) is dissolved in toluene (10 mL) and cooled to −78 °C. Triethylamine (71.0 μL, 0.507 mmol, 3.00 equiv.) is added. A solution of **1^Me^** (50 mg, 0.169 mmol, 1.00 equiv.) in toluene (10 mL) is also cooled to −78 °C and added dropwise to the phosphorus pentabromide solution. The mixture is gradually warmed to room‐temperature and stirred for 16 h resulting in the generation of an orange solution and off‐white precipitate. The solution is then filtered, and the precipitate extracted with toluene (3×5 mL). The solvent is removed *in vacuo* and the residue dissolved in hexane and cooled to −30 °C for 5 days resulting in the generation of orange solid (an unknown impurity). The solution is then filtered, and the solvent removed *in vacuo*. Lyophilisation from a concentrated benzene solution provides **3^Me^** as an orange powder (52.0 mg, 0.108 mmol, 64 %). ^1^H NMR (600 MHz, C_6_D_6_): δ=6.67 (2H, s, Ar), 6.12 (2H, s, Ar), 2.95 (6H, d, ^3^ *J*_P‐H_=21.1 Hz, NCH_3_), 2.26 (6H, s, ArCH_3_), 1.51 ppm (6H, s, CH_3_). ^1^H{^31^P} NMR (500 MHz, C_6_D_6_): as above, expect δ=2.95 ppm (6H, s, NCH_3_). ^13^C{^1^H} NMR (151 MHz, C_6_D_6_): δ=135.1 (d, ^2^ *J*_C‐P_=12.2 Hz, Ar), 133.6 (s, Ar), 129.7 (d, ^3^ *J*_C‐P_=9.2 Hz, Ar), 116.5 (d, ^4^ *J*_C‐P_=1.8 Hz, Ar), 116.1 (d, ^2^ *J*_C‐P_=13.4 Hz, Ar), 105.4 (d, ^3^ *J*_C‐P_=7.6 Hz, Ar), 36.9 (d, ^4^ *J*_C‐P_=1.7 Hz, *C*(CH_3_)_2_), 32.6 (s, CH_3_), 31.5 (d, ^2^ *J*_C‐P_=5.5 Hz, NCH_3_), 22.6 ppm (s, ArCH_3_). ^31^P NMR (243 MHz, C_6_D_6_): δ=−107.5 ppm (sept, ^3^ *J*_P‐H_=21.1 Hz). ^31^P{^1^H} NMR (162 MHz, C_6_D_6_): δ=−107.6 ppm (s). Elemental analysis calcd. (%) for C_19_H_22_Br_2_N_3_P: C 47.23, H 4.59, N 8.70; found: C 47.32, H 4.94, N 8.03.

**Synthesis of 3** ^***i*** **Pr**^: A solution of phosphorous pentabromide (366 mg, 0.853 mmol, 1.00 equiv.) in toluene (10 mL) is cooled to −78 °C. Triethylamine (0.600 mL, 4.27 mmol, 5.00 equiv.) is added, followed by the addition of a −78 °C solution of **1** ^***i*** **Pr**^ (300 mg, 0.853 mmol, 1.00 equiv.) in toluene (10 mL). The resulting bright green solution is gradually warmed to room temperature and stirred for 16 h over which the solution turned dark brown along with the precipitation of a light brown solid. The mixture is filtered, and the precipitate extracted with toluene (3×5 mL). The solvent is removed *in vacuo* providing an orange‐brown residue, which is then washed with diethyl ether (3×5 mL) at −78 °C. The resulting bright orange powder is dried *in vacuo* to provide **3** ^***i*** **Pr**^ (250 mg, 0.464 mmol, 54 %). ^1^H NMR (500 MHz, C_6_D_6_): δ=6.64 (2H, s, Ar), 6.62 (2H, s, Ar), 4.74 (2H, d‐sept, ^3^ *J*_P‐H_=27.3 Hz, ^3^ *J*_H‐H_=6.9 Hz, NCH), 2.26 (6H, s, ArCH_3_), 1.53 (12H, d, ^3^ *J*_H‐H_=6.9 Hz, NC(CH_3_)), 1.52 ppm (6H, s, CH_3_). ^1^H{^31^P} NMR (500 MHz, C_6_D_6_): as above, except δ=4.74 ppm (2H, sept, ^3^ *J*_H‐H_=6.9 Hz, NCH). ^13^C{^1^H} NMR (151 MHz, C_6_D_6_): δ=133.0 (d, ^4^ *J*_C‐P_=1.7 Hz, Ar), 132.7 (d, ^2^ *J*_C‐P_=13.7 Hz, Ar), 130.4 (d, ^3^ *J*_C‐P_=9.4 Hz, Ar), 116.7 (d, ^2^ *J*_C‐P_=14.3 Hz, Ar), 115.8 (m, Ar), 107.9 (d, ^3^ *J*_C‐P_=9.9 Hz, Ar), 46.7 (d, ^2^ *J*_C‐P_=6.0 Hz, NCH), 36.7 (d, ^4^ *J*_C‐P_=1.8 Hz, *C*(CH_3_)_2_), 33.0 (s, CH_3_), 22.7 (s, ArCH_3_), 19.0 ppm (d, ^3^ *J*_C‐P_=6.5 Hz, NC(*C*H_3_)). ^31^P NMR (202 MHz, C_6_D_6_): δ=−120.1 ppm (t, ^3^ *J*_P‐H_=27.3 Hz). ^31^P{^1^H} NMR (162 MHz, C_6_D_6_): δ=−120.1 ppm (s). Elemental analysis calcd. (%) for C_23_H_30_Br_2_N_3_P: C 51.22, H 5.61, N 7.79; found: C 51.08, H 5.26, N 7.41.

**Synthesis of 4** ^***i*** **Pr**^: **3** ^***i*** **Pr**^ (200 mg, 0.371 mmol, 1.00 equiv.) is dissolved in toluene (10 mL). A solution of trimethyl‐phosphine (0.19 mL, 1.85 mmol, 5.00 equiv.) in toluene (1 mL) is added and the mixture stirred for 15 min, resulting in the generation of a bright orange solution and off‐white precipitate. The solution is filtered and the precipitate extracted with toluene (3×5 mL). The solvent is removed *in vacuo*. Lyophilisation from a concentrated benzene solution provides **4** ^***i*** **Pr**^ as an orange powder (127 mg, 0.335 mmol, 90 %). **Alternative procedure**: **3** ^***i*** **Pr**^ (100 mg, 0.185 mmol, 1.00 equiv.) and magnesium powder (92 mg, 3.709 mmol, 20.0 equiv.) is dissolved in tetrahydrofuran (5 mL) and stirred at room temperature for 15 min. The solvent is removed *in vacuo* and the product extracted into pentane (3×2 mL) and filtered. The solvent is removed *in vacuo* from the combined extracts. Lyophilisation from a concentrated benzene solution provides **4** ^***i*** **Pr**^ as an orange powder (63.0 mg, 0.166 mmol, 90 %). ^1^H NMR (500 MHz, C_6_D_6_): δ=6.86 (2H, s, Ar), 6.79 (2H, s, Ar), 4.42 (2H, apparent sept, ^3^ *J*_H‐H_=6.9 Hz, NCH), 2.37 (6H, s, ArCH_3_), 1.72 (6H, s, CH_3_), 1.52 ppm (12H, d, ^3^ *J*_H‐H_=6.9 Hz, NC(CH_3_)). ^1^H{^31^P} NMR (500 MHz, C_6_D_6_): as above. ^13^C{^1^H} NMR (151 MHz, C_6_D_6_): δ=135.9 (d, ^2^ *J*_C‐P_=12.6 Hz, Ar), 133.5 (d, ^3^ *J*_C‐P_=4.6 Hz, Ar), 133.4 (s, Ar), 130.5 (d, ^2^ *J*_C‐P_=11.2 Hz, Ar), 115.7 (s, Ar), 109.1 (s, Ar), 45.1 (d, ^2^ *J*_C‐P_=20.9 Hz, NCH), 38.3 (s, *C*(CH_3_)_2_), 31.3 (s, CH_3_), 23.6 (d, ^3^ *J*_C‐P_=6.4 Hz, NC(*C*H_3_)), 22.6 ppm (s, ArCH_3_). ^31^P NMR (202 MHz, C_6_D_6_): δ=145.0 ppm (br s). ^31^P{^1^H} NMR (162 MHz, C_6_D_6_): δ=as above. Elemental analysis calcd. (%) for C_23_H_30_N_3_P: C 72.80, H 7.97, N 11.07; found: C 72.35, H 7.79, N 10.21.

**Synthesis of 5**: Potassium *tert*‐butoxide (3.1 mg, 27.8 μmol, 1.00 equiv.) and 2,2,2‐crypt (10.5 mg, 27.8 μmol, 1.00 equiv.) is dissolved in diethyl ether (0.5 mL). The resulting solution is added to **4** ^***i*** **Pr**^ (10.5 mg, 27.8 μmol, 1.00 equiv.) in a J. Young NMR tube producing a yellow solution. The solution is filtered, and half of the solvent is removed *in vacuo*. Crystallisation from this concentrated diethyl ether at room temperature yields **5** (11.0 mg, 12.7 μmol, 46 %) as yellow crystals. ^1^H NMR (500 MHz, *d*_8_‐THF): δ=5.82 (2H, s, Ar), 5.57 (2H, s, Ar), 3.70 (2H, apparent sept, ^3^ *J*_H‐H_=6.6 Hz, NCH), 3.37 (14H, s, crypt), 3.32 (14H, t, ^3^ *J*_H‐H_=4.6 Hz, crypt), 2.36 (14H, t, ^3^ *J*_H‐H_=4.6 Hz, crypt), 2.11 (6H, s, ArCH_3_), 1.60 (3H, s, CH_3_), 1.50 (3H, s, CH_3_), 1.41 (6H, d, ^3^ *J*_H‐H_=6.7 Hz, NC(CH_3_)), 1.35 (6H, d, ^3^ *J*_H‐H_=6.7 Hz, NC(C*H*_3_)), 0.90 ppm (9H, s, O^*t*^Bu). ^1^H{^31^P} NMR (500 MHz, *d*_8_‐THF): as above. ^13^C{^1^H} NMR (151 MHz, *d*_8_‐THF): δ=142.3 (d, ^2^ *J*_C‐P_=13.2 Hz, Ar), 128.0 (s, Ar),127.9 (s, Ar), 125.3 (s, Ar), 107.0 (s, Ar), 100.8 (d, ^3^ *J*_C‐P_=2.8 Hz, Ar), 73.0 (d, ^2^ *J*_C‐P_=21.6 Hz, O^*t*^Bu), 71.3 (s, crypt), 68.5 (s, crypt), 54.8 (s, crypt), 46.8 (d, ^2^ *J*_C‐P_=8.3 Hz, NCH), 36.8 (m, *C*(CH_3_)_2_), 36.8 (s, CH_3_), 33.7 (m, CH_3_), 32.1 (d, ^2^ *J*_C‐P_=4.1 Hz, O^*t*^Bu), 23.1 (s, ArCH_3_), 23.0 (d, ^3^ *J*_C‐P_=3.3 Hz, NC(*C*H_3_)), 22.9 ppm (d, ^3^ *J*_C‐P_=6.1 Hz, NC(*C*H_3_)). ^31^P NMR (243 MHz, *d*_8_‐THF): δ=50.2 ppm (s). ^31^P{^1^H} NMR (162 MHz, *d*_8_‐THF): δ=as above. Elemental analysis calcd. (%) for C_45_H_75_K_1_N_5_O_7_P: C 62.26, H 8.71, N 8.07; found: C 61.81, H 8.77, N 7.52.

**Synthesis of 6**: Potassium *tert*‐butoxide (3.12 mg, 27.8 μmol, 1.00 equiv.) and [2.2.2.]‐cryptand (10.5 mg, 27.8 μmol, 1.00 equiv.) is dissolved in diethyl ether (0.5 mL). The resulting solution is added to **4** ^***i*** **Pr**^ (10.5 mg, 27.8 μmol, 1.00 equiv.) in a J. Young NMR tube producing a yellow solution. The solvent is removed *in vacuo*. A solution of methyl triflate (3.0 μL, 27.8 μmol, 1.00 equiv.) in d_6_‐benzene (0.5 mL) is added, producing a colourless solution. The solvent is removed *in vacuo* and the product extracted into pentane (3×0.5 mL). The solvent is removed, then yellow crystals of **6** are grown from a concentrated hexane solution at −30 °C (5.0 mg, 10.7 μmol, 38 %). ^1^H NMR (600 MHz, C_6_D_6_): δ=6.64 (2H, s, Ar), 6.46 (2H, s, Ar), 3.75 (2H, apparent oct, ^3^ *J*_P‐H=_6.7 Hz, ^3^ *J*_H‐H_=6.7 Hz, NCH), 2.43 (6H, s, ArCH_3_), 1.83 (3H, s, CH_3_), 1.81 (3H, s, CH_3_), 1.63 (3H, d, ^2^ *J*_P‐H_=16.6 Hz, PCH_3_), 1.46 (6H, d, ^3^ *J*_H‐H_=6.7 Hz, NC(CH_3_)), 1.35 (6H, d, ^3^ *J*_H‐H_=6.8 Hz, NC(CH_3_)), 1.04 ppm (9H, s, O^*t*^Bu). ^1^H{^31^P} NMR (600 MHz, C_6_D_6_): as above, except δ=3.75 (2H, sept, ^3^ *J*_H‐H_=6.7 Hz, NCH), 1.63 ppm (3H, s, PCH_3_). ^13^C{^1^H} NMR (151 MHz, C_6_D_6_): δ=135.2 (d, ^2^ *J*_C‐P_=8.7 Hz, Ar), 134.1 (d, ^2^ *J*_C‐P_=19.2 Hz, Ar), 131.6 (s, Ar), 128.6 (s, Ar), 118.9 (d, ^2^ *J*_C‐P_=12.7 Hz, Ar), 112.1 (s, Ar), 105.7 (d, ^3^ *J*_C‐P_=4.4 Hz, Ar), 80.0 (d, ^2^ *J*_C‐P_=11.2 Hz, O^*t*^Bu), 45.9 (d, ^2^ *J*_C‐P_=2.6 Hz, NCH), 36.4 (m, *C*(CH_3_)_2_), 35.2 (s, CH_3_), 32.8 (s, CH_3_), 30.5 (d, ^3^ *J*_C‐P_=5.9 Hz, O^*t*^Bu), 25.8 (d, ^1^ *J*_C‐P_=179.4 Hz, PCH_3_), 23.0 (s, ArCH_3_), 20.6 (d, ^3^ *J*_C‐P_=2.2 Hz, NC(*C*H_3_)), 20.5 ppm (s, NC(*C*H_3_)). ^31^P NMR (243 MHz, C_6_D_6_): δ=−39.4 ppm (m). ^31^P{^1^H} NMR (162 MHz, C_6_D_6_): δ=−39.4 ppm (s). Elemental analysis calcd. (%) for C_28_H_42_N_3_OP: C 71.92, H 9.05, N 8.99; found: C 72.20, H 9.46, N 8.47.

**Synthesis of 7**: **4** ^***i*** **Pr**^ (10.5 mg, 27.8 μmol, 1.00 equiv.) and pyridine‐N‐oxide (2.6 mg, 27.8 μmol, 1.00 equiv.) are dissolved in d_6_‐benzene (0.5 mL) in a J. Young NMR tube and stirred at room temperature for 2 h. The solvent is removed *in vacuo* and the residue extracted into pentane (0.5 mL). Colourless crystals of **7** (6.3 mg, 15.9 μmol, 57 %) are grown from a concentrated pentane solution at −30 °C. **Alternative procedure**: **4** ^***i*** **Pr**^ (7.0 mg, 18.5 μmol, 1.00 equiv.) is dissolved in tetrahydrofuran (0.5 mL) in a J. Young NMR tube. The solution is freeze‐pump‐thaw degassed four times then placed under an atmosphere of oxygen (1 atm., excess). The resulting dark red solution is heated at 60 °C for 16 h. The solution is then cooled to room temperature and the solvent removed *in vacuo*. The residues are extracted with pentane (3×1 mL). Off‐white microcrystalline material of **7** (4.0 mg, 10.1 μmol, 55 %) is obtained from a concentrated pentane solution at −30 °C. ^1^H NMR (500 MHz, C_6_D_6_): δ=6.56 (2H, s, Ar), 6.32 (2H, s, Ar), 3.98 (2H, d‐sept, ^3^ *J*_P‐H_=15.4 Hz, ^3^ *J*_H‐H_=6.9 Hz, NCH), 2.19 (6H, s, ArCH_3_), 1.59 (3H, s, CH_3_), 1.55 (3H, s, CH_3_), 1.48 (6H, d, ^3^ *J*_H‐H_=6.8 Hz, NC(CH_3_)), 1.35 ppm (6H, d, ^3^ *J*_H‐H_=6.8 Hz, NC(CH_3_)). ^1^H{^31^P} NMR (500 MHz, C_6_D_6_): as above, except δ=3.98 ppm (2H, sept, ^3^ *J*_H‐H_=6.9 Hz, NCH). ^13^C{^1^H} NMR (151 MHz, C_6_D_6_): δ=135.3 (s, Ar), 135.2 (d, *J*_C‐P_=9.68 Hz, Ar), 133.9 (s, Ar), 133.2 (d, *J*_C‐P_=8.8 Hz, Ar), 115.8 (s, Ar), 109.9 (d, ^3^ *J*_C‐P_=10.5 Hz, Ar), 46.6 (d, ^2^ *J*_C‐P_=3.5 Hz, NCH), 40.2 (m, *C*(CH_3_)_2_), 26.4 (s, CH_3_), 22.6 (s, CH_3_), 22.3 (s, ArCH_3_), 21.4 (d, ^3^ *J*_C‐P_=1.1 Hz, NC(*C*H_3_)), 20.8 ppm (d, ^3^ *J*_C‐P_=2.0 Hz, NC(*C*H_3_). ^31^P NMR (202 MHz, C_6_D_6_): δ=37.4 ppm (t, ^3^ *J*_P‐H_=15.4 Hz). ^31^P{^1^H} NMR (162 MHz, C_6_D_6_): δ=37.4 ppm (s). Elemental analysis calcd. (%) for C_23_H_30_N_3_O_1_P: C 69.85, H 7.65, N 10.63; found: C 69.19, H 7.61, N 10.09.

**Synthesis of 8**: **7** (6.0 mg, 15.2 μmol, 1.00 equiv.) and 2,4‐bis(4‐methoxyphenyl‐1,3,2,4‐dithiadiphosphetane‐2,4‐disulfide (3.1 mg, 7.6 μmol, 0.50 equiv.) are dissolved in toluene (0.5 mL) in a J. Young NMR tube and heated to 110 °C for 16 h. Full conversion to **8** is observed by ^31^P NMR spectroscopy. The solvent is removed *in vacuo* and the product extracted into hexane (0.5 mL). **8** was crystallised at −30 °C following concentration of this hexane solution by slow evaporation (6.0 mg, 14.6 μmol, 96 %). **Alternative procedure**: **4** ^***i*** **Pr**^ (15.0 mg, 39.5 μmol, 1.00 equiv.) and S_8_ (6.4 mg, 0.198 mmol, 5.00 equiv.) are dissolved in *d*_2_‐dichloromethane (0.5 mL) in a J. Young NMR tube and stirred for 4 h. Full conversion to thiophosphorane **8** is observed by ^31^P{^1^H} and ^1^H NMR, however isolation of this product is hindered by co‐crystallisation of sulfur. ^1^H NMR (500 MHz, C_6_D_6_): δ=6.55 (2H, s, Ar), 6.34 (2H, s, Ar), 4.26 (2H, d‐sept, ^3^ *J*_P‐H_=15.0 Hz, ^3^ *J*_H‐H_=7.1 Hz, NCH), 2.17 (6H, s, ArCH_3_), 1.58 (3H, s, CH_3_), 1.56 (3H, s, CH_3_), 1.51 (6H, d, ^3^ *J*_H‐H_=7.0 Hz, NC(CH_3_)), 1.29 ppm (6H, d, ^3^ *J*_H‐H_=7.0 Hz, NC(CH_3_)). ^1^H{^31^P} NMR (500 MHz, C_6_D_6_): as above, except δ=4.26 ppm (2H, sept, ^3^ *J*_H‐H_=7.0 Hz, NCH). ^13^C{^1^H} NMR (151 MHz, C_6_D_6_): δ=135.4 (d, ^3^ *J*_C‐P_=10.6 Hz, Ar), 134.5 (s, Ar), 134.4 (d, *J*_C‐P_=12.6 Hz, Ar), 133.8 (s, Ar), 116.1 (s, Ar) 109.9 (s, Ar), 48.4 (d, ^2^ *J*_C‐P_=5.9 Hz, NCH), 40.2 (s, *C*(CH_3_)_2_), 26.2 (s, CH_3_), 22.7 (s, CH_3_), 22.2 (s, ArCH_3_)), 20.8 (d, ^3^ *J*_C‐P_=1.6 Hz, NC(*C*H_3_)), 20.1 ppm (d, ^3^ *J*_C‐P_=3.5 Hz, NC(*C*H_3_)). ^31^P NMR (202 MHz, C_6_D_6_): δ=83.8 ppm (t, ^3^ *J*_P‐H_=15.0 Hz). ^31^P{^1^H} NMR (162 MHz, C_6_D_6_): δ=83.8 ppm (s). HRMS (ESI) *m/z* calcd. for C_23_H_30_N_3_PS+H^+^: 412.1971 [*M*+H]^+^; found: 412.1964.

**Synthesis of 9**: **4** ^***i*** **Pr**^ (10.0 mg, 26.4 μmol, 1.00 equiv.) and selenium powder (10.4 mg, 0.132 mmol, 5.00 equiv.) are suspended in d_2_‐dichloromethane (0.5 mL) in a J. Young NMR tube and stirred for 2.5 h at room temperature. Full conversion to **9** was observed by ^31^P NMR spectroscopy. The solvent is removed *in vacuo* and the residue is washed with hexane (0.5 mL). The product is extracted into tetrahydrofuran (0.3 mL), filtered, and crystallised by pentane vapour diffusion (2 mL). **9** is isolated as yellow crystals after one week (6.5 mg, 14.2 μmol, 54 %). ^1^H NMR (500 MHz, C_6_D_6_): δ=6.55 (2H, s, Ar), 6.36 (2H, s, Ar), 4.43 (2H, d‐sept, ^3^ *J*_P‐H_=14.6 Hz, ^3^ *J*_H‐H_=6.9 Hz, NCH), 2.16 (6H, s, ArCH_3_), 1.58 (3H, s, CH_3_), 1.55 (3H, s, CH_3_), 1.52 (6H, d, ^3^ *J*_H‐H_=6.9 Hz, NC(CH_3_)), 1.27 ppm (6H, d, ^3^ *J*_H‐H_=7.0 Hz, NC(CH_3_)). ^1^H{^31^P} NMR (500 MHz, C_6_D_6_): as above, except δ=4.43 ppm (2H, sept, ^2^ *J*_H‐H_=7.0 Hz, NCH). ^13^C{^1^H} NMR (151 MHz, C_6_D_6_): δ=135.4 (d, ^3^ *J*_C‐P_=10.4 Hz, Ar), 135.0 (d, ^2^ *J*_C‐P_=3.0 Hz, Ar), 134.0 (d, ^2^ *J*_C‐P_=14.9 Hz, Ar), 133.8 (s, Ar), 116.2 (s, Ar), 110.1 (d, ^3^ *J*_C‐P_=8.6 Hz, Ar), 47.1 (d, ^2^ *J*_C‐P_=7.4 Hz, NCH), 40.2 (s, *C*(CH_3_)_2_), 26.3 (s, CH_3_), 22.6 (s, CH_3_), 22.2 (s, ArCH_3_), 20.7 (d, ^3^ *J*_C‐P_=1.7 Hz, NC(*C*H_3_)), 20.0 ppm (d, ^3^ *J*_C‐P_=3.9 Hz, NC(*C*H_3_)). ^31^P NMR (243 MHz, C_6_D_6_): δ=75.7 ppm (dt, ^1^ *J*_P‐77Se_=919.2 Hz, ^3^ *J*_P‐H_=14.7 Hz). ^31^P{^1^H} NMR (162 MHz, C_6_D_6_): δ=75.6 ppm (dt, ^1^ *J*_P‐77Se_=918.8 Hz). ^77^Se NMR (95 MHz, C_6_D_6_): δ=−35.4 ppm (d, ^1^ *J*_P‐77Se_=916.8 Hz). Elemental analysis calcd. (%) for C_23_H_30_N_3_PSe: C 60.26, H 6.60, N 9.17; found: C 60.05, H 6.61, N 9.05.

**Synthesis of 10**: **4** ^***i*** **Pr**^ (10.5 mg, 27.8 μmol, 1.00 equiv.) is dissolved in a toluene (0.5 mL) solution of mesityl‐azide (4.5 mg, 27.8 μmol, 1.00 equiv.) in a J. Young NMR tube. The solution was heated at 110 °C for 2 h generating a colourless solution. The solvent is removed *in vacuo* and the residues heated under dynamic vacuum for 30 min to remove any unreacted mesityl‐azide. The residue is dissolved in hexane (0.5 mL) and crystals of **10** (10.0 mg, 19.5 μmol, 70 %) were grown by slow evaporation of this hexane solution. ^1^H NMR (500 MHz, C_6_D_6_): δ=6.92 (2H, s, Ar^Mes^), 6.61 (2H, s, Ar), 6.44 (2H, s, Ar), 4.13 (2H, d‐sept, ^3^ *J*_P‐H_=11.7 Hz, ^3^ *J*_H‐H_=7.0 Hz, NCH), 2.50 (6H, br s, CH_3_^Mes^), 2.26 (3H, d, *J*_P‐H_=2.8 Hz, CH_3_^Mes^), 2.21 (6H, s, ArCH_3_), 1.66 (3H, s, CH_3_), 1.64 (3H, s, CH_3_), 1.30 (6H, d, ^3^ *J*_H‐H_=6.9 Hz, NC(CH_3_)), 1.17 ppm (6H, d, ^3^ *J*_H‐H_=7.0 Hz, NC(CH_3_)). ^1^H{^31^P} NMR (500 MHz, C_6_D_6_): as above, except δ=4.13 (2H, sept, ^3^ *J*_H‐H_=7.0 Hz, NCH), 2.26 ppm (3H, s, CH_3_^Mes^). ^13^C{^1^H} NMR (151 MHz, C_6_D_6_): δ=143.1 (d, *J*_C‐P_=3.4 Hz, Ar^Mes^), 134.4 (d, ^3^ *J*_C‐P_=10.0 Hz, Ar), 133.8 (d, ^2^ *J*_C‐P_=19.6 Hz, Ar), 133.5 (d, ^2^ *J*_C‐P_=7.3 Hz, Ar), 133.5 (s, Ar), 131.3 (m, Ar^Mes^), 129.5 (m, Ar^Mes^), 129.0 (d, *J*_C‐P_=4.1 Hz, Ar^Mes^), 115.6 (s, Ar), 110.3 (d, ^3^ *J*_C‐P_=10.4 Hz, Ar), 47.0 (d, ^2^ *J*_C‐P_=2.6 Hz, NCH), 40.0 (s, *C*(CH_3_)_2_), 26.8 (s, CH_3_), 22.5 (s, CH_3_), 22.3 (s, ArCH_3_), 20.95–20.88 (overlapping signals corresponding to one CH_3_^Mes^ environment, and one set of NC(*C*H_3_) environments), 20.7 (s, CH_3_^Mes^), 20.0 ppm (d, ^3^ *J*_C‐P_=4.4 Hz, NC(*C*H_3_)). ^31^P NMR (243 MHz, C_6_D_6_): δ=18.1 ppm (m). ^31^P{^1^H} NMR (243 MHz, C_6_D_6_): δ=18.1 ppm (s). Elemental analysis calcd (%) for C_32_H_41_N_4_P: C 74.97, H 8.06, N 10.93; found: C 74.91, H 8.30, N 10.54.

**Synthesis of 11**: **4** ^***i*** **Pr**^ (10.0 mg, 26.4 μmol, 1.00 equiv.) is dissolved in d_6_‐benzene (0.5 mL) in a J. Young NMR tube. Methyl triflate (2.9 μL, 26.4 μmol, 1.0 equiv.) is added and the solution heated to 80 °C for 3 h. Yellow needle‐like crystals form over the course of the reaction. The mixture is cooled gradually to room temperature and left for 16 h. The crystals are filtered, washed with benzene (3×0.5 mL) and dried *in vacuo* to yield **11** (7.5 mg, 13.8 μmol, 52 %). ^1^H NMR (500 MHz, CD_2_Cl_2_): δ=6.88 (2H, s, Ar), 6.81 (2H, s, Ar), 4.55 (2H, d‐sept, ^3^ *J*_P‐H_=11.6 Hz, ^3^ *J*_H‐H_=6.9 Hz, NCH), 3.13 (3H, d, ^2^ *J*_P‐H_=15.6 Hz, PCH_3_), 2.38 (6H, s, ArCH_3_), 1.82 (3H, s, CH_3_), 1.70 (6H, d, ^3^ *J*_H‐H_=7.0 Hz, NC(CH_3_)), 1.64 (3H, s, CH_3_), 1.58 ppm (6H, d, ^3^ *J*_H‐H_=6.8 Hz, NC(CH_3_)). ^1^H{^31^P} NMR (500 MHz, CD_2_Cl_2_): as above, except δ=4.55 (2H, sept, ^3^ *J*_H‐H_=6.8 Hz, NCH), 3.13 ppm (3H, s, PCH_3_). ^13^C{^1^H} NMR (151 MHz, CD_2_Cl_2_): δ=137.0 (s, Ar), 136.4 (d, ^3^ *J*_C‐P_=10.4 Hz, Ar), 133.0 (d, ^4^ *J*_C‐P_=3.0 Hz, Ar), 131.4 (d, ^2^ *J*_C‐P_=15.7 Hz, Ar), 119.4 (d, ^4^ *J*_C‐P_=1.8 Hz, Ar), 113.0 (d, ^3^ *J*_C‐P_=9.1 Hz, Ar), 47.9 (d, ^2^ *J*_C‐P_=4.1 Hz, NCH), 40.5 (s, *C*(CH_3_)), 26.9 (s, CH_3_), 22.5 (s, CH_3_), 22.2 (s, ArCH_3_), 21.6 (s, NC(*C*H_3_)), 21.1 (d, ^3^ *J*_C‐P_=5.8 Hz, NC(*C*H_3_)), 16.0 ppm (d, ^1^ *J*_C‐P_=125.1 Hz, PCH_3_). ^31^P NMR (243 MHz, CD_2_Cl_2_): δ=80.5 ppm (qt, ^2^ *J*_P‐H_=15.4 Hz, ^3^ *J*_P‐H_=11.6 Hz). ^31^P{^1^H} NMR (243 MHz, CD_2_Cl_2_): δ=80.5 ppm (s). ^19^F{^1^H} NMR (470 MHz, CD_2_Cl_2_): δ=78.9 ppm (s, OTf). Elemental analysis calcd. (%) for C_25_H_33_F_3_N_3_O_3_PS: C 55.24, H 6.12, N 7.73; found: C 55.81, H 6.07, N 7.59.

**Synthesis of 12**: **4** ^***i*** **Pr**^ (10.5 mg, 27.8 μmol, 1.00 equiv.) is dissolved in d_6_‐benzene (0.5 mL) in a J. Young NMR tube. Methyl triflate (3.0 μL, 27.8 μmol, 1.00 equiv.) is added and the solution heated to 80 °C for 3 h. The solvent is removed *in vacuo* and the residues dissolved in a solution of potassium *tert*‐butoxide (3.1 mg, 27.8 μmol, 1.00 equiv.) in tetrahydrofuran (0.5 mL). A colourless solution results. The solvent is removed *in vacuo* and the product extracted into hexane (2×0.5 mL). Colourless crystals of **12** (7.6 mg, 19.3 μmol, 69 %) were grown from a concentrated hexane solution at −30 °C. ^1^H NMR (600 MHz, C_6_D_6_): δ=6.63 (2H, s, Ar), 6.47 (2H, s, Ar), 4.23 (2H, d‐sept, ^3^ *J*_P‐H_=11.4 Hz, ^3^ *J*_H‐H_=7.0 Hz, NCH), 2.26 (6H, s, ArCH_3_), 1.65 (3H, s, CH_3_), 1.59 (3H, s, CH_3_), 1.40 (6H, d, ^3^ *J*_H‐H_=7.0 Hz, NC(CH_3_)), 1.20 (2H, d, ^2^ *J*_P‐H_=18.5 Hz, PCH_2_), 1.19 ppm (6H, d, ^3^ *J*_H‐H_=7.1 Hz, NC(CH_3_)). ^1^H{^31^P} NMR (600 MHz, C_6_D_6_): as above, except δ=4.23 (2H, sept, ^3^ *J*_H‐H_=7.0 Hz, NCH), 1.20 ppm (2H, s, PCH_2_). ^13^C{^1^H} NMR (151 MHz, C_6_D_6_): δ=133.8 (d, ^2^ *J*_C‐P_=4.1 Hz, Ar), 133.1 (d, ^2^ *J*_C‐P_=16.4 Hz, Ar), 132.9 (d, ^3^ *J*_C‐P_=10.3 Hz, Ar), 132.4 (s, Ar), 115.6 (s, Ar), 109.8 (d, ^3^ *J*_C‐P_=8.9 Hz, Ar), 47.1 (d, ^2^ *J*_C‐P_=3.9 Hz, NCH), 39.4 (s, *C*(CH_3_)_2_), 27.4 (s, CH_3_), 22.8 (s, CH_3_), 22.4 (s, CH_3_), 20.9 (s, NC(*C*H_3_)), 20.5 (d, ^3^ *J*_C‐P_=5.2 Hz, NC(*C*H_3_)), 16.9 ppm (d, ^1^ *J*_C‐P_=240.1 Hz, PCH_2_). ^31^P NMR (243 MHz, C_6_D_6_): δ=61.2 ppm (m). ^31^P{^1^H} NMR (162 MHz, C_6_D_6_): δ=61.2 ppm (s). Elemental analysis calcd. (%) for C_24_H_32_N_3_P: C 73.25, H 8.20, N 10.68; found: C 73.63, H 8.40, N 10.16.

**Synthesis of 13**: **4** ^***i*** **Pr**^ (10.0 mg, 26.4 μmol, 1.00 equiv.) is dissolved in d_6_‐benzene (0.5 mL) in a J. Young NMR tube. Catachole borane (2.8 μL, 26.4 μmol, 1.00 equiv.) is added, resulting in the immediate generation of a colourless solution. Full conversion to an isomeric mixture of **13** (1:0.6 ratio) was observed by ^31^P NMR spectroscopy. The solvent is removed *in vacuo*, dissolved in benzene and lyophilised to yield the isomeric mixture of **13** as an off white powder (9.6 mg, 19.2 μmol, 69 %). ^1^H NMR (600 MHz, C_6_D_6_): ***major isomer*** δ=7.20 (1H, s, Ar), 7.02 (1H, d, ^1^ *J*_P‐H_=135.0 Hz, P−H), 6.91 (Ar^BCat^), 6.86 (1H, m, Ar), 6.70 (1H, s, Ar), 6.68 (Ar^BCat^), 6.27 (1H, s, Ar), 4.35 (1H, sept, ^2^ *J*_H‐H_=6.7 Hz, NCH), 3.54 (1H, d‐sept, ^3^ *J*_P‐H_=11.6 Hz, ^3^ *J*_H‐H_=6.6 Hz, NCH), 2.30 (3H, s, ArCH_3_), 2.18 (3H, s, ArCH_3_), 1.71 (3H, s, CH_3_), 1.51 (3H, s, CH_3_), 1.43 (3H, d, ^2^ *J*_H‐H_=6.6 Hz, NC(CH_3_)), 1.18 (3H, d, ^2^ *J*_H‐H_=6.8 Hz, NC(CH_3_)), 1.18 (3H, d, ^2^ *J*_H‐H_=6.6 Hz, NC(CH_3_)), 0.94 ppm (3H, d, ^2^ *J*_H‐H_=6.5 Hz, NC(CH_3_)). ***minor isomer*** δ=7.16 (Ar), 7.15 (1H, d, ^1^ *J*_P‐H_=148.0 Hz, P−H), 6.98 (2H, br m, Ar^BCat^), 6.91 (Ar), 6.74 (2H, dd, ^3^ *J*_*H*‐H_=5.7 Hz, ^4^ *J*_*H*‐H_=3.3 Hz, Ar^BCat^), 6.66 (1H, s, Ar), 6.25 (s, 1H, Ar), 4.11 (1H, sept, ^2^ *J*_H‐H_=6.7 Hz, NCH), 3.44 (1H, d‐sept, ^3^ *J*_P‐H_=12.7 Hz, ^2^ *J*_H‐H_=6.3 Hz, NCH), 2.29 (3H, s, ArCH_3_), 2.18 (3H, s, ArCH_3_), 1.64 (3H, s, CH_3_), 1.59 (3H, s, CH_3_), 1.38 (3H, d, ^2^ *J*_H‐H_=6.5 Hz, NC(CH_3_)), 1.00 (3H, d, ^2^ *J*_H‐H_=6.6 Hz, NC(CH_3_)), 0.90 (3H, d, ^2^ *J*_H‐H_=6.5 Hz, NC(CH_3_)), 0.86 ppm (3H, d, ^2^ *J*_H‐H_=6.6 Hz, NC(CH_3_)). ^1^H{^31^P} NMR (500 MHz, C_6_D_6_): ***major isomer*** as above, except δ=7.02 (1H, s, P−H), 3.54 ppm (1H, sept, ^2^ *J*_H_‐_H_=6.6 Hz, NCH). ***minor isomer*** as above, except δ=7.15 (1H, s, P−H), 3.44 ppm (1H, sept, ^2^ *J*_H‐H_=6.3 Hz, NCH). ^13^C{^1^H} NMR (151 MHz, C_6_D_6_): ***major isomer*** δ=149.4 (s, Ar^BCat^), 139.6 (d, ^2^ *J*_C‐P_=6.7 Hz, Ar), 135.5 (d, ^2^ *J*_C‐P_=3.6 Hz, Ar), 134.5 (d, ^3^ *J*_C‐P_=2.1 Hz, Ar), 133.3 (d, ^2^ *J*_C‐P_=9.5 Hz, Ar), 131.3 (s, Ar), 130.9 (s, Ar), 130.0 (s, Ar), 128.4 (s, Ar), 127.0 (s, Ar), 122.3 (s, Ar^BCat^), 116.4 (s, Ar), 112.1 (s, Ar^BCat^), 108.5 (s, Ar), 51.9 (d, ^5TS^ *J*_C‐P_=13.4 Hz, NCH), 47.5 (d, ^2^ *J*_C‐P_=17.4 Hz, NCH), 36.6 (s, *C*(CH_3_)_2_), 34.8 (s, CH_3_), 31.5 (s, CH_3_), 24.3 (s, NC(*C*H_3_)), 22.2 (d, ^3^ *J*_C‐P_=10.9 Hz, NC(*C*H_3_)), 21.9 (s, ArCH_3_), 21.4 (s, NC(*C*H_3_)), 20.9 (s, ArCH_3_), 19.0 ppm (d, ^6TS^ *J*_C‐P_=6.1 Hz, NC(*C*H_3_)). ***minor isomer*** δ=149.7 (s, Ar^BCat^), 139.5 (d, ^2^ *J*_C‐P_=6.5 Hz, Ar), 134.4 (s, Ar), 133.8 (d, ^3^ *J*_C_‐_P_=2.0 Hz, Ar), 132.8 (d, ^2^ *J*_C‐P_=7.8 Hz, Ar), 131.2 (s, Ar), 129.3 (s, Ar), 128.3 (s, Ar), 126.8 (s, Ar), 126.7 (s, Ar), 122.0 (s, Ar^BCat^),116.8 (s, Ar), 112.0 (s, Ar^BCat^), 108.8 (s, Ar), 50.5 (s, NCH), 47.9 (d, ^2^ *J*_C‐P_=18.1 Hz, NCH), 36.7 (s, *C*(CH_3_)_2_), 34.6 (s, CH_3_), 33.0 (s, CH_3_), 25.1 (s, NC(*C*H_3_)), 21.9 (s, ArCH_3_), 21.6 (d, ^2^ *J*_C‐P_=12.2 Hz, NC(*C*H_3_)), 21.3 (s, NC(*C*H_3_)), 20.9 (s, ArCH_3_), 20.6 ppm (s, NC(*C*H_3_)). ^31^P NMR (243 MHz, C_6_D_6_): ***major isomer*** δ=67.1 ppm (dd, ^1^ *J*_P‐H_=135.0 Hz, ^3^ *J*_P‐H_=11.3 Hz). ***minor isomer*** δ=61.8 ppm (dd, ^1^ *J*_P‐H_=148.4 Hz, ^3^ *J*_P‐H_=12.3 Hz). ^31^P{^1^H} NMR (202 MHz, C_6_D_6_): ***major isomer*** δ=67.1 ppm (s). ***minor isomer*** δ=61.8 ppm (s). ^11^B{^1^H} NMR (128 MHz, C_6_D_6_): δ=−28.6 (br), −34.0 ppm (br) (assignment of major and minor isomer could not be achieved).

**Synthesis of 14**: **4** ^***i*** **Pr**^ (10.5 mg, 27.8 μmol, 1.00 equiv.) is dissolved in d_6_‐benzene (0.5 mL) in a J. Young NMR tube. Hydrochloric acid (0.2 M in diethyl ether, 0.14 mL, 27.8 μmol, 1.00 equiv.) is added, instantly generating a colourless solution. The solvent was removed *in vacuo* and the product dissolved in pentane (0.5 mL). Pale yellow crystals of **14** (8.5 mg, 20.4 μmol, 74 %) were grown from a concentrated pentane solution at −30 °C. ^1^H NMR (500 MHz, C_6_D_6_): δ=7.06 (1H, s, Ar), 6.83 (1H, s, Ar), 6.58 (1H, s, Ar), 6.55 (1H, s, Ar), 3.99 (1H, d‐sept, ^3^ *J*_P‐H_=11.5 Hz, ^3^ *J*_H‐H_=6.7 Hz, NCH), 3.19 (1H, sept, ^3^ *J*_H‐H_=6.2 Hz, NCH), 2.97 (1H, br s, N−H), 2.33 (3H, s, ArCH_*3*_), 2.19 (3H, s, ArCH_3_), 1.57 (6H, br s, C*H*_3_), 1.54 (6H, d, ^3^ *J*_H‐H_=6.6 Hz, NC(CH_3_)), 1.10 ppm (6H, d, ^3^ *J*_H‐H_=6.2 Hz, NC(CH_3_)). ^1^H{^31^P} NMR (500 MHz, C_6_D_6_): as above, except δ=3.99 ppm (1H, sept, ^3^ *J*_H‐H_=6.7 Hz, NCH). ^13^C{^1^H} NMR (151 MHz, C_6_D_6_): δ=134.9 (d, ^2^ *J*_C‐P_=9.8 Hz, Ar), 134.7 (d, ^2^ *J*_C‐P_=3.4 Hz, Ar), 133.6 (s, Ar), 132.4 (s, Ar), 132.3 (s, Ar), 131.2 (d, ^2^ *J*_C‐P_=8.0 Hz, Ar), 130.1 (s, Ar), 129.1 (d, ^2^ *J*_C‐P_=12.1 Hz, Ar), 125.2 (s, Ar), 125.0 (s, Ar), 117.9 (s, Ar), 109.1 (s, Ar), 49.0 (d, ^5TS^ *J*_C‐P_=6.0 Hz, NCH), 47.5 (d, ^2^ *J*_C‐P_=15.8 Hz, NCH), 36.4 (s, *C*(CH_3_)_2_), 22.0 (s, ArCH_3_), 21.1 ppm (s, ArCH_3_). ^31^P NMR (243 MHz, C_6_D_6_): δ=130.6 ppm (m). ^31^P{^1^H} NMR (243 MHz, C_6_D_6_): δ=130.6 ppm (s). Elemental analysis calcd. (%) for C_23_H_31_ClN_3_P: C 66.42, H 7.51, N 10.10; found: C 67.21, H 8.24, N 9.01. This is consistent with the presence of 0.5 equiv. pentane in the crystalline sample submitted.

**Synthesis of 15**: **4** ^***i*** **Pr**^ (31.0 mg, 81.7 μmol, 1.00 equiv.) is dissolved in d_6_‐benzene (0.5 mL) in a J. Young NMR tube. Water (1.5 μL, 81.7 μmol, 1.00 equiv.) is added resulting in an immediate colour change to colourless. The solvent is removed *in vacuo* and the remaining residue heated under vacuum to remove traces of water. Lyophilisation from a concentrated benzene solution yields **15** as a colourless powder (25.5 mg, 64.2 μmol, 79 %). ^1^H NMR (400 MHz, C_6_D_6_): δ=9.42 (1H, d, ^1^ *J*_P‐H_=714.6 Hz, P‐*H*), 6.97 (1H, s, Ar), 6.69 (1H, s, Ar), 6.64 (1H, s, Ar), 6.29 (1H, s, Ar), 3.85 (1H, s, N−H), 3.72 (1H, d‐sept, ^3^ *J*_P‐H_=18.0 Hz, ^3^ *J*_H‐H_=6.7 Hz, NCH), 3.31 (1H, sept, ^3^ *J*_H‐H_=6.3 Hz, NCH), 2.31 (3H, s, ArCH_3_), 2.18 (3H, s, ArCH_3_), 1.64 (3H, s, CH_3_), 1.54 (3H, s, CH_3_), 1.41 (3H, d, ^3^ *J*_H‐H_=6.7 Hz, NC(CH_3_)), 1.40 (3H, d, ^3^ *J*_H‐H_=6.7 Hz, NC(CH_3_)), 1.21 (3H, d, ^3^ *J*_H‐H_=6.3 Hz, NC(CH_3_)), 0.95 ppm (3H, d, ^3^ *J*_H‐H_=6.3 Hz, NC(CH_3_)). ^1^H{^31^P} NMR (400 MHz, C_6_D_6_): as above, except δ=9.42 (1H, s, P−H), 3.72 ppm (1H, sept, ^3^ *J*_H‐H_=6.7 Hz, NCH). ^13^C{^1^H} NMR (151 MHz, C_6_D_6_): δ=133.6 (s, Ar), 133.0 (d, ^2^ *J*_C‐P_=8.6 Hz, Ar), 132.9 (d, ^2^ *J*_C‐P_=5.7 Hz, Ar), 131.9 (s, Ar), 131.5 (s, Ar), 129.9 (s, Ar), 127.3 (d, ^3^ *J*_C‐P_=5.9 Hz, Ar), 125.1 (d, ^2^ *J*_C‐P_=10.8 Hz, Ar), 123.9 (s, Ar), 121.8 (s, Ar), 116.3 (s, Ar), 106.6 (d, ^3^ *J*_C‐P_=6.16 Hz, Ar), 47.0 (s, NCH), 46.3 (d, ^2^ *J*_C‐P_=5.0 Hz, NCH), 36.2 (s, *C*(CH_3_)_2_), 34.5 (s, CH_3_), 34.0 (s, CH_3_), 22.6 (s, NC(*C*H_3_)), 22.1 (s, ArCH_3_), 21.8 (s, NC(*C*H_3_)), 21.2 (s, ArCH_3_), 21.0 (s, NC(*C*H_3_)), 20.0 ppm (d, ^3^ *J*_C‐P_=3.73 Hz, NC(*C*H_3_)). ^31^P NMR (162 MHz, C_6_D_6_): δ=1.3 ppm (dd, ^1^ *J*_P‐H_=714.6 Hz, ^3^ *J*_P‐H_=18.3 Hz). ^31^P{^1^H} NMR (162 MHz, C_6_D_6_): δ=1.3 ppm (s). Elemental analysis calcd. (%) for C_23_H_32_N_3_OP: C 69.50, H 8.11, N 10.57; found: C 68.72, H 7.68, N 9.97. This is consistent with the presence of 0.25 equiv. water in the crystalline sample submitted.

**Synthesis of 16**: **4** ^***i*** **Pr**^ (10.5 mg, 27.8 μmol, 1.00 equiv.) is dissolved in d_6_‐benzene (0.5 mL) in a J. Young NMR tube. Methanol (2 M in DME, 14.0 μL, 27.8 μmol, 1.00 equiv.) is added resulting in the generation of a pale‐yellow solution. The solvent is removed *in vacuo* and the residue extracted with pentane (2×0.5 mL). Crystals of **16** (8.5 mg, 20.7 μmol, 74 %) are grown from a concentrated pentane solution at −30 °C. ^1^H NMR (600 MHz, C_6_D_6_): δ=7.02 (1H, s, Ar), 6.79 (1H, s, Ar), 6.57 (1H, s, Ar), 6.43 (1H, s, Ar), 3.92 (1H, d‐sept, ^3^ *J*_P‐H_=10.2 Hz, ^3^ *J*_H‐H_=6.7 Hz, NCH), 3.47 ‐ 3.39 (2H, N−H and NCH), 2.81 (3H, d, ^3^ *J*_P‐H_=7.4 Hz, OCH_3_), 2.39 (3H, s, ArCH_3_), 2.28 (3H, s, ArC*H*_3_), 1.76 (3H, s, CH_3_), 1.72 (3H, s, CH_3_), 1.43 (3H, dd, ^3^ *J*_H‐H_=6.7 Hz, ^4^ *J*_P‐H_=1.1 Hz, NC(CH_3_)), 1.38 (3H, d, ^3^ *J*_H‐H_=6.7 Hz, NC(CH_3_)), 1.19 ‐ 1.17 ppm (6H, NCH(C*H*_3_). ^1^H{^31^P} NMR (600 MHz, C_6_D_6_): as above, except δ=3.92 (1H, sept, ^3^ *J*_H‐H_=6.7 Hz, NCH), 2.81 (3H, s, OCH_3_), 1.43 ppm (3H, d, ^3^ *J*_H‐H_=6.7 Hz, NC(CH_3_)). ^13^C{^1^H} NMR (151 MHz, C_6_D_6_): δ=136.4 (d, ^2^ *J*_C‐P_=7.8 Hz, Ar), 134.4 (d, ^3^ *J*_C‐P_=1.5 Hz, Ar), 133.4 (d, ^3^ *J*_C‐P_=2.1 Hz, Ar), 130.8 (s, Ar), 130.7 (s, Ar), 130.1 (d, ^2^ *J*_C‐P_=8.8 Hz, Ar), 128.6 (s, Ar), 127.0 (s, Ar), 121.3 (s, Ar), 119.0 (s, Ar), 116.2 (s, Ar), 106.1 (s, Ar), 49.7 (d, ^2^ *J*_C‐P_=5.4 Hz, OCH_3_), 46.7 (d, ^2^ *J*_C‐P_=19.5 Hz, NCH), 46.5 (d, ^5TS^ *J*_C‐P_=5.9 Hz, NCH), 36.6 (s, *C*(CH_3_)_2_), 34.7 (s, CH_3_), 34.6 (s, CH_3_), 22.7 ‐ 22.6 (overlapping multiplets, NC(*C*H_3_)), 22.4 (s, NC(*C*H_3_)), 22.1 (s, ArCH_3_), 21.3 ppm (s, ArCH_3_). ^31^P NMR (243 MHz, C_6_D_6_): δ=97.3 ppm (m). ^31^P{^1^H} NMR (243 MHz, C_6_D_6_): δ=97.3 ppm (s). Elemental analysis calcd. (%) for C_24_H_34_N_3_OP: C 70.05, H 8.33, N 10.21; found: C 70.15, H 8.44, N 9.80.

## Conflict of interest

The authors declare no conflict of interest.

1

## Supporting information

As a service to our authors and readers, this journal provides supporting information supplied by the authors. Such materials are peer reviewed and may be re‐organized for online delivery, but are not copy‐edited or typeset. Technical support issues arising from supporting information (other than missing files) should be addressed to the authors.

Supporting Information

## Data Availability

The data that support the findings of this study are available in the supplementary material of this article.

## References

[1] P. P. Power , Nature 2010, 463, 171–177. 20075912 10.1038/nature08634

[2] T. Chu , G. I. Nikonov , Chem. Rev. 2018, 118, 3608–3680. 29558125 10.1021/acs.chemrev.7b00572

[3] C. Weetman , S. Inoue , ChemCatChem 2018, 10, 4213–4228.

[4] R. L. Melen , Science 2019, 363, 479–484. 30705183 10.1126/science.aau5105

[5] C. Weetman , Chem. Eur. J. 2021, 27, 1941–1954. 32757381 10.1002/chem.202002939 PMC7894548

[6] J. M. Lipshultz , G. Li , A. T. Radosevich , J. Am. Chem. Soc. 2021, 143, 1699–1721. 33464903 10.1021/jacs.0c12816 PMC7934640

[7] J. Abbenseth , J. M. Goicoechea , Chem. Sci. 2020, 11, 9728–9740. 34094237 10.1039/d0sc03819a PMC8162179

[8] S. Kundu , Chem. Asian J. 2020, 15, 3209–3224. 32794320 10.1002/asia.202000800

[9] A. Brand , W. Uhl , Chem. Eur. J. 2019, 25, 1391–1404. 30126018 10.1002/chem.201803331

[10] L. Greb , F. Ebner , Y. Ginzburg , L. M. Sigmund , Eur. J. Inorg. Chem. 2020, 3030–3047.

[11] L. Wang , Y. Li , Z. Li , M. Kira , Coord. Chem. Rev. 2022, 457, 214413.

[12] Y. Mizuhata , T. Sasamori , N. Tokitoh , Chem. Rev. 2009, 109, 3479–3511. 19630390 10.1021/cr900093s

[13] D. Bourissou , O. Guerret , F. P. Gabbaï , G. Bertrand , Chem. Rev. 2000, 100, 39–92. 11749234 10.1021/cr940472u

[14] M. S. Nechaev , Organometallics 2021, 40, 3408–3423.

[15] C. Bonningue , D. Houalla , M. Sanchez , R. Wolf , F. H. Osman , J. Chem. Soc. Perkin Trans. 2 1981, 19–25.

[16] C. Bonningue , D. Houalla , R. Wolf , J. Jaud , J. Chem. Soc. Perkin Trans. 2 1983, 773–776.

[17] D. Houalla , F. H. Osman , M. Sanchez , R. Wolf , Tetrahedron Lett. 1977, 18, 3041–3044.

[18] S. A. Culley , A. J. Arduengo , J. Am. Chem. Soc. 1984, 106, 1164–1165.

[19] T. P. Robinson , D. M. De Rosa , S. Aldridge , J. M. Goicoechea , Angew. Chem. Int. Ed. 2015, 54, 13758–13763. 10.1002/anie.201506998 PMC4648037 26404498

[20] T. P. Robinson , S.-K. Lo , D. De Rosa , S. Aldridge , J. M. Goicoechea , Chem. Eur. J. 2016, 22, 15712–15724. 27628576 10.1002/chem.201603135 PMC5095867

[21] T. P. Robinson , D. De Rosa , S. Aldridge , J. M. Goicoechea , Chem. Eur. J. 2017, 23, 15455–15465. 28865168 10.1002/chem.201703119

[22] P. Wang , Q. Zhu , Y. Wang , G. Zeng , J. Zhu , C. Zhu , Chin. Chem. Lett. 2021, 32, 1432–1436.

[23] A. J. Pistner , H. W. Moon , A. Silakov , H. P. Yennawar , A. T. Radosevich , Inorg. Chem. 2017, 56, 8661–8668. 28661124 10.1021/acs.inorgchem.7b00657

[24] S. M. McCarthy , Y.-C. Lin , D. Devarajan , J. W. Chang , H. P. Yennawar , R. M. Rioux , D. H. Ess , A. T. Radosevich , J. Am. Chem. Soc. 2014, 136, 4640–4650. 24597970 10.1021/ja412469e

[25] M. Driess , N. Muresan , K. Merz , M. Päch , Angew. Chem. Int. Ed. 2005, 44, 6734–6737. 10.1002/anie.200501990 16175648

[26] G. Zeng , S. Maeda , T. Taketsugu , S. Sakaki , Angew. Chem. Int. Ed. 2014, 53, 4633–4637. 10.1002/anie.201311104 24668586

[27] S. Volodarsky , R. Dobrovetsky , Chem. Commun. 2018, 54, 6931–6934. 10.1039/c8cc02423e 29862397

[28] A. Pal , K. Vanka , Inorg. Chem. 2016, 55, 558–565. 26700074 10.1021/acs.inorgchem.5b01074

[29] A. J. Arduengo , C. A. Stewart , Chem. Rev. 1994, 94, 1215–1237.

[30] D. Bawari , S. Volodarsky , Y. Ginzburg , K. Jaiswal , P. Joshi , R. Dobrovetsky , Chem. Commun. 2022, 58, 12176–12179. 10.1039/d2cc04359a 36226583

[31] A. Hentschel , A. Brand , P. Wegener , W. Uhl , Angew. Chem. Int. Ed. 2018, 57, 832–835. 10.1002/anie.201711373 29171723

[32] A. Brand , P. Wegener , A. Hepp , W. Uhl , Organometallics 2020, 39, 1384–1392.

[33] K. Chulsky , I. Malahov , D. Bawari , R. Dobrovetsky , J. Am. Chem. Soc. 2023, 145, 3786–3794. 36738474 10.1021/jacs.2c13318 PMC9936586

[34] M. B. Kindervater , K. M. Marczenko , U. Werner-Zwanziger , S. S. Chitnis , Angew. Chem. Int. Ed. 2019, 58, 7850–7855. 10.1002/anie.201903354 30945403

[35] G. T. Cleveland , A. T. Radosevich , Angew. Chem. Int. Ed. 2019, 58, 15005–15009. 10.1002/anie.201909686 PMC6779506 31469492

[36] N. L. Dunn , M. Ha , A. T. Radosevich , J. Am. Chem. Soc. 2012, 134, 11330–11333. 22746974 10.1021/ja302963p

[37] K. Lee , A. V. Blake , A. Tanushi , S. M. McCarthy , D. Kim , S. M. Loria , C. M. Donahue , K. D. Spielvogel , J. M. Keith , S. R. Daly , A. T. Radosevich , Angew. Chem. Int. Ed. 2019, 58, 6993–6998. 10.1002/anie.201901779 PMC6513703 30901511

[38] S. Lim , A. T. Radosevich , J. Am. Chem. Soc. 2020, 142, 16188–16193. 32909747 10.1021/jacs.0c07580

[39] Y.-C. Lin , J. C. Gilhula , A. T. Radosevich , Chem. Sci. 2018, 9, 4338–4347. 29780566 10.1039/c8sc00929e PMC5944378

[40] Y.-C. Lin , E. Hatzakis , S. M. McCarthy , K. D. Reichl , T.-Y. Lai , H. P. Yennawar , A. T. Radosevich , J. Am. Chem. Soc. 2017, 139, 6008–6016. 28398750 10.1021/jacs.7b02512 PMC5778438

[41] J. M. Lipshultz , Y. Fu , P. Liu , A. T. Radosevich , Chem. Sci. 2021, 12, 1031–1037. 10.1039/d0sc05620k PMC8179051 34163869

[42] H. W. Moon , A. Maity , A. T. Radosevich , Organometallics 2021, 40, 2785–2791.

[43] A. Tanushi , A. T. Radosevich , J. Am. Chem. Soc. 2018, 140, 8114–8118. 29923715 10.1021/jacs.8b05156 PMC6033636

[44] C. te Grotenhuis , J. T. Mattos , A. T. Radosevich , Phosphorus Sulfur Silicon Relat. Elem. 2020, 195, 940–946.

[45] W. Zhao , S. M. McCarthy , T. Y. Lai , H. P. Yennawar , A. T. Radosevich , J. Am. Chem. Soc. 2014, 136, 17634–17644. 25401723 10.1021/ja510558d

[46] M. K. Mondal , L. Zhang , Z. Feng , S. Tang , R. Feng , Y. Zhao , G. Tan , H. Ruan , X. Wang , Angew. Chem. Int. Ed. 2019, 58, 15829–15833. 10.1002/anie.201910139 31478328

[47] S. Volodarsky , D. Bawari , R. Dobrovetsky , Angew. Chem. Int. Ed. 2022, 61, e202208401. 10.1002/anie.202208401 PMC9541694 35830679

[48] Q. J. Bruch , A. Tanushi , P. Müller , A. T. Radosevich , J. Am. Chem. Soc. 2022, 144, 21443–21447. 36378626 10.1021/jacs.2c10200 PMC9712262

[49] S. J. Hwang , A. Tanushi , A. T. Radosevich , J. Am. Chem. Soc. 2020, 142, 21285–21291. 33306370 10.1021/jacs.0c11161 PMC7806272

[50] J. Cui , Y. Li , R. Ganguly , A. Inthirarajah , H. Hirao , R. Kinjo , J. Am. Chem. Soc. 2014, 136, 16764–16767. 25390290 10.1021/ja509963m

[51] J. Cui , Y. Li , R. Ganguly , R. Kinjo , Chem. Eur. J. 2016, 22, 9976–9985. 27283866 10.1002/chem.201600935

[52] G. Baccolini , E. Mezzina , P. E. Todesco , E. Foresti , J. Chem. Soc. Chem. Commun. 1988, 304–305.

[53] G. Baccolini , E. Mezzina , P. E. Todesco , J. Chem. Soc. Perkin Trans. 1 1988, 3281–3283.

[54] G. Baccolini , C. A. Mosticchio , E. Mezzina , C. Rizzoli , P. Sgarabotto , Heteroat. Chem. 1993, 4, 319–322.

[55] J. Abbenseth , O. P. E. Townrow , J. M. Goicoechea , Angew. Chem. Int. Ed. 2021, 60, 23625–23629. 10.1002/anie.202111017 PMC8596738 34478227

[56] J. I. van der Vlugt , Top. Organomet. Chem. 2021, 68, 135–179.

[57] J. I. van der Vlugt , Chem. Eur. J. 2019, 25, 2651–2662. 30084211 10.1002/chem.201802606 PMC6471147

[58] O. R. Luca , R. H. Crabtree , Chem. Soc. Rev. 2013, 42, 1440–1459. 22975722 10.1039/c2cs35228a

[59] G. Zeng , S. Maeda , T. Taketsugu , S. Sakaki , J. Am. Chem. Soc. 2016, 138, 13481–13484. 27690395 10.1021/jacs.6b07274

[60] D. Yang , P. Bao , Z. Yang , Z. Chen , S. Sakaki , S. Maeda , G. Zeng , ChemCatChem 2021, 13, 3925–3929.

[61] Q. Zhu , R. Qiu , S. Dong , G. Zeng , J. Zhu , Chem. Asian J. 2021, 16, 2063–2067. 34101364 10.1002/asia.202100427

[62] J. Underhill , E. S. Yang , T. Schmidt-Räntsch , W. K. Myers , J. M. Goicoechea , J. Abbenseth , Chem. Eur. J. 2023, 29, e202203266. 36281622 10.1002/chem.202203266 PMC10098518

[63] See Supporting Information for all experimental details, spectra and single crystal X-ray data. See Supporting Information for all experimental details. Deposition Numbers 2245882 (**2**), 2245883 (**3** ^ **Me** ^), 2245884 (**3** ^ * **i** * **Pr** ^), 2245885 (**4** ^ * **i** * **Pr** ^), 2245886 (**6**), 2245887 (**7**⋅hex), 2245888 (**8**), 2245889 (**9**), 2245890 (**10**), 2245891 (**11**), 2245892 (**12**), 2245893 (**14**), 2245894 (**15**) and 2245895 (for **16**) contains the supplementary crystallographic data for this paper. These data are provided free of charge by the joint Cambridge Crystallographic Data Centre and Fachinformationszentrum Karlsruhe Access Structures service.

[64] K. M. Marczenko , J. A. Zurakowski , M. B. Kindervater , S. Jee , T. Hynes , N. Roberts , S. Park , U. Werner-Zwanziger , M. Lumsden , D. N. Langelaan , S. S. Chitnis , Chem. Eur. J. 2019, 25, 16414–16424. 31574185 10.1002/chem.201904361

[65] S. E. Frazier , R. P. Nielsen , H. H. Sisler , Inorg. Chem. 1964, 3, 292–294.

[66] S. F. Spangenberg , H. H. Sisler , Inorg. Chem. 1969, 8, 1006–1010.

[67] J. C. Summers , H. H. Sisler , Inorg. Chem. 1970, 9, 862–869.

[68] A. Schumann , F. Reiß , H. Jiao , J. Rabeah , J.-E. Siewert , I. Krummenacher , H. Braunschweig , C. Hering-Junghans , Chem. Sci. 2019, 10, 7859–7867. 31853345 10.1039/c9sc02322d PMC6839504

[69] J.-E. Siewert , A. Schumann , C. Hering-Junghans , Dalton Trans. 2021, 50, 15111–15117. 34611690 10.1039/d1dt03095g

[70] L. Falivene , Z. Cao , A. Petta , L. Serra , A. Poater , R. Oliva , V. Scarano , L. Cavallo , Nat. Chem. 2019, 11, 872–879. 31477851 10.1038/s41557-019-0319-5

[71] S. Volodarsky , I. Malahov , D. Bawari , M. Diab , N. Malik , B. Tumanskii , R. Dobrovetsky , Chem. Sci. 2022, 13, 5957–5963. 35685804 10.1039/d2sc01060g PMC9132080

[72] A. Okuniewski , D. Rosiak , J. Chojnacki , B. Becker , Polyhedron 2015, 90, 47–57.

[73] D. W. Allen , B. F. Taylor , J. Chem. Soc. Dalton Trans. 1982, 51–54.

[74] A. Sarbajna , V. S. V. S. N. Swamy , V. H. Gessner , Chem. Sci. 2021, 12, 2016–2024. 10.1039/d0sc03278f PMC8179322 34163963

[75] P. Pyykkö , J. Phys. Chem. A 2015, 119, 2326–2337. 25162610 10.1021/jp5065819

[76] L. Alig , M. Fritz , S. Schneider , Chem. Rev. 2019, 119, 2681–2751. 30596420 10.1021/acs.chemrev.8b00555

[77] R. J. Somerville , J. Campos , Eur. J. Inorg. Chem. 2021, 3488–3498. 34690540 10.1002/ejic.202100460 PMC8518731

[78] B. Chatterjee , W.-C. Chang , S. Jena , C. Werlé , ACS Catal. 2020, 10, 14024–14055.

[79] M. R. Elsby , R. T. Baker , Chem. Soc. Rev. 2020, 49, 8933–8987. 33164012 10.1039/d0cs00509f

[80] E. R. M. Habraken , A. R. Jupp , M. B. Brands , M. Nieger , A. W. Ehlers , J. C. Slootweg , Eur. J. Inorg. Chem. 2019, 2436–2442. 31423108 10.1002/ejic.201900169 PMC6687039

[81] T. Higashi , S. Kusumoto , K. Nozaki , Chem. Rev. 2019, 119, 10393–10402. 31408323 10.1021/acs.chemrev.9b00262

[82] J. R. Khusnutdinova , D. Milstein , Angew. Chem. Int. Ed. 2015, 54, 12236–12273. 10.1002/anie.201503873 26436516

[83] S. Yao , M. Brym , C. van Wüllen , M. Driess , Angew. Chem. Int. Ed. 2007, 46, 4159–4162. 10.1002/anie.200700398 17436257

[84] T. Oishi , L. I. Lugo-Fuentes , Y. Jing , J. O. C. Jimenez-Halla , J. Barroso-Flores , M. Nakamoto , Y. Yamamoto , N. Tsunoji , R. Shang , Chem. Commun. 2021, 12, 15603–15608. 10.1039/d1sc05135k PMC8654027 35003590

[85] F. Dankert , M. Fischer , C. Hering-Junghans , Dalton Trans. 2022, 51, 11267–11276. 35766522 10.1039/d2dt01575g

[86] J. E. Griffiths , A. B. Burg , J. Am. Chem. Soc. 1960, 82, 1507–1508.

[87] W. J. Bailey , R. B. Fox , J. Org. Chem. 1963, 28, 531–534.

[88] W. J. Bailey , R. B. Fox , J. Org. Chem. 1964, 29, 1013–1017.

[89] I. G. M. Campbell , I. D. R. Stevens , Chem. Commun. 1966, 505–506.

[90] B. Hoge , S. Neufeind , S. Hettel , W. Wiebe , C. Thösen , J. Organomet. Chem. 2005, 690, 2382–2387.

[91] A. Christiansen , C. Li , M. Garland , D. Selent , R. Ludwig , A. Spannenberg , W. Baumann , R. Franke , A. Börner , Eur. J. Org. Chem. 2010, 2733–2741.

[92] B. G. Janesko , H. C. Fisher , M. J. Bridle , J.-L. Montchamp , J. Org. Chem. 2015, 80, 10025–10032. 26372089 10.1021/acs.joc.5b01618

[93] A. Hubbard , T. Okazaki , K. K. Laali , J. Org. Chem. 2008, 73, 316–319. 18067314 10.1021/jo701937e

