# Supplementary material for: Reactivity of a Strictly T‐Shaped Phosphine Ligated by an Acridane Derived NNN Pincer Ligand
Source: Chemistry. 2023 May 23;29(39):e202300818. doi: 10.1002/chem.202300818 (PMC10947599; doi:10.1002/chem.202300818)
Supplement: Supplementary file 1 — Supporting Information [file CHEM-29-0-s001.pdf]

Chemistry–A European Journal

Supporting Information

Reactivity of a Strictly T-Shaped Phosphine Ligated by an Acridane Derived NNN Pincer Ligand

Aaron J. King, Josh Abbenseth,* and Jose M. Goicoechea*

Contents

1. Experimental section 2
1.1 General experimental methods 2
1.2 Synthesis and characterisation of reported compounds 3
1.2.1 Synthesis of **1**^{Me} 3
1.2.2 Synthesis of **2** 6
1.2.3 Synthesis of **3**^{Me} 9
1.2.4 Synthesis of **3**^{iPr} 12
1.2.5a Synthesis of **4**^{iPr} 15
1.2.6 Synthesis of **5** 18
1.2.7 Synthesis of **6** 21
1.2.8a Synthesis of **7** 24
1.2.9a Synthesis of **8** 27
1.2.10 Synthesis of **9** 31
1.2.11 Synthesis of **10** 34
1.2.12 Synthesis of **11** 37
1.2.13 Synthesis of **12** 40
1.2.14 Synthesis of **13** 43
1.2.15 Synthesis of **14** 48
1.2.16 Synthesis of **15** 51
1.2.17 Synthesis of **16** 54
2. Additional NMR spectra 57
2.1 Reaction between **3**^{Me} and PMe_3 57
2.2 Reaction between acridane derived ligand **1**^{iPr} and PCl_3 59
2.3 Dimerisation of **4**^{iPr} under ambient conditions 60
2.4 Reaction between **4**^{iPr} and O_2 60
2.5 Reaction between **4**^{iPr} and S_8 61
2.6 Reaction between **12** and benzaldehyde 62
2.7 Decomposition of **13** 63
2.8 D_2O activation product characterisation (**15**^{D}) 65
2.9 Variable temperature NMR study of **2** 66
2.10 ATIR spectrum of **15** 67
3. Single crystal X-ray diffraction data 68
4. References 77

1. Experimental section

1.1 General experimental methods

Synthetic methods:

All reactions and product manipulations were carried out using standard Schlenk-line techniques under an inert atmosphere of argon, or in a dinitrogen filled glovebox (MBraun UNIlab glovebox maintained at < 0.1 ppm H_2O and < 0.1 ppm O_2). Hexane (Sigma Aldrich, HPLC grade), pentane (Sigma Aldrich, HPLC grade), benzene (Rathburn, HPLC grade), dichloromethane (Sigma Aldrich, HPLC grade), acetonitrile (Sigma Aldrich, HPLC grade) diethyl ether (Sigma Aldrich, HPLC grade) and toluene (Sigma Aldrich, HPLC grade) were purified using an MBraun SPS-800 solvent system. Ethyl acetate (Sigma Aldrich, HPLC grade) and methanol (Sigma Aldrich anhydrous, 99.8%) was used as provided. THF (Sigma Aldrich, HPLC grade) was distilled over sodium/benzophenone. C_6D_6 (Sigma Aldrich, 99.5%) and CD_2Cl_2 (Sigma Aldrich, 99.5%) was degassed and dried over CaH_2. d_8-THF (Sigma Aldrich, 99.5%) was degassed and dried over sodium/potassium alloy. All dry solvents were stored under argon in gas-tight ampoules over activated 3 Å molecular sieves. 2,7,9,9-tetramethyl-4,5-dinitro-9,10-dihydroacridine[1], **1**^{iPr}[1] and mesityl azide[2] were synthesised according to their referenced literature procedures. The following were used as received from their commercial supplier without further purification: palladium on carbon (10 w%, Sigma Aldrich), H_2 (Sigma Aldrich), paraformaldehyde (Merck), sodium methoxide (Sigma Aldrich), sodium borohydride (Sigma Aldrich), phosphorus pentabromide (Acros), trimethylphosphine (Strem), magnesium powder (Sigma Aldrich), potassium *tert*-butoxide (Sigma Aldrich), 4,7,13,16,21,24-hexaoxa-1,10-diazabicyclo[8.8.8]hexacosane (Merck), methyl triflate (Apollo Scientific), oxygen (Linde), 2,4-bis(4-methoxyphenyl-1,3,2,4-dithiadiphosphetane-2,4-disulfide (Alfa Aesar), sulfur (Sigma Aldrich), selenium (Sigma Aldrich), 2*H*-1,3,2-benzodioxaborole (Alfa Aesar), 2 M hydrochloric acid in diethyl ether (diluted prior to use, Alfa Aesar) and deuterium oxide (Sigma Aldrich). Phosphorus trichloride (Sigma Aldrich) was distilled and degassed prior to use. Triethylamine (TCI) was degassed, dried over CaH_2 and distilled prior to use. Benzaldehyde (Sigma Aldrich) was distilled prior to use. Pyridine N-oxide (Sigma Aldrich) was sublimed prior to use.

Characterisation techniques:

NMR samples were prepared inside an inert atmosphere glovebox in NMR tubes fitted with a gas-tight valve. NMR spectra were acquired on either a Bruker AVIII 400 MHz NMR spectrometer (^{1}H 400 MHz, ^{11}B 128 MHz, ^{31}P 162 MHz), a Bruker AVIII 500 spectrometer (^{1}H 500 MHz, ^{31}P 202 MHz, ^{19}F 470 MHz, ^{77}Se 95 MHz) or a Bruker Avance NEO 600 MHz NMR spectrometer with a broadband helium cryoprobe (^{1}H 600 MHz, ^{31}P 243 MHz, ^{13}C 151 MHz). ^{1}H and ^{13}C NMR spectra were referenced to their respective solvent resonance (^{1}H NMR C_6D_6: δ = 7.16 ppm, CD_2Cl_2: δ = 5.32 ppm, d_8-THF: δ = 3.58 ppm; ^{13}C NMR C_6D_6: δ = 128.1 ppm, CD_2Cl_2: δ = 53.8 ppm, d_8-THF: δ = 67.6 ppm). ^{11}B NMR spectra were externally referenced to $BF_3{\cdot}Et_2O$ in C_6D_6. ^{19}F NMR spectra were externally referenced to $CFCl_3$. ^{31}P NMR spectra were externally referenced to an 85% solution of H_3PO_4 in H_2O. High-resolution mass spectrometry was acquired using a Thermo Exactive High-Resolution Orbitrap FTMS instrument equipped with a Acquity TUV Detector, with a Waters Acquity Ultraperformance LC system used for sample delivery. Elemental analyses were carried out by Elemental Analysis Services Team (London Metropolitan University, U.K.). Samples (approx. 5 mg) were submitted in flame sealed glass tubes. Infrared spectra were acquired on a Thermo Scientific iS5 FTIR spectrometer using an iD3 ATR stage.

1.2 Synthesis and characterisation of reported compounds

1.2.1 Synthesis of 1^{Me}

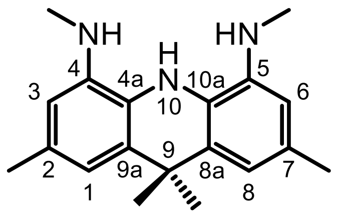

2,7,9,9-tetramethyl-4,5-dinitro-9,10-dihydroacridine (663 mg, 2.03 mmol, 1.00 eq.) and palladium on carbon (108 mg, 0.102 mmol palladium, 10.0 wt% palladium, 5.00 mol% palladium) are added to a gas tight ampoule and suspended in ethyl acetate (20 mL). The suspension is freeze-pump-thaw degassed four times, then refilled with dihydrogen gas (1 atm, excess) and stirred at 50 °C for 18 hours. The mixture is cooled to room temperature and the solvent is removed *in vacuo*. The residue is extracted into methanol (3 × 10 mL) and filtered. The mixture is cooled to 0 °C then paraformaldehyde (608 mg, 20.6 mmol, 10.0 eq.) is added followed by sodium methoxide (25 w% in methanol, 1.85 mL, 8.10 mmol, 4.00 eq.). The mixture is warmed to reflux and stirred for 1 hour. The mixture is then cooled to 0 °C and sodium borohydride (843 mg, 22.3 mmol, 11.0 eq.) is added in three portions over the course of 15 minutes. The mixture is warmed to reflux and stirred for 2 hours. The mixture is cooled to room temperature and the solvent is removed *in vacuo*. The residue is extracted into benzene (3 × 10 mL) and filtered. Lyophilisation from a concentrated benzene solution affords $\mathbf{1^{Me}}$ (474 mg, 1.61 mmol, 79%) as a white powder.

^{1}H NMR (600 MHz, C_6D_6): δ = 6.94 (2H, s, $C_{1/8}H$), 6.46 (2H, s, $C_{3/6}H$), 5.69 (1H, br s, $N_{10}H$), 2.55 (2H, br m, $C_{4/5}NH$), 2.46 (6H, d, $^3J_{H–H}$ = 5.5 Hz, N–CH_3), 2.36 (6H, s, $C_{2/7}(CH_3)$), 1.73 ppm (6H, s, $C_9(CH_3)_2$).

^{13}C{^{1}H} NMR (151 MHz, C_6D_6): δ = 136.3 (s, $C_{4/5}$), 129.7 (s, $C_{2/7}$), 129.2 (s, $C_{8a/9a}$), 126.7 (s, $C_{4a/10a}$), 117.5 (s, $C_{1/8}$), 111.3 (s, $C_{3/6}$), 36.2 (s, C_9), 31.9 (s, $C_9(CH_3)_2$), 31.1 (s, $C_{4/5}N(CH_3)$), 21.5 ppm (s, $C_{2/7}(CH_3)$).

HRMS (ESI) *m/z* calcd. for $C_{19}H_{25}N_3$+H^+: 296.2121 [*M*+H]$^+$; found: 296.2116.

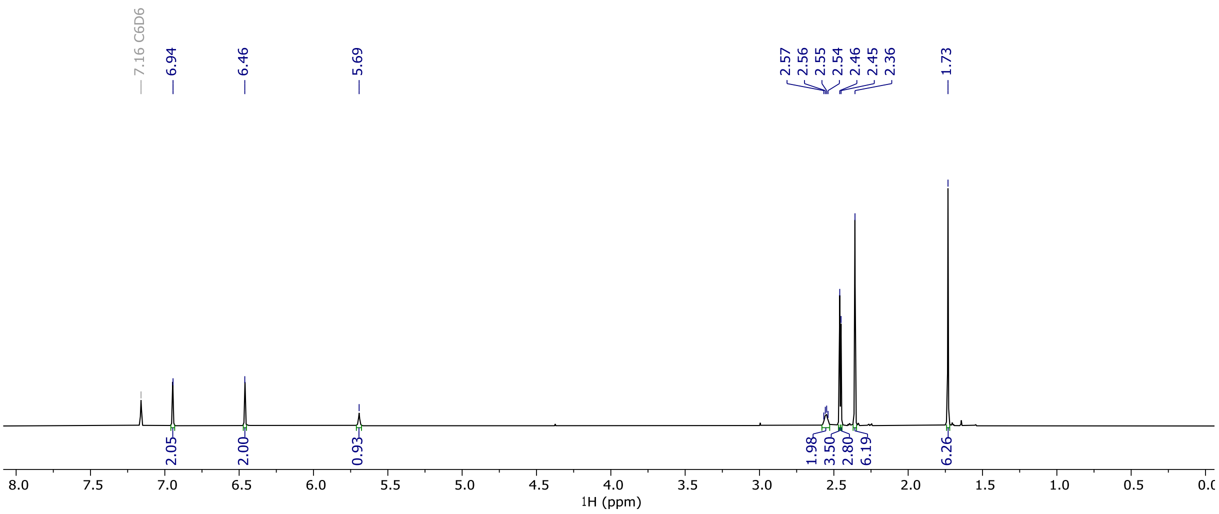

Figure S1: ^{1}H NMR spectrum of $\mathbf{1^{Me}}$ in C_6D_6.

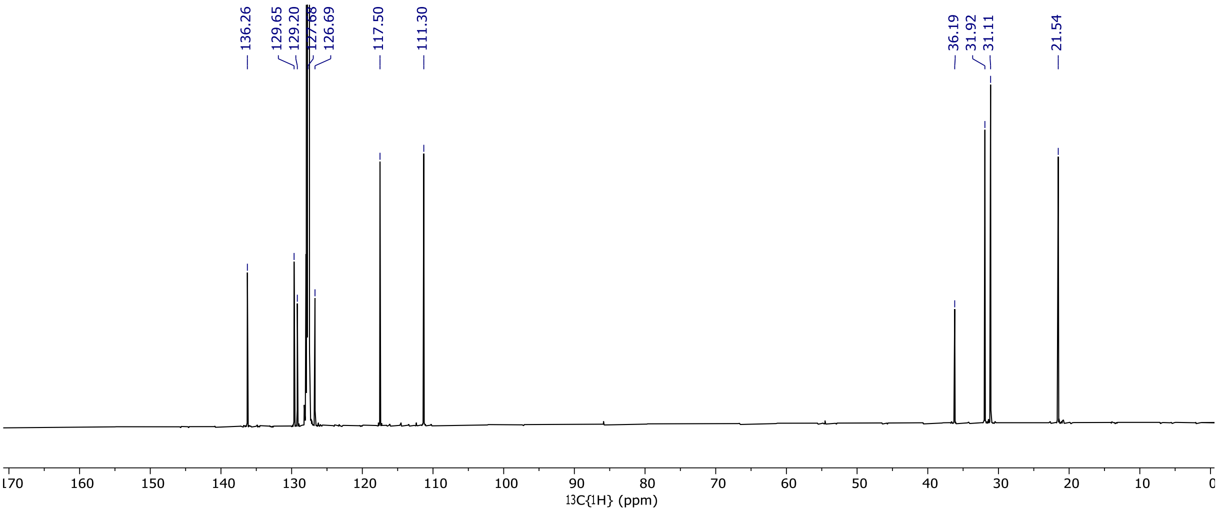

Figure S2: ^{13}C{^{1}H} NMR spectrum of $\mathbf{1^{Me}}$ in C_6D_6.

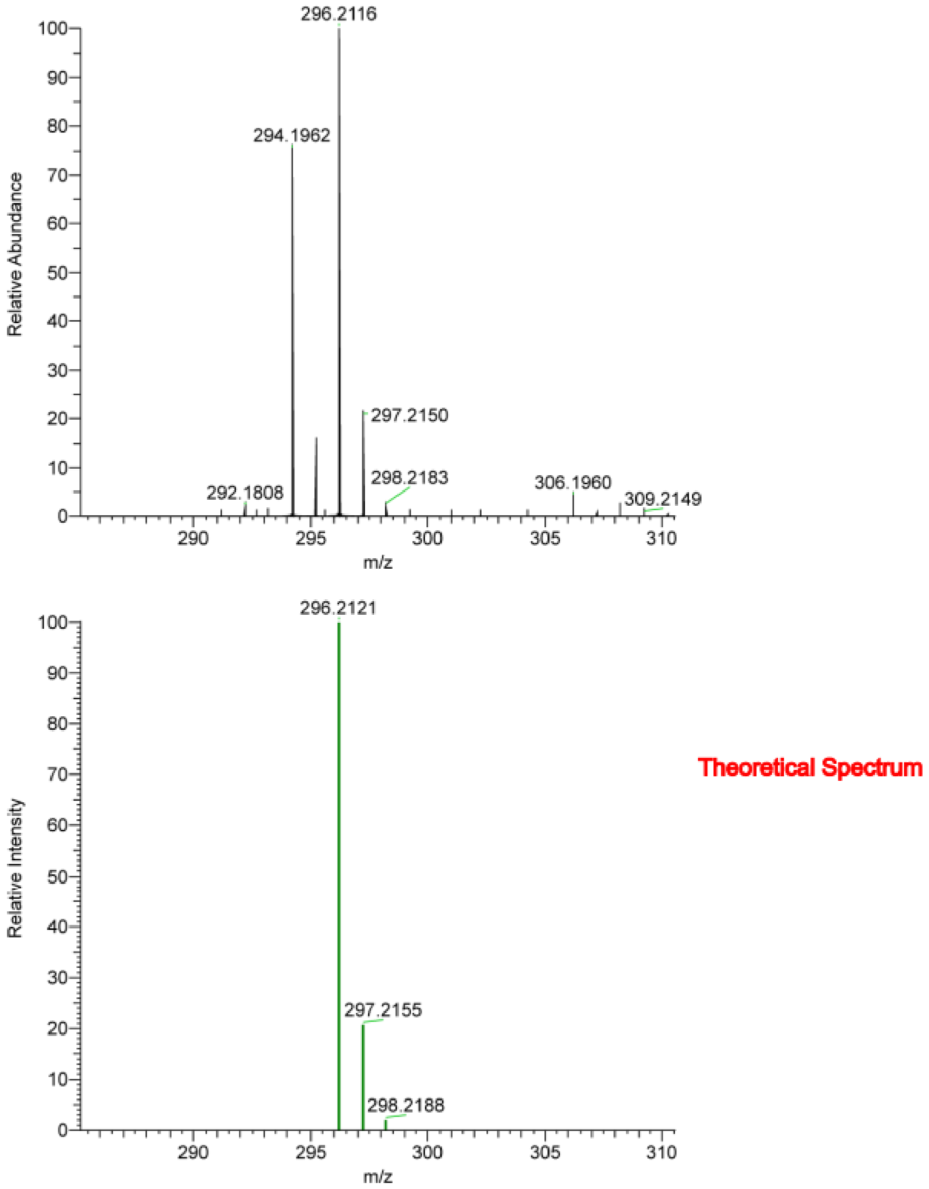

Figure S3: Top: high-resolution mass spectrum of **1**$^{\mathbf{Me}}$ showing the [*M*+H]$^+$ ion at *m/z* 296.2116. Bottom: theoretical spectrum.

1.2.2 Synthesis of 2

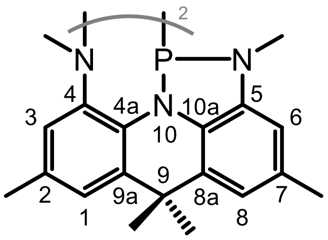

1^Me (50.0 mg, 0.169 mmol, 1.00 eq.) is dissolved in dichloromethane (5 mL) and cooled to −78 °C. Phosphorus trichloride (0.573 M in hexane, 0.30 mL, 0.169 mmol, 1.00 eq.) is added followed by triethylamine (83.0 µL, 0.593 mmol, 3.50 eq.). The mixture is gradually warmed to room temperature and stirred for 16 hours, resulting in the generation of a pale-yellow solution and off-white precipitate. The solution is then filtered and the precipitate extracted with dichloromethane (3 × 2 mL). The solvent is removed from the combined extracts *in vacuo* and the resulting residue washed with acetonitrile (3 × 2 mL) at −30 °C. The product is dried under vacuum, providing **2** as a white powder (26.0 mg, 41.1 µmol, 49%).

^{1}H NMR (600 MHz, C_6D_6): δ = 7.19 (1H, s, C_1H), 6.85 (1H, s, C_3H), 6.72 (1H, s, C_8H), 6.26 (1H, s, C_6H), 2.79 (6H, d, $^3J_{P–H}$ = 8.0 Hz, $C_5N(CH_3)$), 2.63 (6H, d, $^3J_{P–H}$ = 1.3 Hz, $C_4N(CH_3)$), 2.41 (6H, s, $C_7(CH_3)$), 2.24 (6H, s, $C_2(CH_3)$), 1.78 (6H, s, $C_9(CH_3)$), 1.65 ppm (6H, s, $C_9(CH_3)$).

^{1}H{^{31}P} NMR (500 MHz, C_6D_6): as above, except δ = 2.79 (6H, s, $C_5N(CH_3)$), 2.63 ppm (6H, s, $C_4N(CH_3)$).

^{13}C{^{1}H} NMR (151 MHz, C_6D_6): δ = 139.2 (d, $^2J_{C–P}$ = 7.9 Hz, C_5), 133.8 (dd, $^2J_{C–P}$ = 5.2 Hz, $^3J_{C–P}$ = 2.7 Hz, C_{4a}), 133.2 (m, C_{Ar})*, 131.0 (s, C_7), 129.9 (d, $^4J_{C–P}$ = 2.5 Hz, C_2), 129.2 (d, $^2J_{C–P}$ = 8.2 Hz, C_{10a}), 127.5 (d, $^3J_{C–P}$ = 7.0 Hz, C_3), 126.3 (d, $^4J_{C–P}$ = 2.6 Hz, C_1), 126.2 (s, C_{Ar})*, 115.3 (s, C_8)**, 105.4 (s, C_6)**, 36.8 (s, C_9), 36.6 (s, $C_9(CH_3)$), 34.7 (dd, $^{5TS}J_{C–P}$ = 5.5 Hz, $^2J_{C–P}$ = 13.7 Hz, $C_4N(CH_3)$)***, 32.1 (d, $^2J_{C–P}$ = 32.2 Hz, $C_5N(CH_3)$), 31.9 (s, $C_9(CH_3)$), 22.1 (s, $C_7(CH_3)$), 21.0 ppm (s, $C_2(CH_3)$).

* C_{8a} and C_{9a} environments could not be distinguished, but correspond to either of these resonances. This is due to the observation of a ^{1}H-^{13}C HMBC cross-peak between each of these and both $C_9(CH_3)$ signals.

** assigned by comparison to **13**, **14** and **15**.

*** TS = through space coupling to phosphorus in close proximity.[3]

^{31}P NMR (243 MHz, C_6D_6): δ = 112.1 ppm (m).

^{31}P{^{1}H} NMR (162 MHz, C_6D_6): δ = 112.1 ppm (s).

Elemental analysis calcd. (%) for $C_{38}H_{44}N_6P_2$: C 70.57, H 6.86, N 12.99; found: C 70.63, H 7.01, N 11.45.

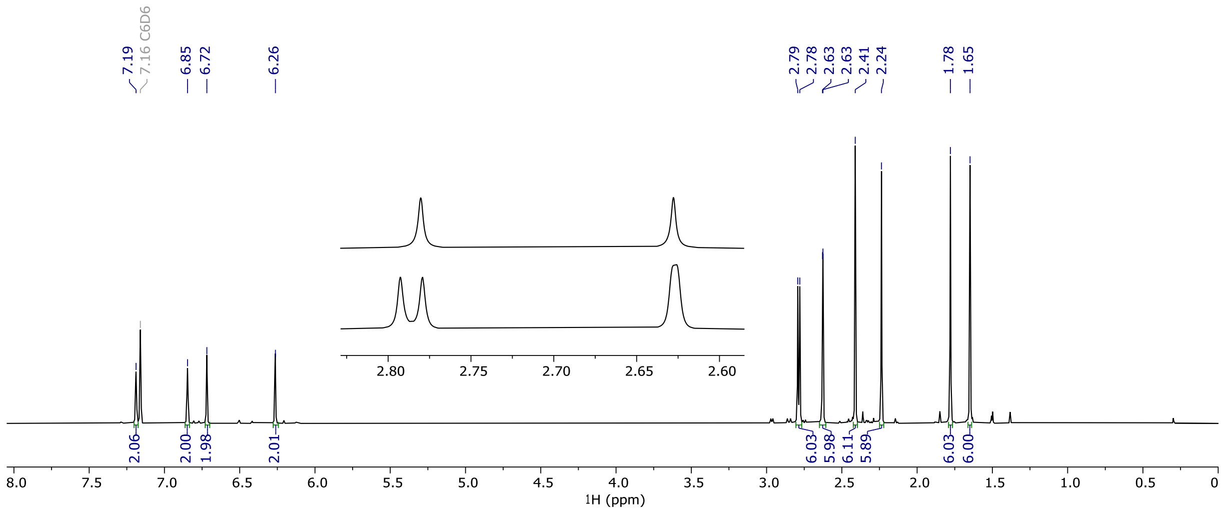

Figure S4: ^{1}H NMR spectrum of **2** in C_6D_6. Inset: top is $^{1}H\{^{31}P\}$ and bottom is ^{1}H NMR spectrum enhanced between 2.83 – 2.58 ppm.

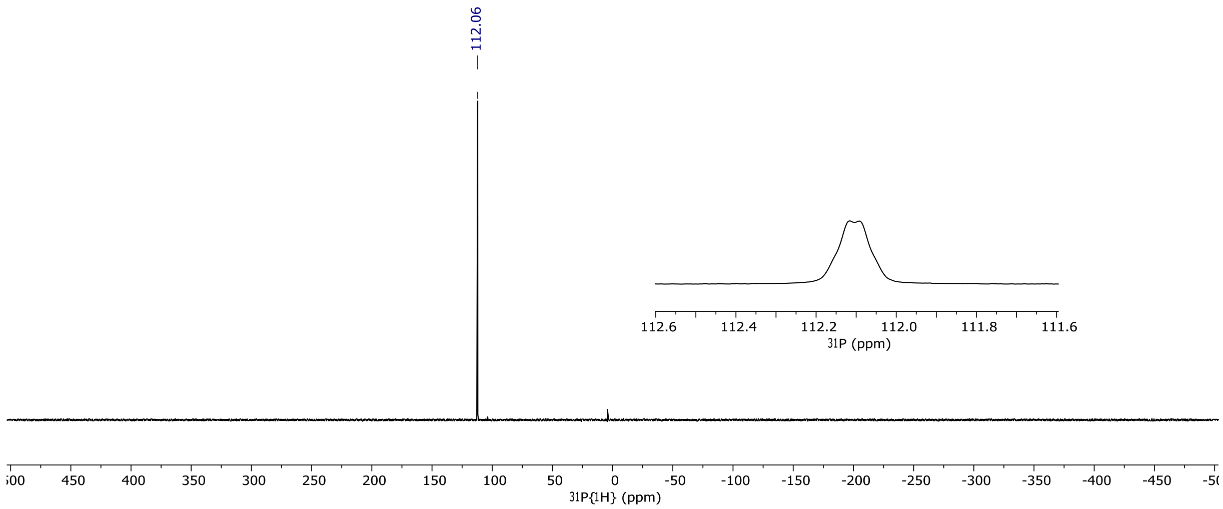

Figure S5: $^{31}P\{^{1}H\}$ NMR spectrum of **2** in C_6D_6. Inset: ^{31}P NMR spectrum enhanced between 112.60 - 111.60 ppm.

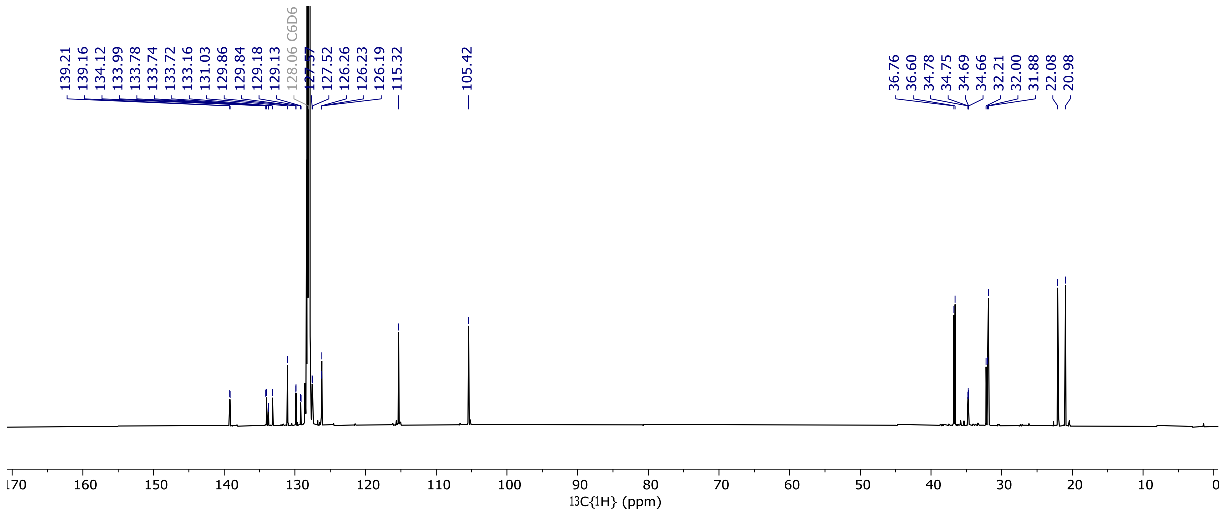

Figure S6: $^{13}C\{^{1}H\}$ NMR spectrum of **2** in C_6D_6.

1.2.3 Synthesis of 3^{Me}

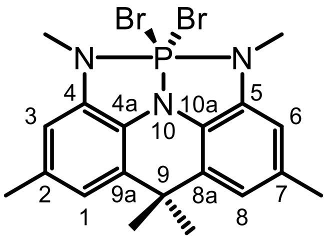

Phosphorus pentabromide (72.9 mg, 0.169 mmol, 1.00 eq.) is dissolved in toluene (10 mL) and cooled to −78 °C**.** Triethylamine (71.0 µL, 0.507 mmol, 3.00 eq.) is added. A solution of $\mathbf{1^{Me}}$ (50 mg, 0.169 mmol, 1.00 eq.) in toluene (10 mL) is also cooled to −78 °C and added dropwise to the phosphorus pentabromide solution. The mixture is gradually warmed to room-temperature and stirred for 16 hours resulting in the generation of an orange solution and off-white precipitate. The solution is then filtered and the precipitate extracted with toluene (3 × 5 mL). The solvent is removed *in vacuo* and the residue dissolved in hexane and cooled to −30 °C for 5 days resulting in the generation of orange solid (an unknown impurity). The solution is then filtered and the solvent removed *in vacuo*. Lyophilisation from a concentrated benzene solution provides $\mathbf{3^{Me}}$ as an orange powder (52.0 mg, 0.108 mmol, 64%).

^{1}H NMR (600 MHz, C_6D_6): δ = 6.67 (2H, s, $C_{1/8}H$), 6.12 (2H, s, $C_{3/6}H$), 2.95 (6H, d, $^{3}J_{P–H}$ = 21.1 Hz, $C_{4/5}N(CH_3)$), 2.26 (6H, s, $C_{2/7}(CH_3)$), 1.51 ppm (6H, s, $C_9(CH_3)_2$).

^{1}H{^{31}P} NMR (500 MHz, C_6D_6): as above, expect δ = 2.95 ppm (6H, s, $C_{4/5}N(CH_3)$).

^{13}C{^{1}H} NMR (151 MHz, C_6D_6): δ = 135.1 (d, $^{2}J_{C–P}$ = 12.2 Hz, $C_{4/5}$), 133.6 (s, $C_{2/7}$), 129.7 (d, $^{3}J_{C–P}$ = 9.2 Hz, $C_{8a/9a}$), 116.5 (d, $^{4}J_{C–P}$ = 1.8 Hz, $C_{1/8}$), 116.1 (d, $^{2}J_{C–P}$ = 13.4 Hz, $C_{4a/10a}$), 105.4 (d, $^{3}J_{C–P}$ = 7.6 Hz, $C_{3/6}$), 36.9 (d, $^{4}J_{C–P}$ = 1.7 Hz, C_9), 32.6 (s, $C_9(CH_3)_2$), 31.5 (d, $^{2}J_{C–P}$ = 5.5 Hz, $C_{4/5}N(CH_3)$), 22.6 ppm (s, $C_{2/7}(CH_3)$).

^{31}P NMR (243 MHz, C_6D_6): δ = −107.5 ppm (sept, $^{3}J_{P–H}$ = 21.1 Hz).

^{31}P{^{1}H} NMR (162 MHz, C_6D_6): δ = −107.6 ppm (s).

Elemental analysis calcd. (%) for $C_{19}H_{22}Br_2N_3P$: C 47.23, H 4.59, N 8.70; found: C 47.32, H 4.94, N 8.03.

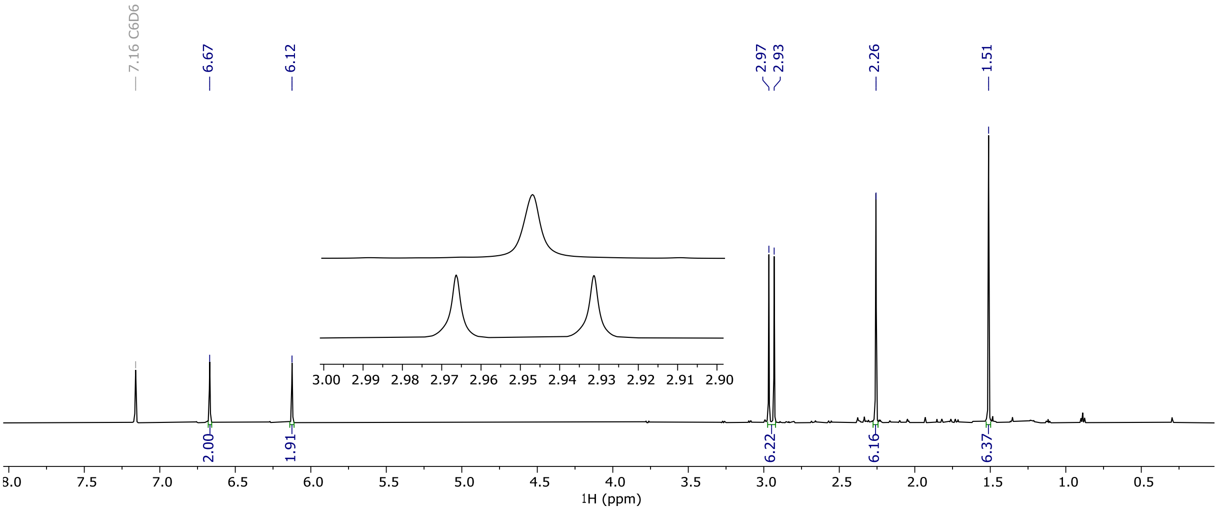

Figure S7: ^{1}H NMR spectrum of $\mathbf{3^{Me}}$ in C_6D_6. Inset: top is $^{1}H\{^{31}P\}$ and bottom is ^{1}H NMR spectrum enhanced between 3.00 – 2.90 ppm.

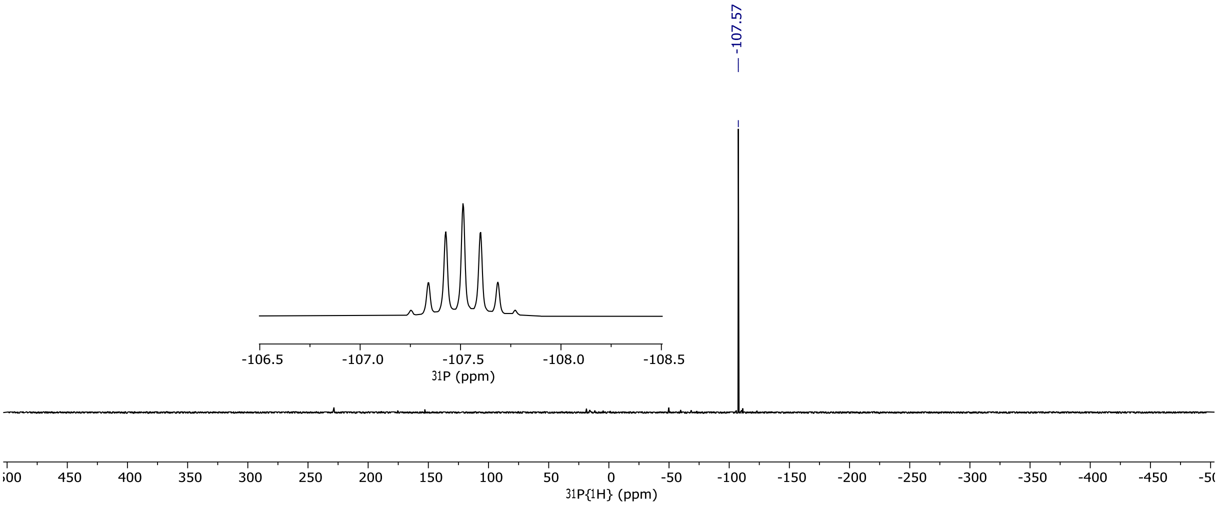

Figure S8: $^{31}P\{^{1}H\}$ NMR spectrum of $\mathbf{3^{Me}}$ in C_6D_6. Inset: ^{31}P NMR spectrum enhanced between −106.50 - −108.50 ppm.

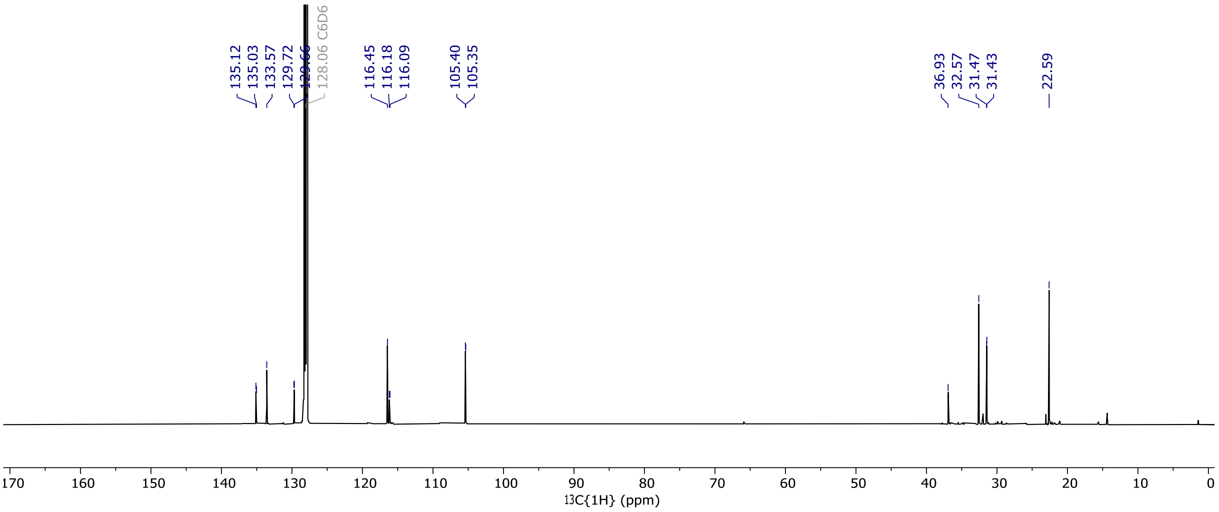

Figure S9: $^{13}C\{^1H\}$ NMR spectrum of $\mathbf{3^{Me}}$ in C_6D_6.

1.2.4 Synthesis of 3^{iPr}

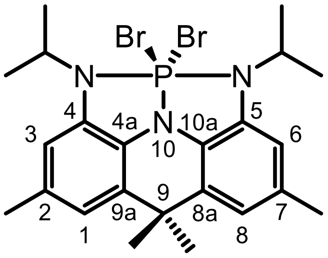

A solution of phosphorous pentabromide (366 mg, 0.853 mmol, 1.00 eq.) in toluene (10 mL) is cooled to −78 °C. Triethylamine (0.600 mL, 4.27 mmol, 5.00 eq.) is added, followed by the addition of a −78 °C solution of $\mathbf{1}^{iPr}$ (300 mg, 0.853 mmol, 1.00 eq.) in toluene (10 mL). The resulting bright green solution is gradually warmed to room temperature and stirred for 16 hours over which the solution turned dark brown along with the precipitation of a light brown solid. The mixture is filtered and the precipitate extracted with toluene (3 × 5 mL). The solvent is removed *in vacuo* providing an orange-brown residue, which is then washed with diethyl ether (3 × 5 mL) at −78 °C. The resulting bright orange powder is dried *in vacuo* to provide $\mathbf{3}^{iPr}$ (250 mg, 0.464 mmol, 54%).

^{1}H NMR (500 MHz, C_6D_6): δ = 6.64 (2H, s, $C_{1/8}H$), 6.62 (2H, s, $C_{3/6}H$), 4.74 (2H, d-sept, $^{3}J_{P-H}$ = 27.3 Hz, $^{3}J_{H-H}$ = 6.9 Hz, $C_{4/5}NCH(CH_3)_2$), 2.26 (6H, s, $C_{2/7}(CH_3)$), 1.53 (12H, d, $^{3}J_{H-H}$ = 6.9 Hz, $C_{4/5}NCH(CH_3)_2$), 1.52 ppm (6H, s, $C_9(CH_3)_2$).

^{1}H{^{31}P} NMR (500 MHz, C_6D_6): as above, except δ = 4.74 ppm (2H, sept, $^{3}J_{H-H}$ = 6.9 Hz, $C_{4/5}NCH(CH_3)_2$).

^{13}C{^{1}H} NMR (151 MHz, C_6D_6): δ = 133.0 (d, $^{4}J_{C-P}$ = 1.7 Hz, $C_{2/7}$), 132.7 (d, $^{2}J_{C-P}$ = 13.7 Hz, $C_{4/5}$), 130.4 (d, $^{3}J_{C-P}$ = 9.4 Hz, $C_{8a/9a}$), 116.7 (d, $^{2}J_{C-P}$ = 14.3 Hz, $C_{4a/10a}$), 115.8 (m, $C_{1/8}$), 107.9 (d, $^{3}J_{C-P}$ = 9.9 Hz, $C_{3/6}$), 46.7 (d, $^{2}J_{C-P}$ = 6.0 Hz, $C_{4/5}NCH(CH_3)_2$), 36.7 (d, $^{4}J_{C-P}$ = 1.8 Hz, C_9), 33.0 (s, $C_9(CH_3)_2$), 22.7 (s, $C_{2/7}(CH_3)$), 19.0 ppm (d, $^{3}J_{C-P}$ = 6.5 Hz, $C_{4/5}NCH(CH_3)_2$).

^{31}P NMR (202 MHz, C_6D_6): δ = −120.1 ppm (t, $^{3}J_{P-H}$ = 27.3 Hz).

^{31}P{^{1}H} NMR (162 MHz, C_6D_6): δ = −120.1 ppm (s).

Elemental analysis calcd. (%) for $C_{23}H_{30}Br_2N_3P$: C 51.22, H 5.61, N 7.79; found: C 51.08, H 5.26, N 7.41.

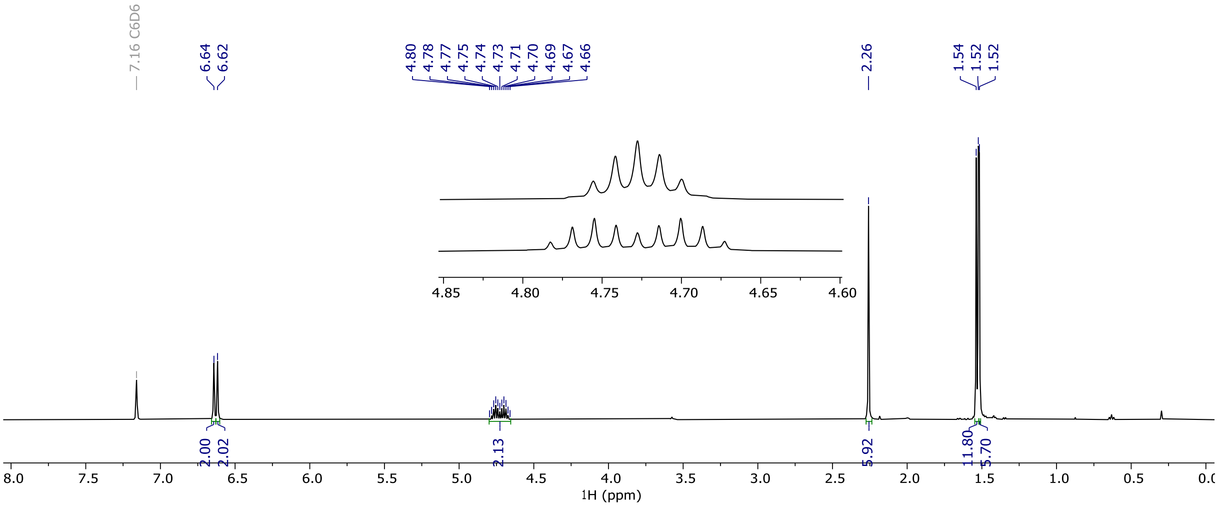

Figure S10: ^{1}H NMR spectrum of $\mathbf{3}^{\boldsymbol{i}\mathbf{Pr}}$ in C_6D_6. Inset: top is $^{1}H\{^{31}P\}$ and bottom is ^{1}H NMR spectrum enhanced between 4.85 – 4.60 ppm.

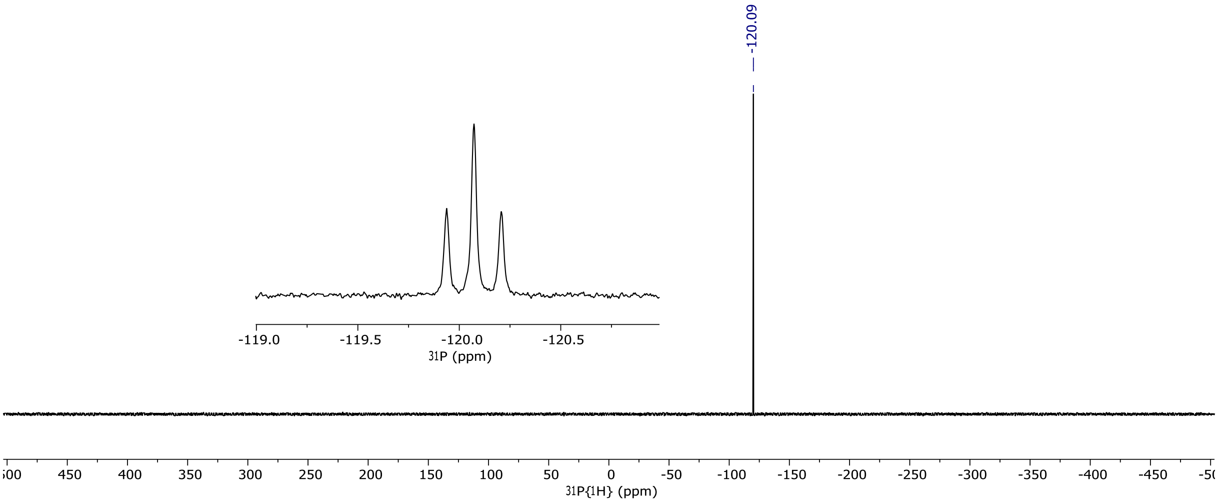

Figure S11: $^{31}P\{^{1}H\}$ NMR spectrum of $\mathbf{3}^{\boldsymbol{i}\mathbf{Pr}}$ in C_6D_6. Inset: ^{31}P NMR spectrum enhanced between −119.00 - −121.00 ppm.

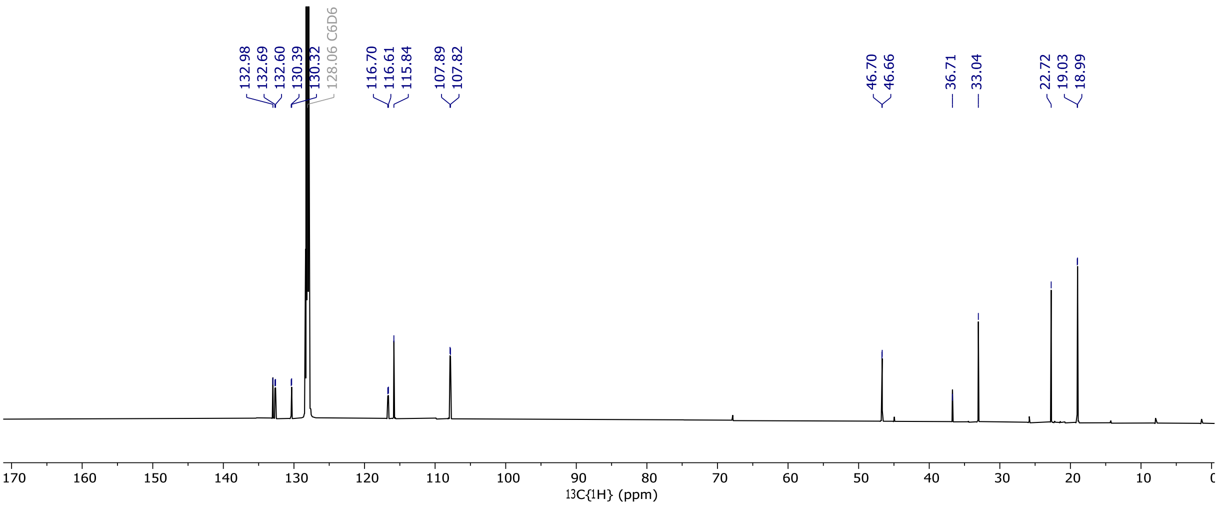

Figure S12: $^{13}C\{^{1}H\}$ NMR spectrum of **3*^{iPr}*** in C_6D_6.

1.2.5a Synthesis of 4^{iPr}

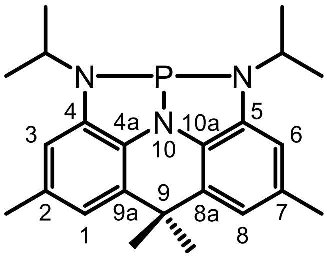

3^{iPr} (200 mg, 0.371 mmol, 1.00 eq.) is dissolved in toluene (10 mL). A solution of trimethylphosphine (0.19 mL, 1.85 mmol, 5.00 eq.) in toluene (1 mL) is added and the mixture stirred for 15 minutes, resulting in the generation of a bright orange solution and off-white precipitate. The solution is filtered and the precipitate extracted with toluene (3 × 5 mL). The solvent is removed *in vacuo*. Lyophilisation from a concentrated benzene solution provides **4^{iPr}** as an orange powder (127 mg, 0.335 mmol, 90%).

^{1}H NMR (500 MHz, C_6D_6): δ = 6.86 (2H, s, $C_{1/8}H$), 6.79 (2H, s, $C_{3/6}H$), 4.42 (2H, apparent sept, $^{3}J_{H–H}$ = 6.9 Hz, $C_{4/5}NCH(CH_3)_2$)*, 2.37 (6H, s, $C_{2/7}(CH_3)$), 1.72 (6H, s, $C_9(CH_3)_2$), 1.52 ppm (12H, d, $^{3}J_{H–H}$ = 6.9 Hz, $C_{4/5}NCH(CH_3)_2$).

* Coupling to phosphorus was not observed.

^{1}H{^{31}P} NMR (500 MHz, C_6D_6): as above.

^{13}C{^{1}H} NMR (151 MHz, C_6D_6): δ = 135.9 (d, $^{2}J_{C–P}$ = 12.6 Hz, $C_{4/5}$), 133.5 (d, $^{3}J_{C–P}$ = 4.6 Hz, $C_{8a/9a}$), 133.4 (s, $C_{2/7}$), 130.5 (d, $^{2}J_{C–P}$ = 11.2 Hz, $C_{4a/10a}$), 115.7 (s, $C_{1/8}$), 109.1 (s, $C_{3/6}$), 45.1 (d, $^{2}J_{C–P}$ = 20.9 Hz, $C_{4/5}NCH(CH_3)_2$), 38.3 (s, C_9), 31.3 (s, $C_9(CH_3)_2$), 23.6 (d, $^{3}J_{C–P}$ = 6.4 Hz, $C_{4/5}NCH(CH_3)_2$), 22.6 ppm (s, $C_{2/7}(CH_3)$).

^{31}P NMR (202 MHz, C_6D_6): δ = 145.0 ppm (br s).

^{31}P{^{1}H} NMR (162 MHz, C_6D_6): δ = 145.0 ppm (br s).

Elemental analysis calcd. (%) for $C_{23}H_{30}N_3P$: C 72.80, H 7.97, N 11.07; found: C 72.35, H 7.79, N 10.21.

1.2.5b Alternative route to 4^{iPr}

3^{iPr} (100 mg, 0.185 mmol, 1.00 eq.) and magnesium powder (92 mg, 3.709 mmol, 20.0 eq.) is dissolved in tetrahydrofuran (5 mL) and stirred at room temperature for 15 minutes. The solvent is removed *in vacuo* and the product extracted into pentane (3 × 2 mL) and filtered. The solvent is removed *in vacuo* from the combined extracts. Lyophilisation from a concentrated benzene solution provides **4^{iPr}** as an orange powder (63.0 mg, 0.166 mmol, 90%).

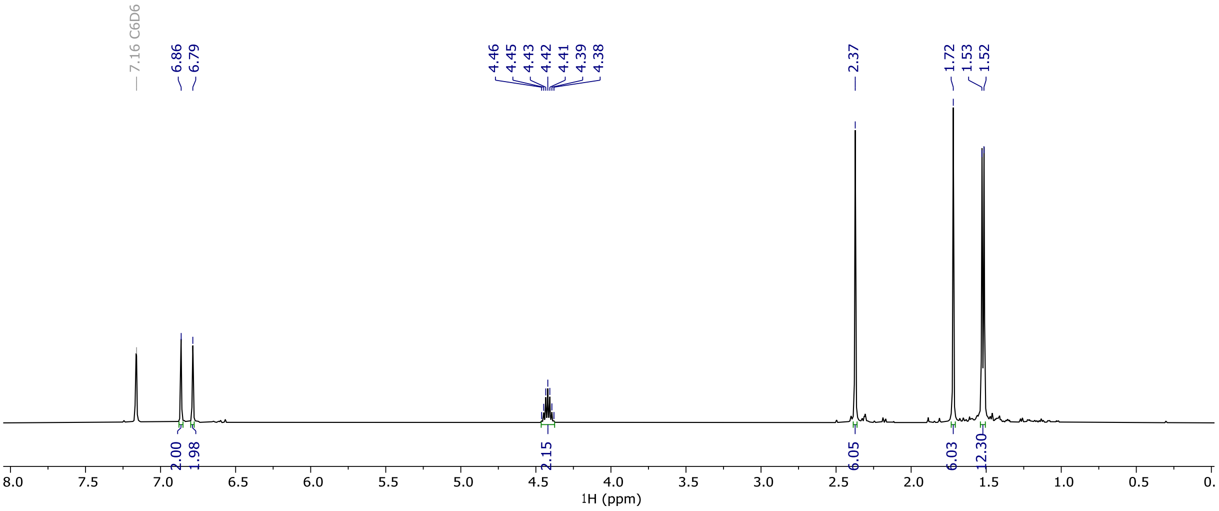

Figure S13: ^{1}H NMR spectrum of **4^{iPr}** in C_6D_6.

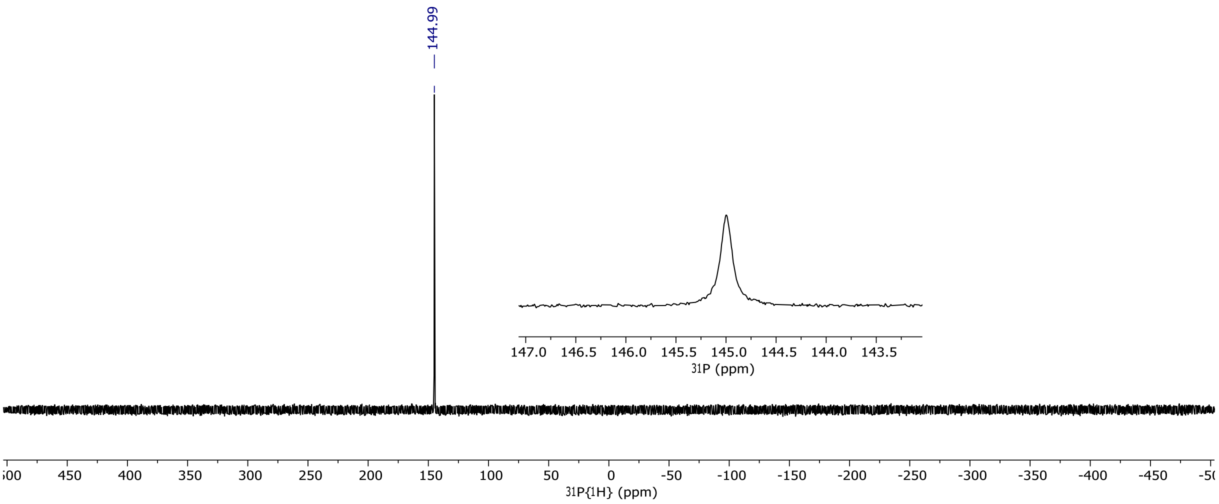

Figure S14: ^{31}P{^{1}H} NMR spectrum of **4^{iPr}** in C_6D_6. Inset: ^{31}P NMR spectrum enhanced between 147.00 - 143.00 ppm.

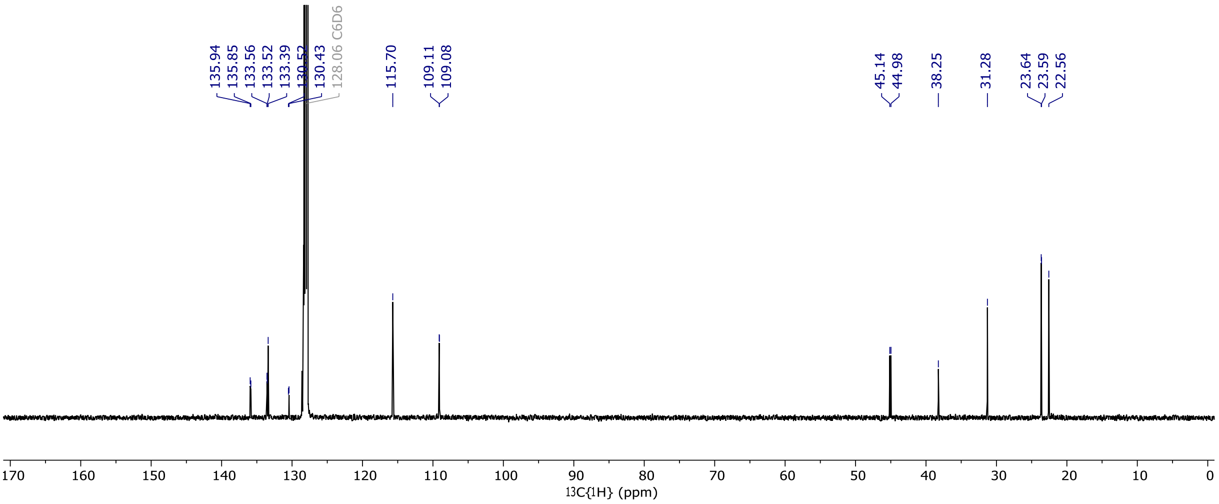

Figure S15: $^{13}C\{^{1}H\}$ NMR spectrum of **4**^{iPr} in C_6D_6.

1.2.6 Synthesis of 5

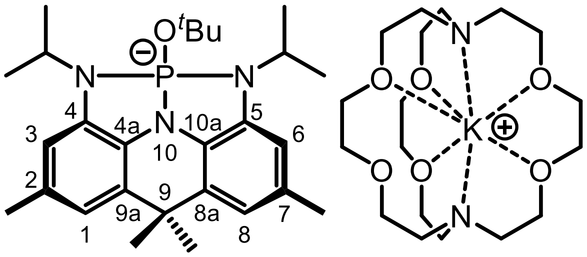

Potassium *tert*-butoxide (3.1 mg, 27.8 µmol, 1.00 eq.) and 4,7,13,16,21,24-hexaoxa-1,10-diazabicyclo[8.8.8]hexacosane (10.5 mg, 27.8 µmol, 1.00 eq.) is dissolved in diethyl ether (0.5 mL). The resulting solution is added to **4^{iPr}** (10.5 mg, 27.8 µmol, 1.00 eq.) in a J. Young NMR tube producing a yellow solution. The solution is filtered and half of the solvent is removed *in vacuo*. Crystallisation from this concentrated diethyl ether at room temperature yields **5** (11.0 mg, 12.7 µmol, 46%) as yellow crystals.

^{1}H NMR (500 MHz, d_8-THF): δ = 5.82 (2H, s, $C_{1/8}H$), 5.57 (2H, s, $C_{3/6}H$), 3.70 (2H, apparent sept, $^3J_{H–H}$ = 6.6 Hz, $C_{4/5}NCH(CH_3)_2$)*, 3.37 (14H, s, CH^{crypt}), 3.32 (14H, t, $^3J_{H–H}$ = 4.6 Hz, CH^{crypt}), 2.36 (14H, t, $^3J_{H–H}$ = 4.6 Hz, CH^{crypt}), 2.11 (6H, s, $C_{2/7}(CH_3)$), 1.60 (3H, s, $C_9(CH_3)$), 1.50 (3H, s, $C_9(CH_3)$), 1.41 (6H, d, $^3J_{H–H}$ = 6.7 Hz, $C_{4/5}NCH(CH_3)$), 1.35 (6H, d, $^3J_{H–H}$ = 6.7 Hz, $C_{4/5}NCH(CH_3)$), 0.90 ppm (9H, s, P–OC($CH_3)_3$).

* Coupling to phosphorus was not observed

^{1}H{^{31}P} NMR (500 MHz, d_8-THF): as above.

^{13}C{^{1}H} NMR (151 MHz, d_8-THF): δ = 142.3 (d, $^2J_{C–P}$ = 13.2 Hz, C_4), 128.0 (s, $C_{4a/10a}$),127.9 (s, $C_{2/7}$), 125.3 (s, $C_{8a/9a}$), 107.0 (s, $C_{1/8}$H), 100.8 (d, $^3J_{C–P}$ = 2.8 Hz, $C_{3/6}$H), 73.0 (d, $^2J_{C–P}$ = 21.6 Hz, P–O$C(CH_3)_3$), 71.3 (s, C^{crypt}), 68.5 (s, C^{crypt}), 54.8 (s, C^{crypt}), 46.8 (d, $^2J_{C–P}$ = 8.3 Hz, $C_{4/5}NCH(CH_3)_2$), 36.8 (m, C_9), 36.8 (s, $C_9(CH_3)$), 33.7 (m, $C_9(CH_3)$), 32.1 (d, $^2J_{C–P}$ = 4.1 Hz, P–OC($CH_3)_3$), 23.1 (s, $C_{2/7}(CH_3)$), 23.0 (d, $^3J_{C–P}$ = 3.3 Hz, $C_{4/5}NCH(CH_3)$), 22.9 ppm (d, $^3J_{C–P}$ = 6.1 Hz, $C_{4/5}NCH(CH_3)$).

^{31}P NMR (243 MHz, d_8-THF): δ = 50.2 ppm (s).

^{31}P{^{1}H} NMR (162 MHz, d_8-THF): δ = 50.0 ppm (s).

Elemental analysis calcd. (%) for $C_{45}H_{75}K_1N_5O_7P$: C 62.26, H 8.71, N 8.07; found: C 61.81, H 8.77, N 7.52.

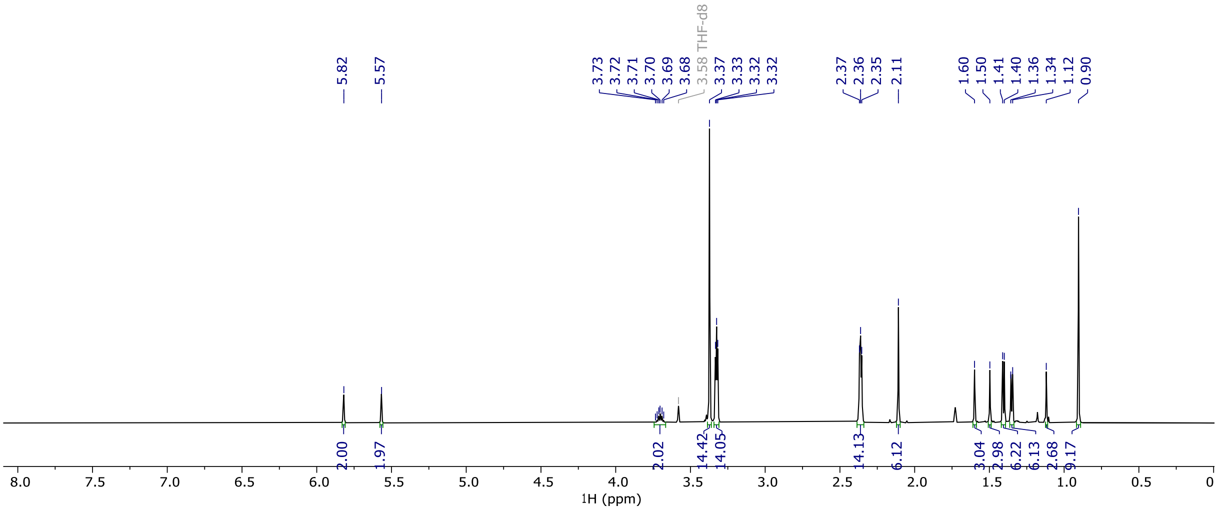

Figure S16: ^{1}H NMR spectrum of **5** in d_8-THF.

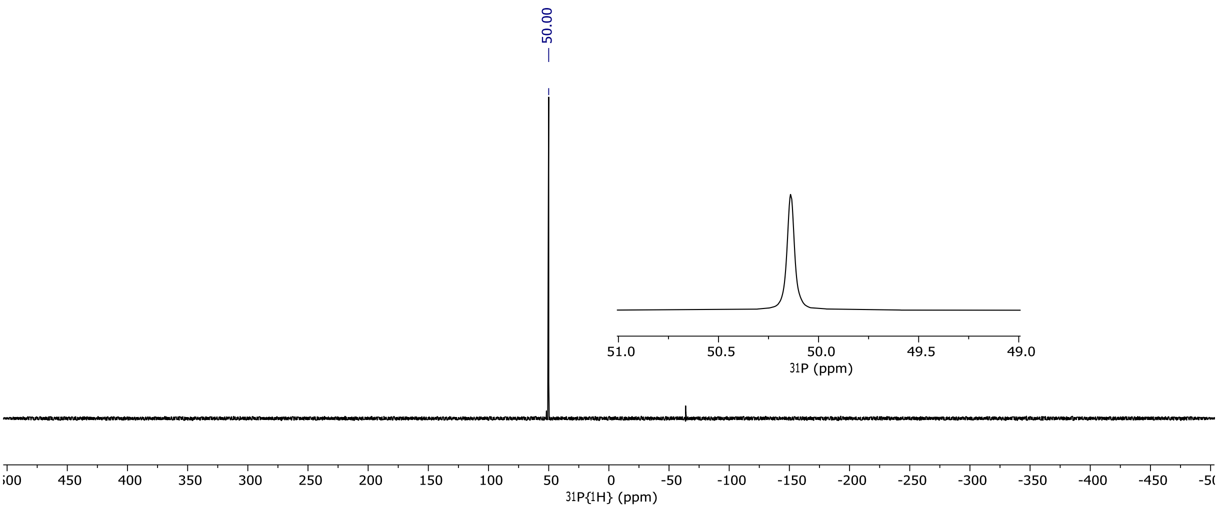

Figure S17: $^{31}P\{^{1}H\}$ NMR spectrum of **5** in d_8-THF. Inset: ^{31}P NMR spectrum enhanced between 51.00 - 49.00 ppm.

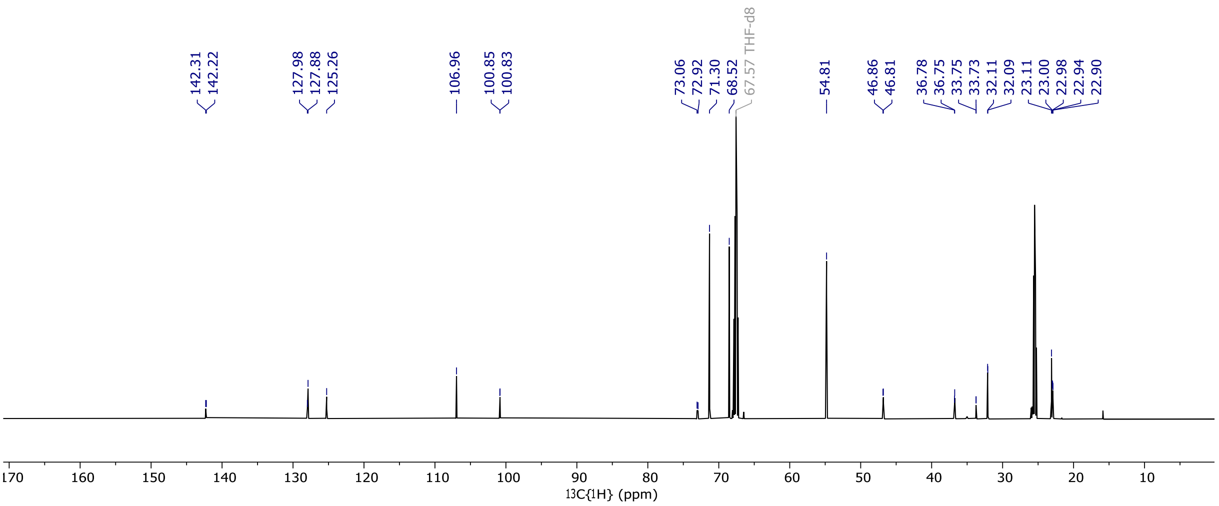

Figure S18: $^{13}C\{^1H\}$ NMR spectrum of **5** in d_8-THF.

1.2.7 Synthesis of 6

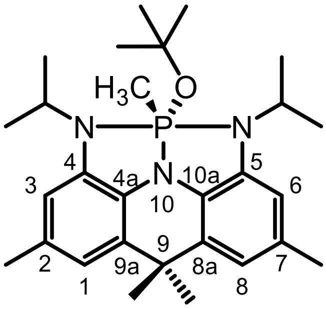

Potassium *tert*-butoxide (3.12 mg, 27.8 µmol, 1.00 eq.) and 4,7,13,16,21,24-Hexaoxa-1,10-diazabicyclo[8.8.8]hexacosane (10.5 mg, 27.8 µmol, 1.00 eq.) is dissolved in diethyl ether (0.5 mL). The resulting solution is added to $\mathbf{4}^{\boldsymbol{iPr}}$ (10.5 mg, 27.8 µmol, 1.00 eq.) in a J. Young NMR tube producing a yellow solution. The solvent is removed *in vacuo*. A solution of methyl triflate (3.0 µL, 27.8 µmol, 1.00 eq.) in d_6-benzene (0.5 mL) is added, producing a colourless solution. The solvent is removed *in vacuo* and the product extracted into pentane (3 × 0.5 mL). The solvent is removed, then yellow crystals of **6** are grown from a concentrated hexane solution at −30 °C (5.0 mg, 10.7 µmol, 38%).

^{1}H NMR (600 MHz, C_6D_6): δ = 6.64 (2H, s, $C_{1/8}\mathit{H}$), 6.46 (2H, s, $C_{3/6}\mathit{H}$), 3.75 (2H, apparent oct, $^{3}J_{P–H}$ = 6.7 Hz, $^{3}J_{H–H}$ = 6.7 Hz, $C_{4/5}NC\mathit{H}(CH_3)_2$), 2.43 (6H, s, $C_{2/7}(C\mathit{H}_3)$), 1.83 (3H, s, $C_9(C\mathit{H}_3)$), 1.81 (3H, s, $C_9(C\mathit{H}_3)$), 1.63 (3H, d, $^{2}J_{P–H}$ = 16.6 Hz, P–$C\mathit{H}_3$), 1.46 (6H, d, $^{3}J_{H–H}$ = 6.7 Hz, $C_{4/5}NCH(C\mathit{H}_3)$), 1.35 (6H, d, $^{3}J_{H–H}$ = 6.8 Hz, $C_{4/5}NCH(C\mathit{H}_3)$), 1.04 ppm (9H, s, P–$OC(C\mathit{H}_3)_3$).

$^{1}H\{^{31}P\}$ NMR (600 MHz, C_6D_6): as above, except δ = 3.75 (2H, sept, $^{3}J_{H–H}$ = 6.7 Hz, $C_{4/5}NC\mathit{H}(CH_3)_2$), 1.63 ppm (3H, s, P–$C\mathit{H}_3$).

$^{13}C\{^{1}H\}$ NMR (151 MHz, C_6D_6): δ = 135.2 (d, $^{2}J_{C–P}$ = 8.7 Hz, $\mathit{C}_{4/5}$), 134.1 (d, $^{2}J_{C–P}$ = 19.2 Hz, C_4), 131.6 (s, $\mathit{C}_{2/7}$), 128.6 (s, $\mathit{C}_{8a/9a}$), 118.9 (d, $^{2}J_{C–P}$ = 12.7 Hz, $\mathit{C}_{4a/10a}$), 112.1 (s, $\mathit{C}_{1/8}$), 105.7 (d, $^{3}J_{C–P}$ = 4.4 Hz, $\mathit{C}_{3/6}$), 80.0 (d, $^{2}J_{C–P}$ = 11.2 Hz, P–$O\mathit{C}(CH_3)_3$), 45.9 (d, $^{2}J_{C–P}$ = 2.6 Hz, $C_{4/5}NCH(CH_3)_2$), 36.4 (m, C_9), 35.2 (s, $C_9(\mathit{C}H_3)$), 32.8 (s, $C_9(\mathit{C}H_3)$), 30.5 (d, $^{3}J_{C–P}$ = 5.9 Hz, P–$OC(\mathit{C}H_3)_3$), 25.8 (d, $^{1}J_{C–P}$ = 179.4 Hz, P–$\mathit{C}H_3$), 23.0 (s, $C_{2/7}(\mathit{C}H_3)$), 20.6 (d, $^{3}J_{C–P}$ = 2.2 Hz, $C_{4/5}NCH(\mathit{C}H_3)$), 20.5 ppm (s, $C_{4/5}NCH(\mathit{C}H_3)$).

^{31}P NMR (243 MHz, C_6D_6): δ = −39.4 ppm (m).

$^{31}P\{^{1}H\}$ NMR (162 MHz, C_6D_6): δ = −39.4 ppm (s).

Elemental analysis calcd. (%) for $C_{28}H_{42}N_3OP$: C 71.92, H 9.05, N 8.99; found: C 72.20, H 9.46, N 8.47.

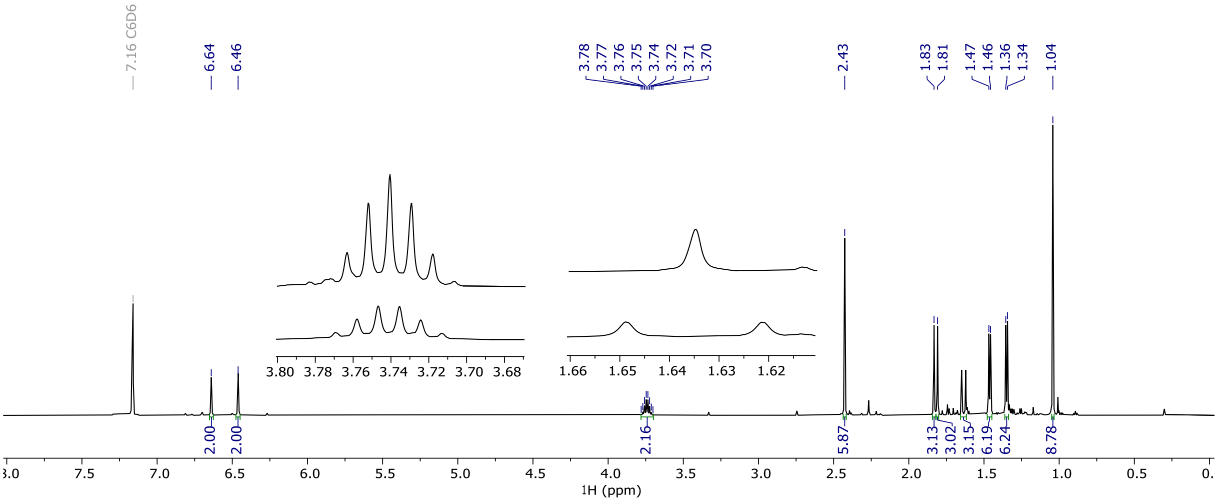

Figure S19: ^{1}H NMR spectrum of **6** in C_6D_6. Left inset: top is $^{1}H\{^{31}P\}$ and bottom is ^{1}H NMR spectrum enhanced between 3.80 – 3.66 ppm. Right inset: top is $^{1}H\{^{31}P\}$ and bottom is ^{1}H NMR spectrum enhanced between 1.66 – 1.61 ppm.

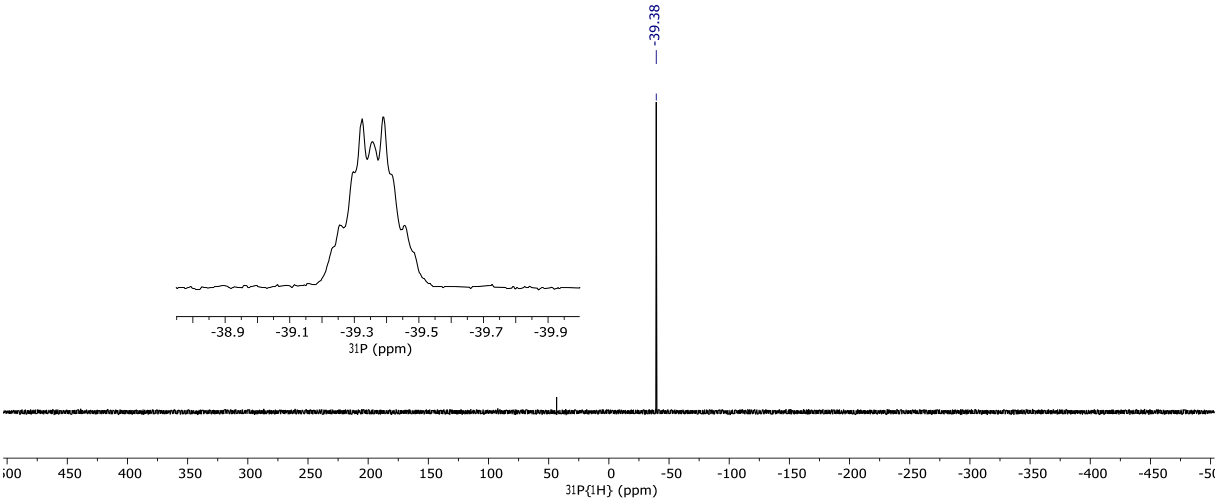

Figure S20: $^{31}P\{^{1}H\}$ NMR spectrum of **6** in C_6D_6. Inset: ^{31}P NMR spectrum enhanced between –38.75 – –39.80 ppm.

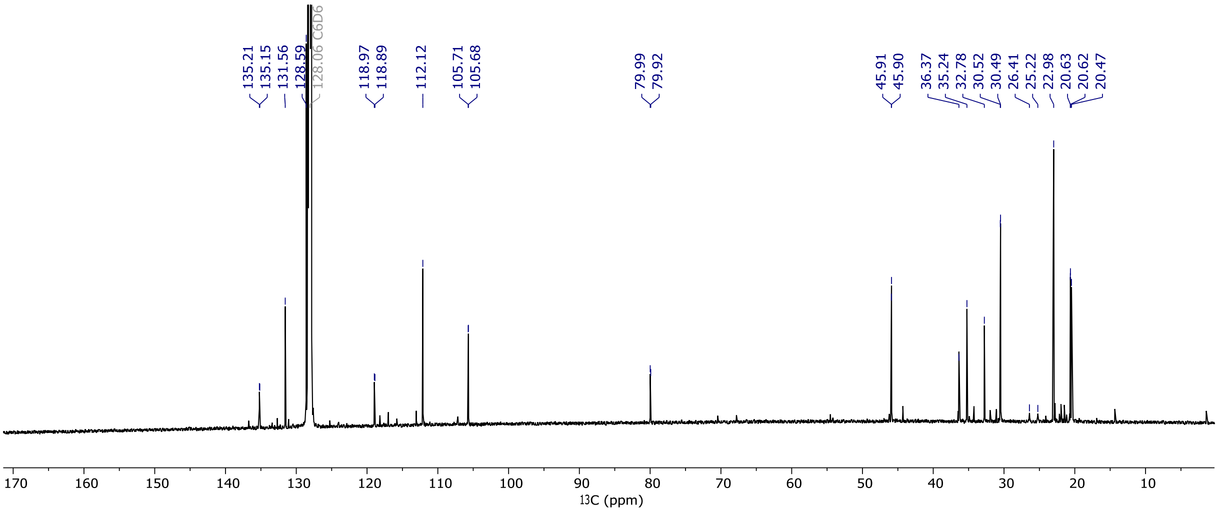

Figure S21: $^{13}C\{^1H\}$ NMR spectrum of **6** in C_6D_6.

1.2.8a Synthesis of 7

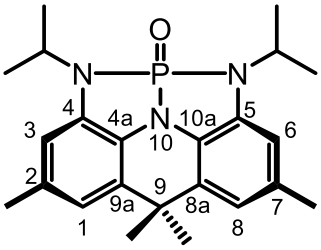

4^{iPr} (10.5 mg, 27.8 µmol, 1.00 eq.) and pyridine-N-oxide (2.6 mg, 27.8 µmol, 1.00 eq.) are dissolved in d_6-benzene (0.5 mL) in a J. Young NMR tube and stirred at room temperature for 2 hours. The solvent is removed *in vacuo* and the residue extracted into pentane (0.5 mL). Colourless crystals of **7** (6.3 mg, 15.9 µmol, 57%) are grown from a concentrated pentane solution at −30 °C.

^{1}H NMR (500 MHz, C_6D_6): δ = 6.56 (2H, s, $C_{1/8}H$), 6.32 (2H, s, $C_{3/6}H$), 3.98 (2H, d-sept, $^3J_{P–H}$ = 15.4 Hz, $^3J_{H–H}$ = 6.9 Hz, $C_{4/5}NCH(CH_3)_2$), 2.19 (6H, s, $C_{2/7}(CH_3)$), 1.59 (3H, s, $C_9(CH_3)$), 1.55 (3H, s, $C_9(CH_3)$), 1.48 (6H, d, $^3J_{H–H}$ = 6.8 Hz, $C_{4/5}NCH(CH_3)$), 1.35 ppm (6H, d, $^3J_{H–H}$ = 6.8 Hz, $C_{4/5}NCH(CH_3)$).

^{1}H{^{31}P} NMR (500 MHz, C_6D_6): as above, except δ = 3.98 ppm (2H, sept, $^3J_{H–H}$ = 6.9 Hz, $C_{4/5}NCH(CH_3)_2$).

^{13}C{^{1}H} NMR (151 MHz, C_6D_6): δ = 135.3 (s, C_{Ar}), 135.2 (d, $J_{C–P}$ = 9.68 Hz, C_{Ar}), 133.9 (s, $C_{2/7}$), 133.2 (d, $J_{C–P}$ = 8.8 Hz, C_{Ar}), 115.8 (s, $C_{1/8}$), 109.9 (d, $^3J_{C–P}$ = 10.5 Hz, $C_{3/6}$), 46.6 (d, $^2J_{C–P}$ = 3.5 Hz, $C_{4/5}NCH(CH_3)_2$), 40.2 (m, C_9), 26.4 (s, $C_9(CH_3)$), 22.6 (s, $C_9(CH_3)$), 22.3 (s, $C_{2/7}(CH_3)$), 21.4 (d, $^3J_{C–P}$ = 1.1 Hz, $C_{4/5}NCH(CH_3)$), 20.8 ppm (d, $^3J_{C–P}$ = 2.0 Hz, $C_{4/5}NCH(CH_3)$.

^{31}P NMR (202 MHz, C_6D_6): δ = 37.4 ppm (t, $^3J_{P–H}$ = 15.4 Hz).

^{31}P{^{1}H} NMR (162 MHz, C_6D_6): δ = 37.4 ppm (s).

Elemental analysis calcd. (%) for $C_{23}H_{30}N_3O_1P$: C 69.85, H 7.65, N 10.63; found: C 69.19, H 7.61, N 10.09.

1.2.8b Alternative route to 7

4^{iPr} (7.0 mg, 18.5 µmol, 1.00 eq.) is dissolved in tetrahydrofuran (0.5 mL) in a J. Young NMR tube. The solution is freeze-pump-thaw degassed four times then placed under an atmosphere of oxygen (1 atm., excess). The resulting dark red solution is heated at 60 °C for 16 hours. The solution is then cooled to room temperature and the solvent removed *in vacuo*. The residues are extracted with pentane (3 × 1 mL). Off-white microcrystalline material of **7** (4.0 mg, 10.1 µmol, 55%) is obtained from a concentrated pentane solution at –30 °C.

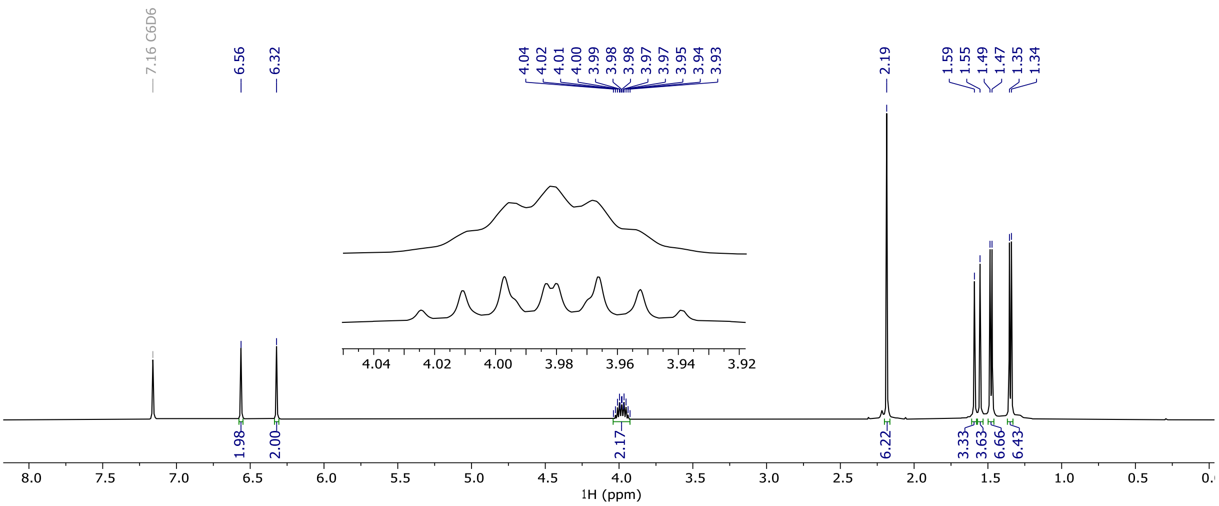

Figure S22: ^{1}H NMR spectrum of **7** in C_6D_6. Inset: top is $^{1}H\{^{31}P\}$ and bottom is ^{1}H NMR spectrum enhanced between 4.05 – 3.92 ppm.

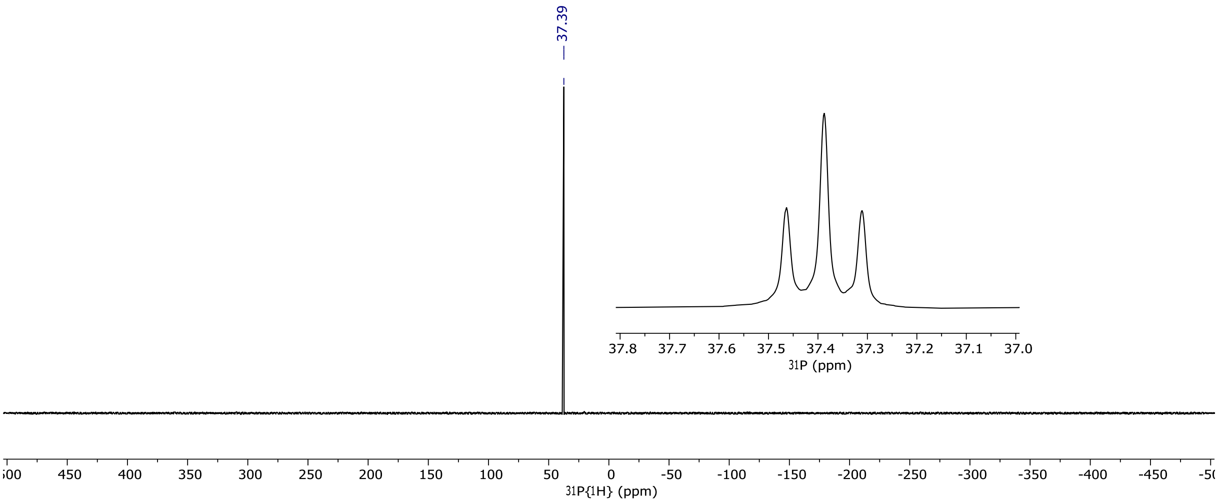

Figure S23: $^{31}P\{^{1}H\}$ NMR spectrum of **7** in C_6D_6. Inset: ^{31}P NMR spectrum enhanced between 37.80 – 37.00 ppm.

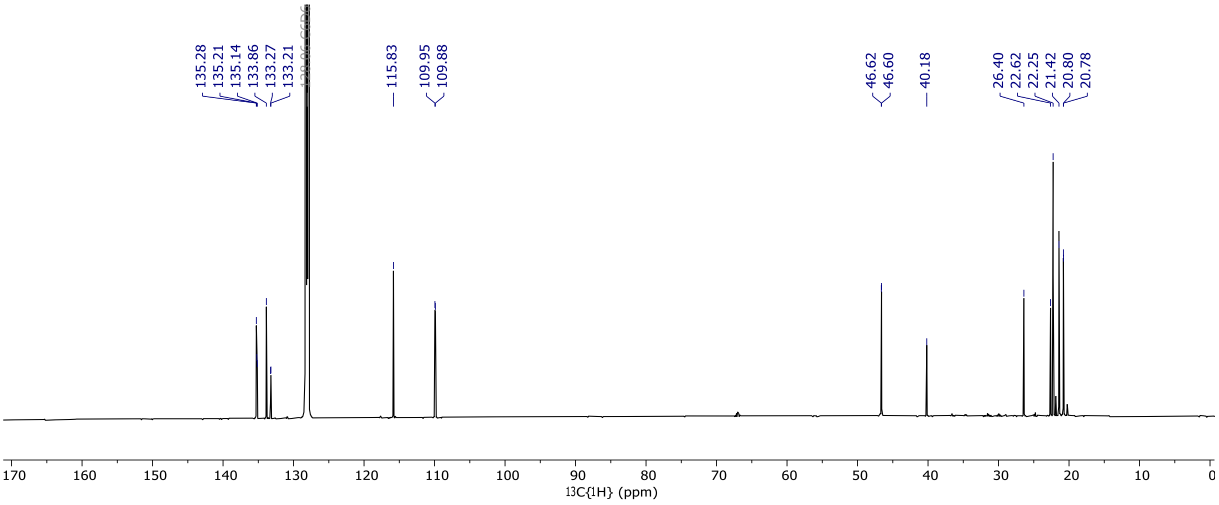

Figure S24: $^{13}C\{^{1}H\}$ NMR spectrum of **7** in C_6D_6.

1.2.9a Synthesis of 8

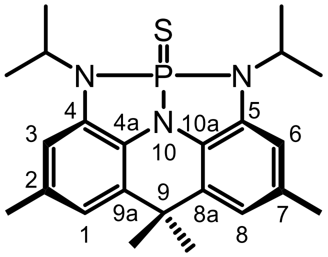

7 (6.0 mg, 15.2 µmol, 1.00 eq.) and 2,4-bis(4-methoxyphenyl-1,3,2,4-dithiadiphosphetane-2,4-disulfide (3.1 mg, 7.6 µmol, 0.50 eq.) are dissolved in toluene (0.5 mL) in a J. Young NMR tube and heated to 110 °C for 16 hours. Full conversion to **8** is observed by ^{31}P NMR spectroscopy. The solvent is removed *in vacuo* and the product extracted into hexane (0.5 mL). **8** was crystallised at −30 °C following concentration of this hexane solution by slow evaporation (6.0 mg, 14.6 µmol, 96%).

^{1}H NMR (500 MHz, C_6D_6): δ = 6.55 (2H, s, $C_{1/8}H$), 6.34 (2H, s, $C_{3/6}H$), 4.26 (2H, d-sept, $^{3}J_{P–H}$ = 15.0 Hz, $^{3}J_{H–H}$ = 7.1 Hz, $C_{4/5}NCH(CH_3)_2$), 2.17 (6H, s, $C_{2/7}CH_3$), 1.58 (3H, s, $C_9(CH_3)$), 1.56 (3H, s, $C_9(CH_3)$), 1.51 (6H, d, $^{3}J_{H–H}$ = 7.0 Hz, $C_{4/5}NCH(CH_3)$), 1.29 ppm (6H, d, $^{3}J_{H–H}$ = 7.0 Hz, $C_{4/5}NCH(CH_3)$).

^{1}H{^{31}P} NMR (500 MHz, C_6D_6): as above, except δ = 4.26 ppm (2H, sept, $^{3}J_{H–H}$ = 7.0 Hz, $C_{4/5}NCH(CH_3)_2$).

^{13}C{^{1}H} NMR (151 MHz, C_6D_6): δ = 135.4 (d, $^{3}J_{C–P}$ = 10.6 Hz, $C_{8a/9a}$), 134.5 (s, C_{Ar}), 134.4 (d, $J_{C–P}$ = 12.6 Hz, C_{Ar}), 133.8 (s, $C_{2/7}$), 116.1 (s, $C_{1/8}$) 109.9 (s, $C_{3/6}$), 48.4 (d, $^{2}J_{C–P}$ = 5.9 Hz, $C_{4/5}NCH(CH_3)_2$), 40.2 (s, C_9), 26.2 (s, $C_9(CH_3)$), 22.7 (s, $C_9(CH_3)$), 22.2 (s, $C_{2/7}(CH_3)$), 20.8 (d, $^{3}J_{C–P}$ = 1.6 Hz, $C_{4/5}NCH(CH_3)$), 20.1 ppm (d, $^{3}J_{C–P}$ = 3.5 Hz, $C_{4/5}NCH(CH_3)$).

^{31}P NMR (202 MHz, C_6D_6): δ = 83.8 ppm (t, $^{3}J_{P–H}$ = 15.0 Hz).

^{31}P{^{1}H} NMR (162 MHz, C_6D_6): δ = 83.8 ppm (s).

HRMS (ESI) *m/z* calcd. for $C_{23}H_{30}N_3PS+H^+$: 412.1971 [*M*+H]$^+$; found: 412.1964.

1.2.9b Alternative route to 8

4^{iPr} (15.0 mg, 39.5 µmol, 1.00 eq.) and S_8 (6.4 mg, 0.198 mmol, 5.00 eq.) are dissolved in *d*$_2$-dichloromethane (0.5 mL) in a J. Young NMR tube and stirred for 4 hours. Full conversion to thiophosphorane **8** is observed by ^{31}P{^{1}H} and ^{1}H NMR,* however isolation of this product is hindered by co-crystallisation of sulfur.

* See **Section 2.5** for ^{31}P{^{1}H} and ^{1}H NMR spectra of this reaction mixture.

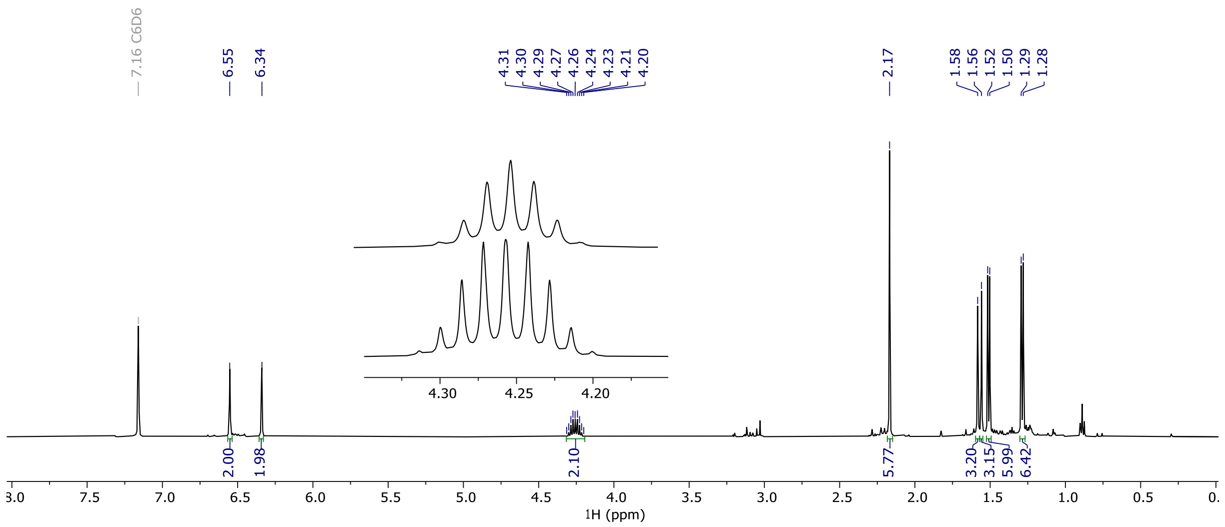

Figure S25: ^{1}H NMR spectrum of **8** in C_6D_6. Inset: top is ^{1}H{^{31}P} and bottom is ^{1}H NMR spectrum enhanced between 4.35 – 3.92 ppm.

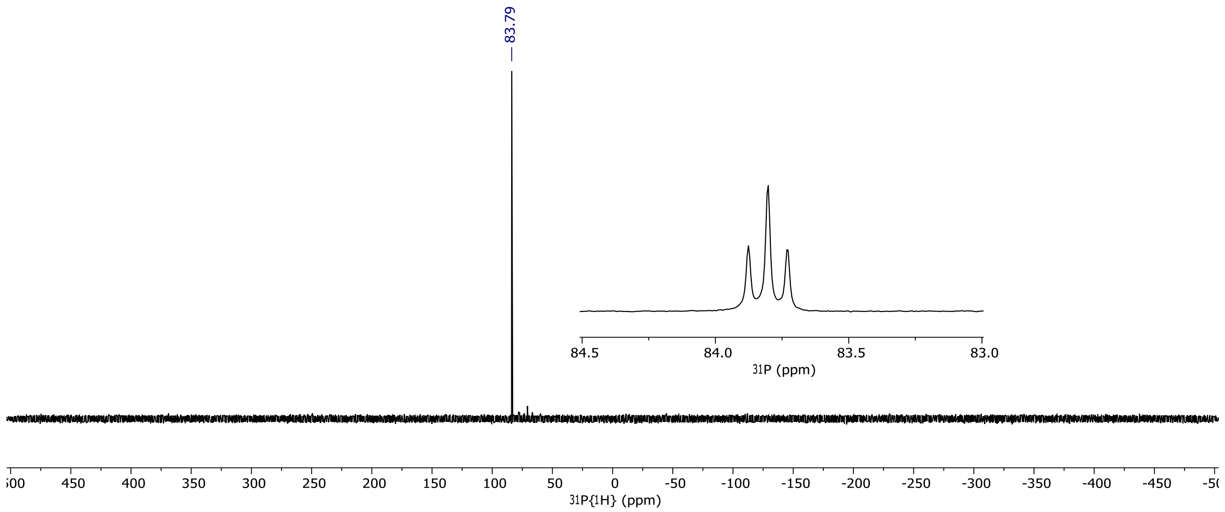

Figure S26: ^{31}P{^{1}H} NMR spectrum of **8** in C_6D_6. Inset: ^{31}P NMR spectrum enhanced between 84.50 – 83.00 ppm.

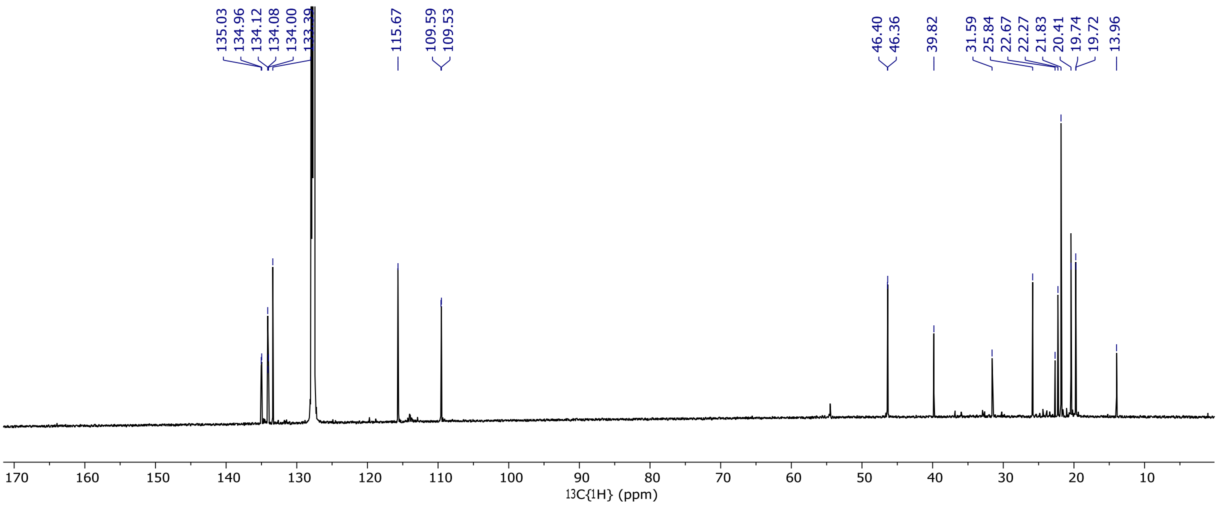

Figure S27: $^{13}C\{^{1}H\}$ NMR spectrum of **8** in C_6D_6.

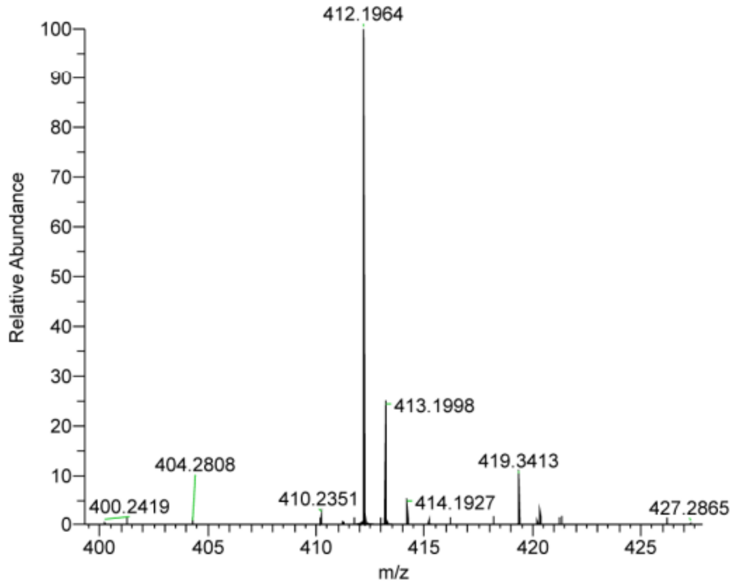

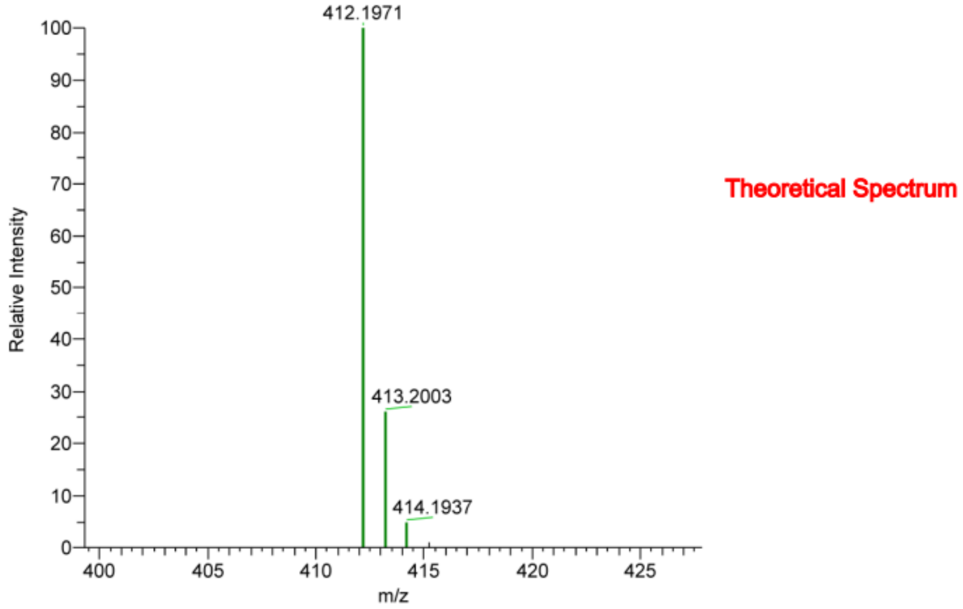

Figure S28: Top: high-resolution mass spectrum of **8** showing the [*M*+H]$^+$ ion at *m/z* 412.1964. Bottom: theoretical spectrum.

1.2.10 Synthesis of 9

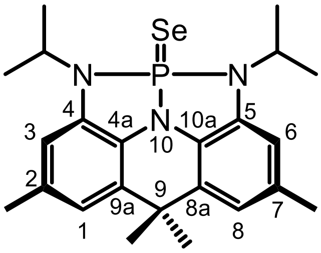

4^{iPr} (10.0 mg, 26.4 µmol, 1.00 eq.) and selenium powder (10.4 mg, 0.132 mmol, 5.00 eq.) are suspended in d_2-dichloromethane (0.5 mL) in a J. Young NMR tube and stirred for 2.5 hours at room temperature. Full conversion to **9** was observed by ^{31}P NMR spectroscopy. The solvent is removed *in vacuo* and the residue is washed with hexane (0.5 mL). The product is extracted into tetrahydrofuran (0.3 mL), filtered, and crystallised by pentane vapour diffusion (2 mL). **9** is isolated as yellow crystals after one week (6.5 mg, 14.2 µmol, 54%).

^{1}H NMR (500 MHz, C_6D_6): δ = 6.55 (2H, s, $C_{1/8}H$), 6.36 (2H, s, $C_{3/6}H$), 4.43 (2H, d-sept, $^{3}J_{P-H}$ = 14.6 Hz, $^{3}J_{H-H}$ = 6.9 Hz, $C_{4/5}NCH(CH_3)_2$), 2.16 (6H, s, $C_{2/7}CH_3$), 1.58 (3H, s, $C_9(CH_3)$), 1.55 (3H, s, $C_9(CH_3)$), 1.52 (6H, d, $^{3}J_{H-H}$ = 6.9 Hz, $C_{4/5}NCH(CH_3)$), 1.27 ppm (6H, d, $^{3}J_{H-H}$ = 7.0 Hz, $C_{4/5}NCH(CH_3)$).

^{1}H{^{31}P} NMR (500 MHz, C_6D_6): as above, except δ = 4.43 ppm (2H, sept, $^{2}J_{H-H}$ = 7.0 Hz, $C_{4/5}NCH(CH_3)_2$).

^{13}C{^{1}H} NMR (151 MHz, C_6D_6): δ = 135.4 (d, $^{3}J_{C-P}$ = 10.4 Hz, $C_{9a/8a}$), 135.0 (d, $^{2}J_{C-P}$ = 3.0 Hz, $C_{4a/10a}$), 134.0 (d, $^{2}J_{C-P}$ = 14.9 Hz, $C_{4/5}$), 133.8 (s, $C_{2/7}$), 116.2 (s, $C_{1/8}$), 110.1 (d, $^{3}J_{C-P}$ = 8.6 Hz, $C_{3/6}$), 47.1 (d, $^{2}J_{C-P}$ = 7.4 Hz, $C_{4/5}NCH(CH_3)$), 40.2 (s, C_9), 26.3 (s, $C_9(CH_3)$), 22.6 (s, $C_9(CH_3)$), 22.2 (s, $C_{2/7}(CH_3)$), 20.7 (d, $^{3}J_{C-P}$ = 1.7 Hz, $C_{4/5}NCH(CH_3)$), 20.0 ppm (d, $^{3}J_{C-P}$ = 3.9 Hz, $C_{4/5}NCH(CH_3)$).

^{31}P NMR (243 MHz, C_6D_6): δ = 75.7 ppm (dt, $^{1}J_{P-77Se}$ = 919.2 Hz, $^{3}J_{P-H}$ = 14.7 Hz).

^{31}P{^{1}H} NMR (162 MHz, C_6D_6): δ = 75.6 ppm (dt, $^{1}J_{P-77Se}$ = 918.8 Hz).

^{77}Se NMR (95 MHz, C_6D_6): δ = −35.4 ppm (d, $^{1}J_{P-77Se}$ = 916.8 Hz).

Elemental analysis calcd. (%) for $C_{23}H_{30}N_3PSe$: C 60.26, H 6.60, N 9.17; found: C 60.05, H 6.61, N 9.05.

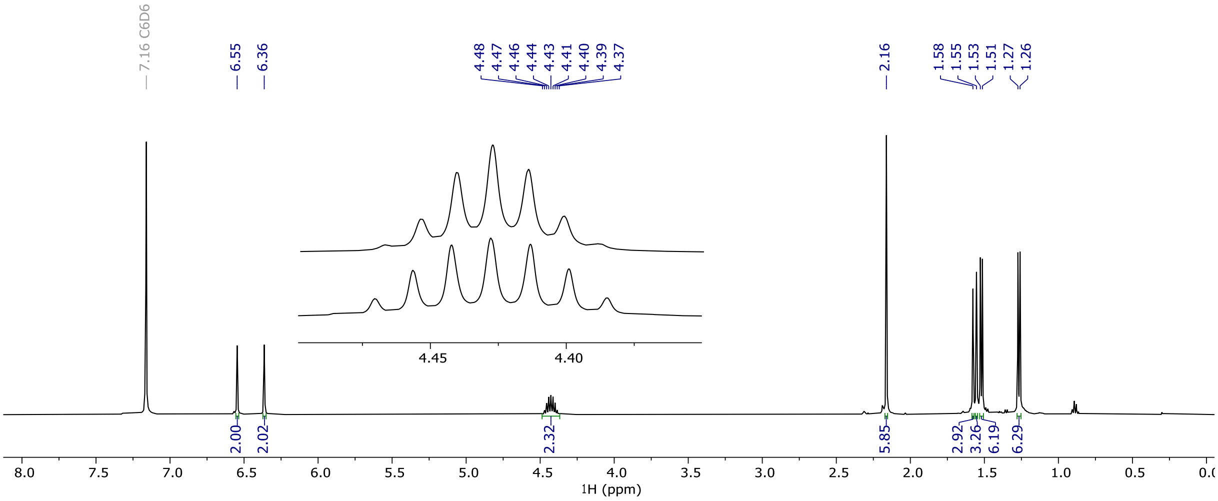

Figure S29: ^{1}H NMR spectrum of **9** in C_6D_6. Inset: top is $^{1}H\{^{31}P\}$ and bottom is ^{1}H NMR spectrum enhanced between 4.50 – 4.45 ppm.

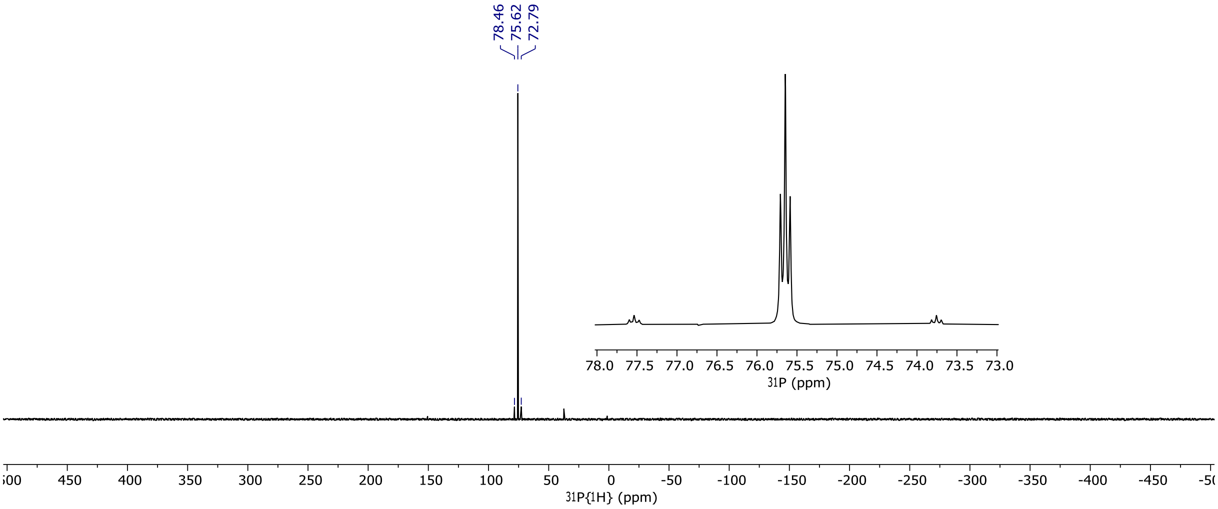

Figure S30: $^{31}P\{^{1}H\}$ NMR spectrum of **9** in C_6D_6. Inset: ^{31}P NMR spectrum enhanced between 78.00 – 73.00 ppm.

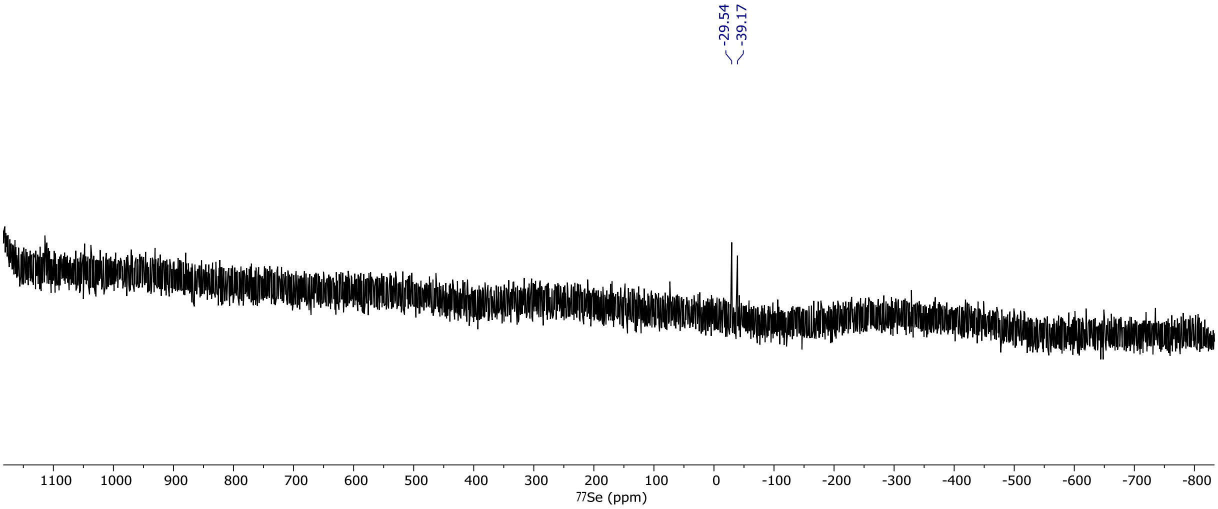

Figure S31: ^{77}Se NMR spectrum of **9** in C_6D_6.

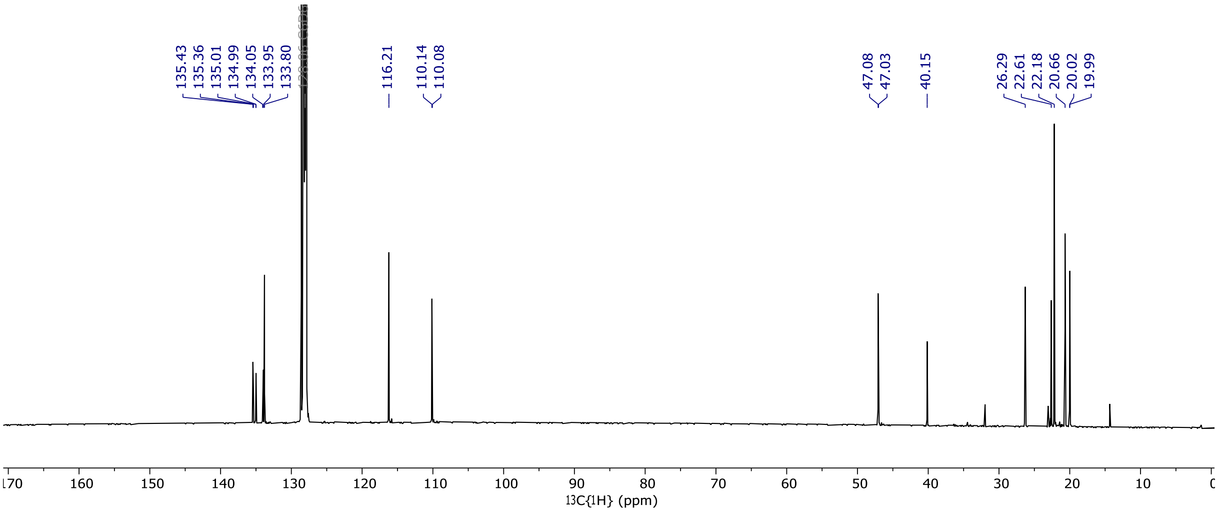

Figure S32: $^{13}C\{^{1}H\}$ NMR spectrum of **9** in C_6D_6.

1.2.11 Synthesis of 10

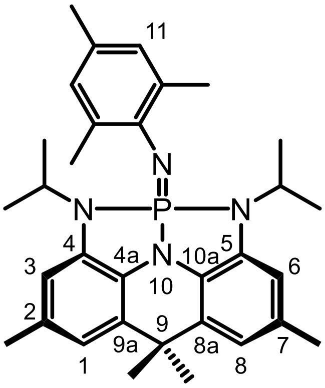

4^{iPr} (10.5 mg, 27.8 µmol, 1.00 eq.) is dissolved in a toluene (0.5 mL) solution of mesityl-azide (4.5 mg, 27.8 µmol, 1.00 eq.) in a J. Young NMR tube. The solution was heated at 110 °C for 2 hours generating a colourless solution. The solvent is removed *in vacuo* and the residues heated under dynamic vacuum for 30 minutes to remove any unreacted mesityl-azide. The residue is dissolved in hexane (0.5 mL) and crystals of **10** (10.0 mg, 19.5 µmol, 70%) were grown by slow evaporation of this hexane solution.

^{1}H NMR (500 MHz, C_6D_6): δ = 6.92 (2H, s, $C_{11}H$), 6.61 (2H, s, $C_{1/8}H$), 6.44 (2H, s, $C_{3/6}H$), 4.13 (2H, d-sept, $^{3}J_{P–H}$ = 11.7 Hz, $^{3}J_{H–H}$ = 7.0 Hz, $C_{4/5}NCH(CH_3)_2$), 2.50 (6H, br s, $C_{Mes}(CH_3)$), 2.26 (3H, d, $J_{P–H}$ = 2.8 Hz, $C_{Mes}(CH_3)$), 2.21 (6H, s, $C_{2/7}(CH_3)$, 1.66 (3H, s, $C_9(CH_3)$), 1.64 (3H, s, $C_9(CH_3)$), 1.30 (6H, d, $^{3}J_{H–H}$ = 6.9 Hz, $C_{4/5}NCH(CH_3)$), 1.17 ppm (6H, d, $^{3}J_{H–H}$ = 7.0 Hz, $C_{4/5}NCH(CH_3)$).

^{1}H{^{31}P} NMR (500 MHz, C_6D_6): as above, except δ = 4.13 (2H, sept, $^{3}J_{H–H}$ = 7.0 Hz, $C_{4/5}NCH(CH_3)_2$), 2.26 ppm (3H, s, $C_{Mes}(CH_3)$).

^{13}C{^{1}H} NMR (151 MHz, C_6D_6): δ = 143.1 (d, $J_{C–P}$ = 3.4 Hz, C_{Mes}), 134.4 (d, $^{3}J_{C–P}$ = 10.0 Hz, $C_{8a/9a}$), 133.8 (d, $^{2}J_{C–P}$ = 19.6 Hz, $C_{4/5}$), 133.5 (d, $^{2}J_{C–P}$ = 7.3 Hz, $C_{4a/10a}$), 133.5 (s, $C_{2/7}$), 131.3 (m, C_{Mes}), 129.5 (m, C_{11}), 129.0 (d, $J_{C–P}$ = 4.1 Hz, C_{Mes}), 115.6 (s, $C_{1/8}$), 110.3 (d, $^{3}J_{C–P}$ = 10.4 Hz, $C_{3/6}$), 47.0 (d, $^{2}J_{C–P}$ = 2.6 Hz, $C_{4/5}NCH(CH_3)_2$), 40.0 (s, C_9), 26.8 (s, $C_9(CH_3)$), 22.5 (s, $C_9(CH_3)$), 22.3 (s, $C_{2/7}(CH_3)$), 20.95 – 20.88 (overlapping signals corresponding to one $C_{Mes}(CH_3)$ environment, and one set of $C_{4/5}NCH(CH_3)$ environments), 20.7 (s, $C_{Mes}(CH_3)$), 20.0 ppm (d, $^{3}J_{C–P}$ = 4.4 Hz, $C_{4/5}NCH(CH_3)$).

^{31}P NMR (243 MHz, C_6D_6): δ = 18.1 ppm (m).

^{31}P{^{1}H} NMR (243 MHz, C_6D_6): δ = 18.1 ppm (s).

Elemental analysis calcd (%) for $C_{32}H_{41}N_4P$: C 74.97, H 8.06, N 10.93; found: C 74.91, H 8.30, N 10.54.

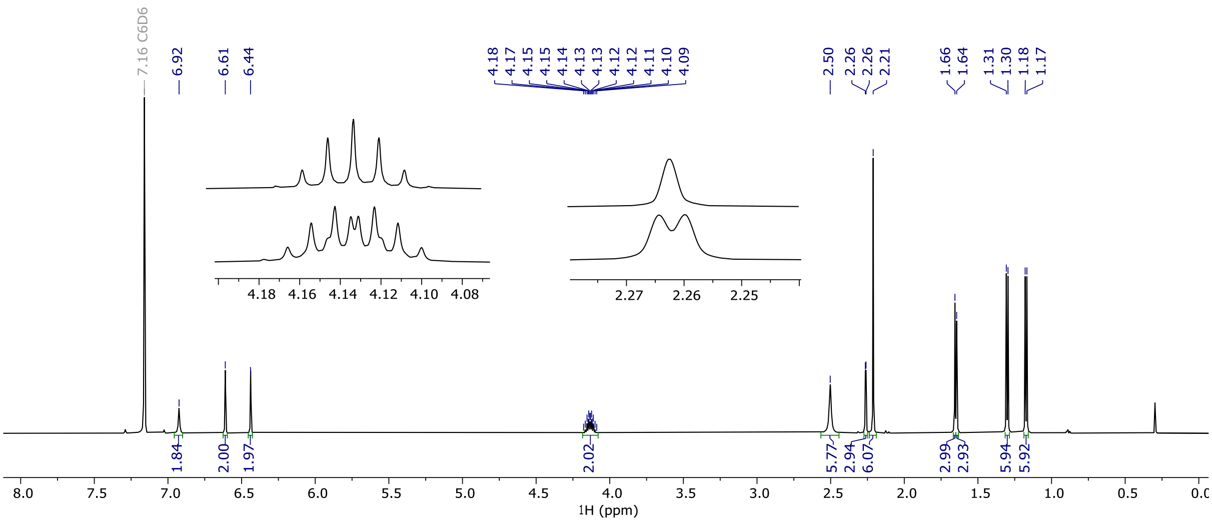

Figure S33: ^{1}H NMR spectrum of **10** in C_6D_6. Left inset: top is $^{1}H\{^{31}P\}$ and bottom is ^{1}H NMR spectrum enhanced between 4.20 – 4.07 ppm. Right inset: top is $^{1}H\{^{31}P\}$ and bottom is ^{1}H NMR spectrum enhanced between 2.28 – 2.24 ppm.

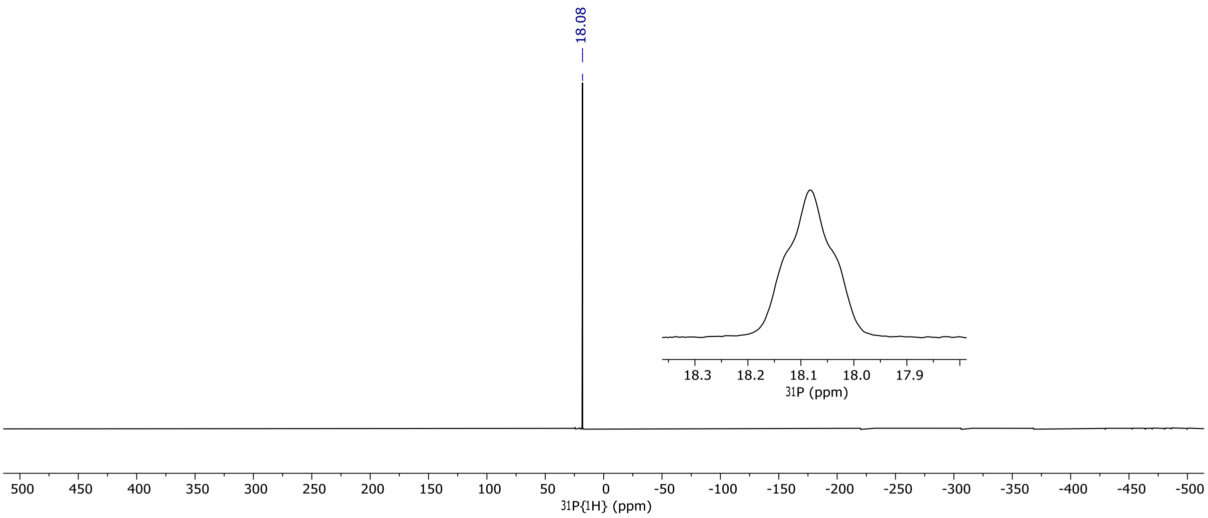

Figure S34: $^{31}P\{^{1}H\}$ NMR spectrum of **10** in C_6D_6. Inset: ^{31}P NMR spectrum enhanced between 18.36 – 17.80 ppm.

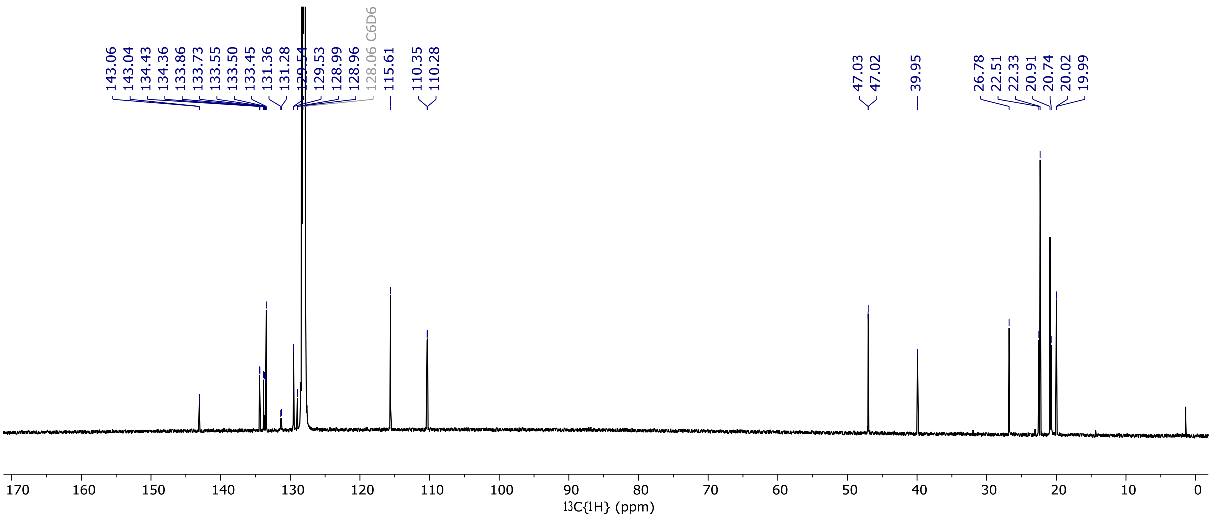

Figure S35: $^{13}C\{^{1}H\}$ NMR spectrum of **10** in C_6D_6.

1.2.12 Synthesis of 11

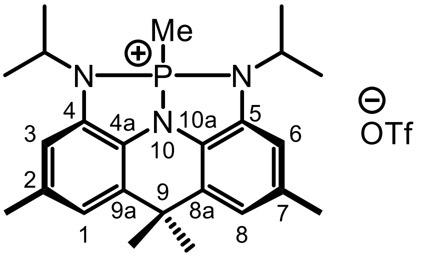

4^{iPr} (10.0 mg, 26.4 µmol, 1.00 eq.) is dissolved in d$_6$-benzene (0.5 mL) in a J. Young NMR tube. Methyl triflate (2.9 µL, 26.4 µmol, 1.0 eq.) is added and the solution heated to 80 °C for 3 hours. Yellow needle-like crystals form over the course of the reaction. The mixture is cooled gradually to room temperature and left for 16 hours. The crystals are filtered, washed with benzene (3 × 0.5 mL) and dried *in vacuo* to yield **11** (7.5 mg, 13.8 µmol, 52%).

^{1}H NMR (500 MHz, CD_2Cl_2): δ = 6.88 (2H, s, $C_{1/8}$*H*), 6.81 (2H, s, $C_{3/6}$*H*), 4.55 (2H, d-sept, $^{3}J_{P–H}$ = 11.6 Hz, $^{3}J_{H–H}$ = 6.9 Hz, $C_{4/5}$NC*H*(CH_3)$_2$), 3.13 (3H, d, $^{2}J_{P–H}$ = 15.6 Hz, P–C*H*$_3$), 2.38 (6H, s, $C_{2/7}$(C*H*$_3$)), 1.82 (3H, s, C_9(C*H*$_3$)), 1.70 (6H, d, $^{3}J_{H–H}$ = 7.0 Hz, $C_{4/5}$NCH(C*H*$_3$)), 1.64 (3H, s, C_9(C*H*$_3$)), 1.58 ppm (6H, d, $^{3}J_{H–H}$ = 6.8 Hz, $C_{4/5}$NCH(C*H*$_3$)).

^{1}H{^{31}P} NMR (500 MHz, CD_2Cl_2): as above, except δ = 4.55 (2H, sept, $^{3}J_{H–H}$ = 6.8 Hz, $C_{4/5}$NC*H*(CH_3)$_2$), 3.13 ppm (3H, s, P–C*H*$_3$).

^{13}C{^{1}H} NMR (151 MHz, CD_2Cl_2): δ = 137.0 (s, *C*$_{4a/10a}$), 136.4 (d, $^{3}J_{C–P}$ = 10.4 Hz, *C*$_{8a/9a}$), 133.0 (d, $^{4}J_{C–P}$ = 3.0 Hz, *C*$_{2/7}$), 131.4 (d, $^{2}J_{C–P}$ = 15.7 Hz, *C*$_{4/5}$), 119.4 (d, $^{4}J_{C–P}$ = 1.8 Hz, *C*$_{1/8}$), 113.0 (d, $^{3}J_{C–P}$ = 9.1 Hz, *C*$_{3/6}$), 47.9 (d, $^{2}J_{C–P}$ = 4.1 Hz, $C_{4/5}$N*C*H(CH_3)$_2$), 40.5 (s, *C*$_9$), 26.9 (s, C_9(*C*H$_3$)), 22.5 (s, C_9(*C*H$_3$)), 22.2 (s, $C_{2/7}$(*C*H$_3$)), 21.6 (s, $C_{4/5}$NCH(*C*H$_3$)), 21.1 (d, $^{3}J_{C–P}$ = 5.8 Hz, $C_{4/5}$NCH(*C*H$_3$)), 16.0 ppm (d, $^{1}J_{C–P}$ = 125.1 Hz, P–*C*H$_3$).

^{31}P NMR (243 MHz, CD_2Cl_2): δ = 80.5 ppm (qt, $^{2}J_{P–H}$ = 15.4 Hz, $^{3}J_{P–H}$ = 11.6 Hz).

^{31}P{^{1}H} NMR (243 MHz, CD_2Cl_2): δ = 80.5 ppm (s).

^{19}F{^{1}H} NMR (470 MHz, CD_2Cl_2): δ = 78.9 ppm (s, S(O)$_3$(C*F*$_3$)$^-$).

Elemental analysis calcd. (%) for $C_{25}H_{33}F_3N_3O_3PS$: C 55.24, H 6.12, N 7.73; found: C 55.81, H 6.07, N 7.59.

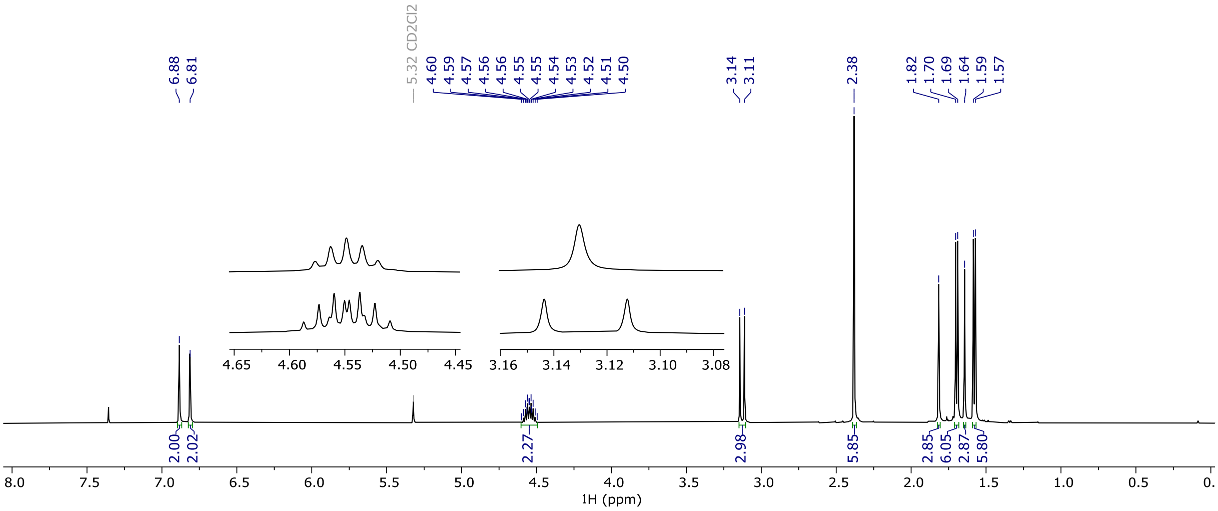

Figure S36: ^{1}H NMR spectrum of **11** in CD_2Cl_2. Left inset: top is $^{1}H\{^{31}P\}$ and bottom is ^{1}H NMR spectrum enhanced between 4.65 – 4.45 ppm. Right inset: top is $^{1}H\{^{31}P\}$ and bottom is ^{1}H NMR spectrum enhanced between 3.16 – 3.08 ppm.

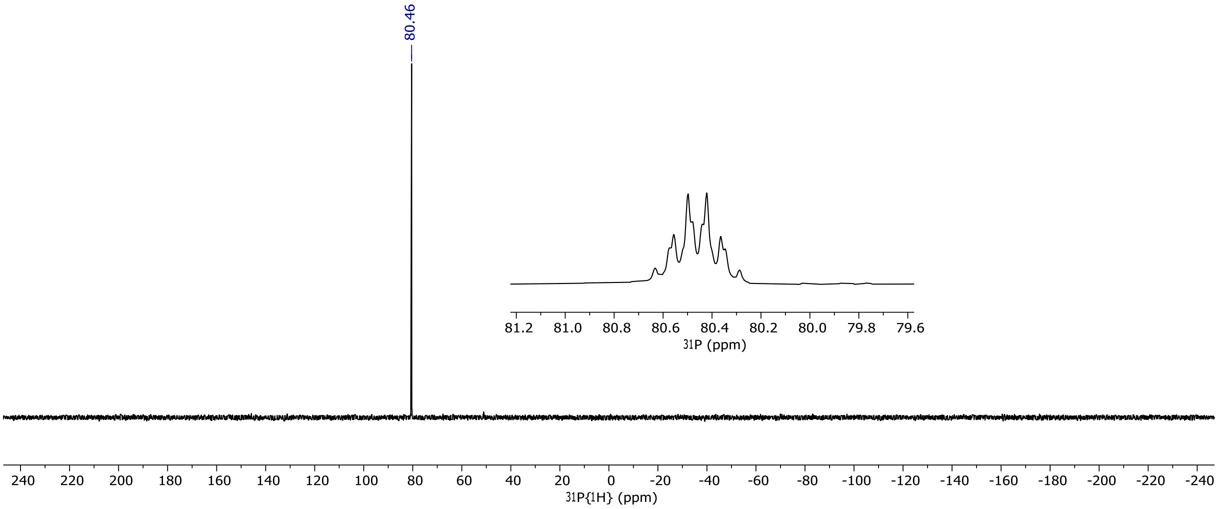

Figure S37: $^{31}P\{^{1}H\}$ NMR spectrum of **11** in CD_2Cl_2. Inset: ^{31}P NMR spectrum enhanced between 81.20 – 79.60 ppm.

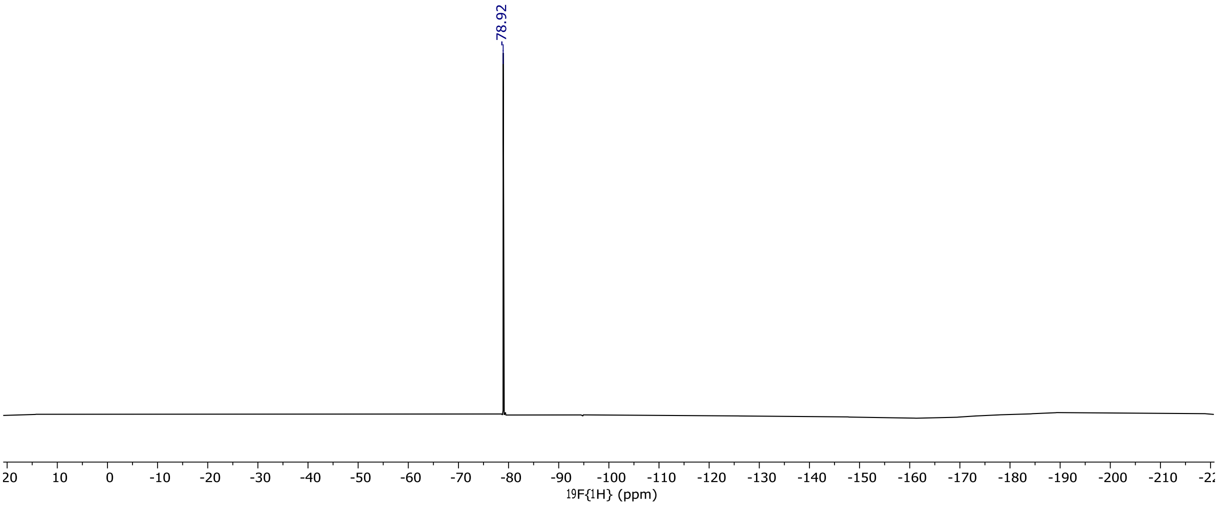

Figure S38: $^{19}F\{^{1}H\}$ NMR spectrum of **11** in CD_2Cl_2.

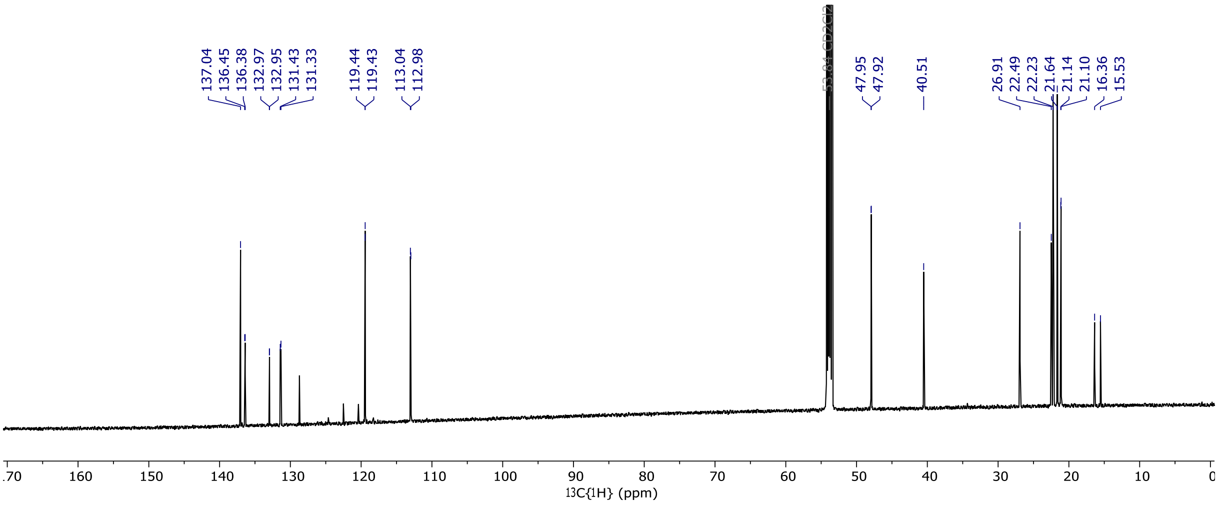

Figure S39: $^{13}C\{^{1}H\}$ NMR spectrum of **11** in CD_2Cl_2.

1.2.13 Synthesis of 12

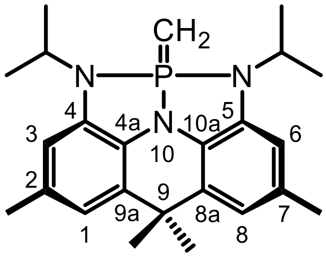

4^{iPr} (10.5 mg, 27.8 µmol, 1.00 eq.) is dissolved in d_6-benzene (0.5 mL) in a J. Young NMR tube. Methyl triflate (3.0 µL, 27.8 µmol, 1.00 eq.) is added and the solution heated to 80 °C for 3 hours. The solvent is removed *in vacuo* and the residues dissolved in a solution of potassium *tert*-butoxide (3.1 mg, 27.8 µmol, 1.00 eq.) in tetrahydrofuran (0.5 mL). A colourless solution results. The solvent is removed *in vacuo* and the product extracted into hexane (2 × 0.5 mL). Colourless crystals of **12** (7.6 mg, 19.3 µmol, 69%) were grown from a concentrated hexane solution at −30 °C.

^{1}H NMR (600 MHz, C_6D_6): δ = 6.63 (2H, s, $C_{1/8}H$), 6.47 (2H, s, $C_{3/6}H$), 4.23 (2H, d-sept, $^3J_{P–H}$ = 11.4 Hz, $^3J_{H–H}$ = 7.0 Hz, $C_{4/5}NCH(CH_3)_2$), 2.26 (6H, s, $C_{2/7}(CH_3)$), 1.65 (3H, s, $C_9(CH_3)$), 1.59 (3H, s, $C_9(CH_3)$), 1.40 (6H, d, $^3J_{H–H}$ = 7.0 Hz, $C_{4/5}NCH(CH_3)$), 1.20 (2H, d, $^2J_{P–H}$ = 18.5 Hz, P–(CH_2)), 1.19 ppm (6H, d, $^3J_{H–H}$ = 7.1 Hz, $C_{4/5}NCH(CH_3)$).

^{1}H{^{31}P} NMR (600 MHz, C_6D_6): as above, except δ = 4.23 (2H, sept, $^3J_{H–H}$ = 7.0 Hz, $C_{4/5}NCH(CH_3)_2$), 1.20 ppm (2H, s, P–(CH_2)).

^{13}C{^{1}H} NMR (151 MHz, C_6D_6): δ = 133.8 (d, $^2J_{C–P}$ = 4.1 Hz, $C_{4a/10a}$), 133.1 (d, $^2J_{C–P}$ = 16.4 Hz, $C_{4/5}$), 132.9 (d, $^3J_{C–P}$ = 10.3 Hz, $C_{8a/9a}$), 132.4 (s, $C_{2/7}$), 115.6 (s, $C_{1/8}$), 109.8 (d, $^3J_{C–P}$ = 8.9 Hz, $C_{3/6}$), 47.1 (d, $^2J_{C–P}$ = 3.9 Hz, $C_{4/5}NCH(CH_3)_2$), 39.4 (s, C_9), 27.4 (s, $C_9(CH_3)$), 22.8 (s, $C_9(CH_3)$), 22.4 (s, $C_{2/7}(CH_3)$), 20.9 (s, $C_{4/5}NCH(CH_3)$), 20.5 (d, $^3J_{C–P}$ = 5.2 Hz, $C_{4/5}NCH(CH_3)$), 16.9 ppm (d, $^1J_{C–P}$ = 240.1 Hz, P–(CH_2)).

^{31}P NMR (243 MHz, C_6D_6): δ = 61.2 ppm (m).

^{31}P{^{1}H} NMR (162 MHz, C_6D_6): δ = 61.2 ppm (s).

Elemental analysis calcd. (%) for $C_{24}H_{32}N_3P$: C 73.25, H 8.20, N 10.68; found: C 73.63, H 8.40, N 10.16.

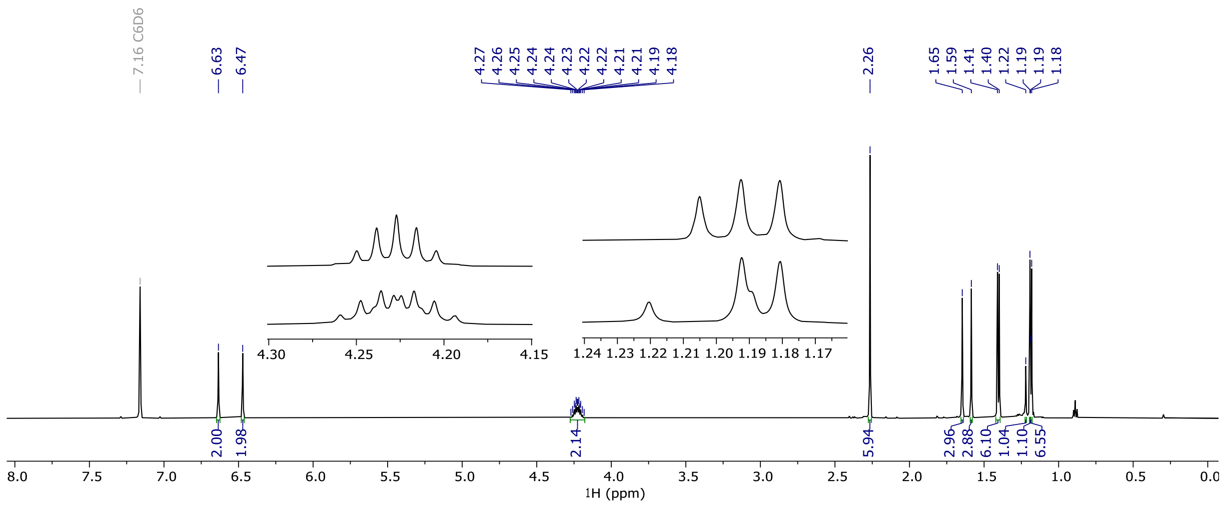

Figure S40: ^{1}H NMR spectrum of **12** in C_6D_6. Left inset: top is $^{1}H\{^{31}P\}$ and bottom is ^{1}H NMR spectrum enhanced between 4.30 – 4.15 ppm. Right inset: top is $^{1}H\{^{31}P\}$ and bottom is ^{1}H NMR spectrum enhanced between 1.24 – 1.26 ppm.

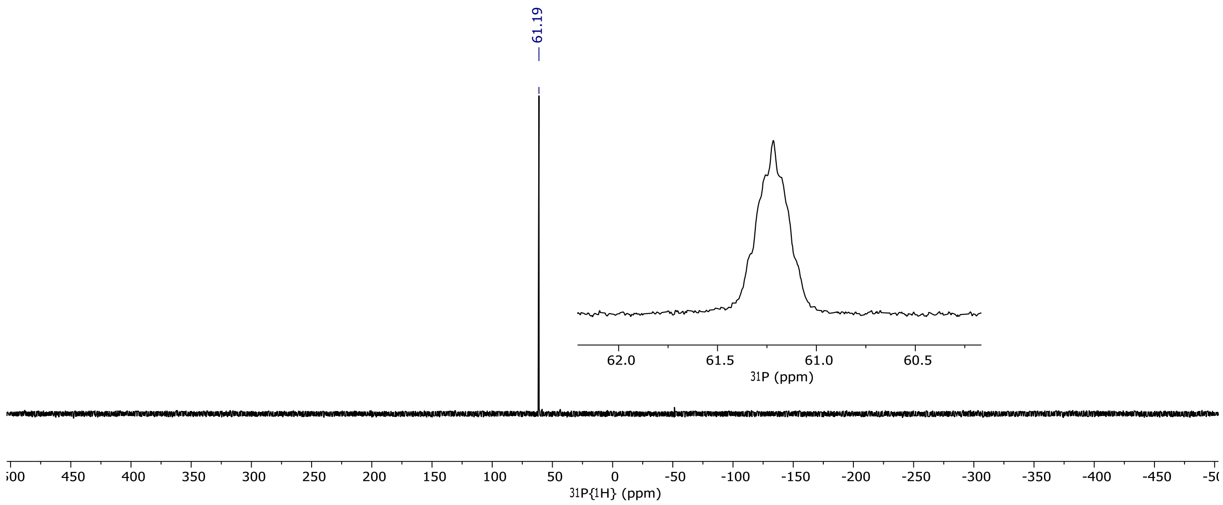

Figure S41: $^{31}P\{^{1}H\}$ NMR spectrum of **12** in C_6D_6. Inset: ^{31}P NMR spectrum enhanced between 62.25 – 60.25 ppm.

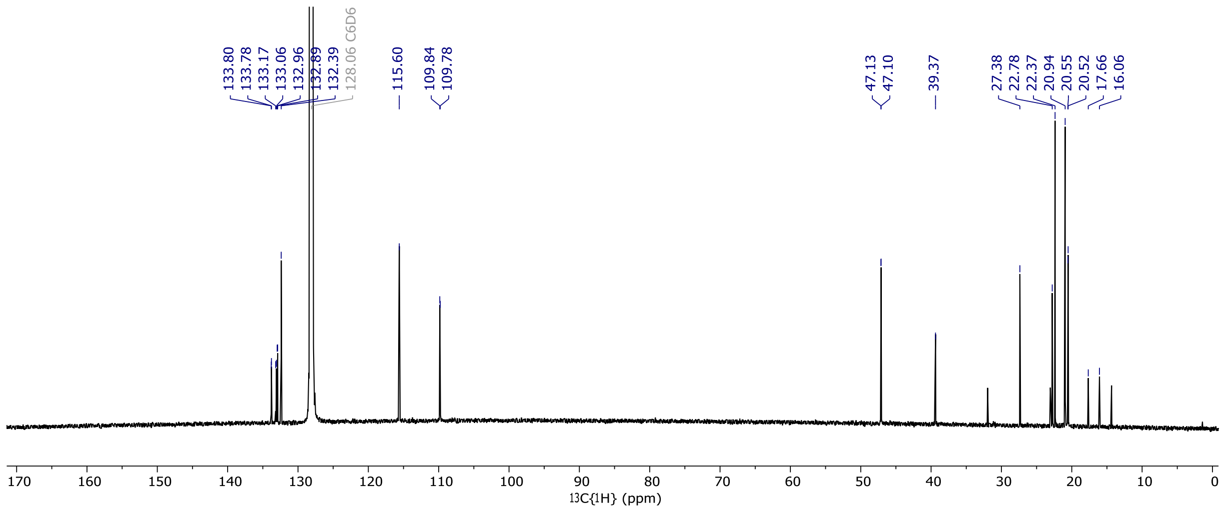

Figure S42: $^{13}C\{^1H\}$ NMR spectrum of **12** in CD_2Cl_2.

1.2.14 Synthesis of 13

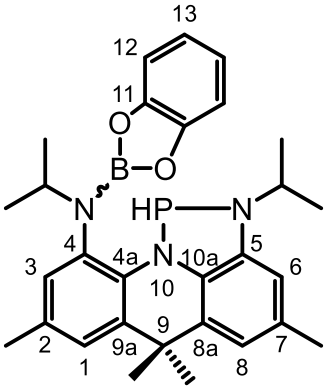

4^{iPr} (10.0 mg, 26.4 µmol, 1.00 eq.) is dissolved in d_6-benzene (0.5 mL) in a J. Young NMR tube. 2*H*-1,3,2-benzodioxaborole (2.8 µL, 26.4 µmol, 1.00 eq.) is added, resulting in the immediate generation of a colourless solution. Full conversion to an isomeric mixture of **13** (1:0.6 ratio) was observed by ^{31}P NMR spectroscopy. The solvent is removed *in vacuo*, dissolved in benzene and lyophilised to yield the isomeric mixture of **13** as an off white powder (9.6 mg, 19.2 µmol, 69%).*

*This provided a compositionally impure sample of **13** as described in **Section 2.7**. Therefore, the following characterisation was performed on a sample of **13** synthesised as above without the lyophilisation step (i.e. redissolved following initial solvent/excess starting material removal *in vacuo*).

^{1}H NMR (600 MHz, C_6D_6): ***major isomer*** δ = 7.20 (1H, s, C_1H), 7.02 (1H, d, $^1J_{P–H}$ = 135.0 Hz, P–H), 6.91 ($C_{13}H$)*, 6.86 (1H, m, C_3H), 6.70 (1H, s, C_8H), 6.68 ($C_{12}H$)*, 6.27 (1H, s, C_6H), 4.35 (1H, sept, $^2J_{H–H}$ = 6.7 Hz, $C_4NCH(CH_3)_2$), 3.54 (1H, d-sept, $^3J_{P–H}$ = 11.6 Hz, $^3J_{H–H}$ = 6.6 Hz, $C_5NCH(CH_3)_2$), 2.30 (3H, s, $C_7(CH_3)$), 2.18 (3H, s, $C_2(CH_3)$), 1.71 (3H, s, $C_9(CH_3)$), 1.51 (3H, s, $C_9(CH_3)$), 1.43 (3H, d, $^2J_{H–H}$ = 6.6 Hz, $C_4NCH(CH_3)$), 1.18 (3H, d, $^2J_{H–H}$ = 6.8 Hz, $C_5NCH(CH_3)$), 1.18 (3H, d, $^2J_{H–H}$ = 6.6 Hz, $C_4NCH(CH_3)$), 0.94 ppm (3H, d, $^2J_{H–H}$ = 6.5 Hz, $C_5NCH(CH_3)$). ***minor isomer*** δ = 7.16 (C_1H)*, 7.15 (1H, d, $^1J_{P–H}$ = 148.0 Hz, P–H), 6.98 (2H, br m, $C_{13}H$), 6.91 (C_3H)*, 6.74 (2H, dd, $^3J_{H–H}$ = 5.7 Hz, $^4J_{H–H}$ = 3.3 Hz, $C_{12}H$), 6.66 (1H, s, C_8H), 6.25 (s, 1H, C_6H), 4.11 (1H, sept, $^2J_{H–H}$ = 6.7 Hz, $C_4NCH(CH_3)_2$), 3.44 (1H, d-sept, $^3J_{P–H}$ = 12.7 Hz, $^2J_{H–H}$ = 6.3 Hz, $C_5NCH(CH_3)_2$), 2.29 (3H, s, $C_7(CH_3)$), 2.18 (3H, s, $C_2(CH_3)$), 1.64 (3H, s, $C_9(CH_3)$), 1.59 (3H, s, $C_9(CH_3)$), 1.38 (3H, d, $^2J_{H–H}$ = 6.5 Hz, $C_4NCH(CH_3)$), 1.00 (3H, d, $^2J_{H–H}$ = 6.6 Hz, $C_5NCH(CH_3)$), 0.90 (3H, d, $^2J_{H–H}$ = 6.5 Hz, $C_4NCH(CH_3)$), 0.86 ppm (3H, d, $^2J_{H–H}$ = 6.6 Hz, $C_5NCH(CH_3)$).

* signal overlap prevents integration and multiplet analysis

^{1}H{^{31}P} NMR (500 MHz, C_6D_6): ***major isomer*** as above, except δ = 7.02 (1H, s, P–H), 3.54 ppm (1H, sept, $^2J_{H–H}$ = 6.6 Hz, $C_5NCH(CH_3)_2$). ***minor isomer*** as above, except δ = 7.15 (1H, s, P–H), 3.44 ppm (1H, sept, $^2J_{H–H}$ = 6.3 Hz, $C_5NCH(CH_3)_2$).

^{13}C{^{1}H} NMR (151 MHz, C_6D_6): ***major isomer*** δ = 149.4 (s, C_{11}), 139.6 (d, $^2J_{C–P}$ = 6.7 Hz, C_5), 135.5 (d, $^2J_{C–P}$ = 3.6 Hz, C_{4a}), 134.5 (d, $^3J_{C–P}$ = 2.1 Hz, C_{8a}), 133.3 (d, $^2J_{C–P}$ = 9.5 Hz, C_{10a}), 131.3 (s, C_7), 130.9 (s, C_3), 130.0 (s, C_2), 128.4 (s, C_{9a}), 127.0 (s, C_1), 122.3 (s, C_{12}), 116.4 (s, C_8), 112.1 (s, C_{13}), 108.5 (s, C_6), 51.9 (d, $^{5TS}J_{C–P}$ = 13.4 Hz, $C_4NCH(CH_3)_2$)**, 47.5 (d, $^2J_{C–P}$ = 17.4 Hz, $C_5NCH(CH_3)_2$), 36.6 (s, C_9), 34.8 (s, $C_9(CH_3)$), 31.5 (s, $C_9(CH_3)$), 24.3 (s, $C_4NCH(CH_3)$), 22.2 (d, $^3J_{C–P}$ = 10.9 Hz, $C_5NCH(CH_3)$), 21.9 (s, $C_7(CH_3)$), 21.4 (s, $C_5NCH(CH_3)$), 20.9 (s, $C_2(CH_3)$), 19.0 ppm (d, $^{6TS}J_{C–P}$ = 6.1 Hz, $C_4NCH(CH_3)$)**. ***minor isomer*** δ = 149.7 (s, C_{11}), 139.5 (d, $^2J_{C–P}$ = 6.5 Hz, C_5), 134.4 (s, C_{4a}), 133.8 (d, $^3J_{C–P}$ = 2.0 Hz, C_{8a}),

132.8 (d, $^2J_{C–P}$ = 7.8 Hz, C_{10a}), 131.2 (s, C_7), 129.3 (s, C_3), 128.3 (s, C_{9a}), 126.8 (s, C_2), 126.7 (s, C_1), 122.0 (s, C_{12}),116.8 (s, C_8), 112.0 (s, C_{13}), 108.8 (s, C_6), 50.5 (s, $C_4NCH(CH_3)_2$), 47.9 (d, $^2J_{C–P}$ = 18.1 Hz, $C_5NCH(CH_3)_2$), 36.7 (s, C_9), 34.6 (s, $C_9(CH_3)$), 33.0 (s, $C_9(CH_3)$), 25.1 (s, $C_4NCH(CH_3)$), 21.9 (s, $C_7(CH_3)$), 21.6 (d, $^2J_{C–P}$ = 12.2 Hz, $C_5NCH(CH_3)$), 21.3 (s, $C_5NCH(CH_3)$), 20.9 (s, $C_2(CH_3)$), 20.6 ppm (s, $C_4NCH(CH_3)$).

* ^{13}C peak for C_4 could not be identified.

** TS = through space coupling to phosphorus in close proximity.[3]

^{31}P NMR (243 MHz, C_6D_6): ***major isomer*** δ = 67.1 ppm (dd, $^1J_{P–H}$ = 135.0 Hz, $^3J_{P–H}$ = 11.3 Hz). ***minor isomer*** δ = 61.8 ppm (dd, $^1J_{P–H}$ = 148.4 Hz, $^3J_{P–H}$ = 12.3 Hz).

^{31}P{^{1}H} NMR (202 MHz, C_6D_6): ***major isomer*** δ = 67.1 ppm (s). ***minor isomer*** δ = 61.8 ppm (s).

^{11}B{^{1}H} NMR (128 MHz, C_6D_6): δ = −28.6 (br), −34.0 ppm (br).*

*assignment of major and minor isomer could not be achieved.

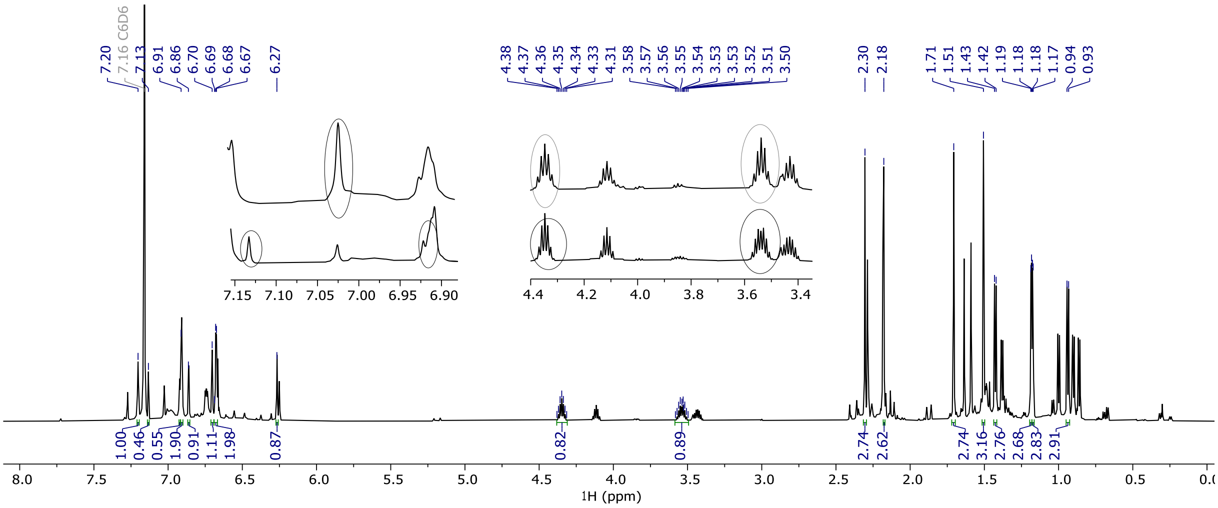

Figure S43: ^{1}H NMR spectrum of **13** in C_6D_6. Only the signals for the major isomer are integrated and picked. Left inset: top is ^{1}H{^{31}P} and bottom is ^{1}H NMR spectrum enhanced between 7.15 – 6.90 ppm. Right inset: top is ^{1}H{^{31}P} and bottom is ^{1}H NMR spectrum enhanced between 4.40 – 3.40 ppm.

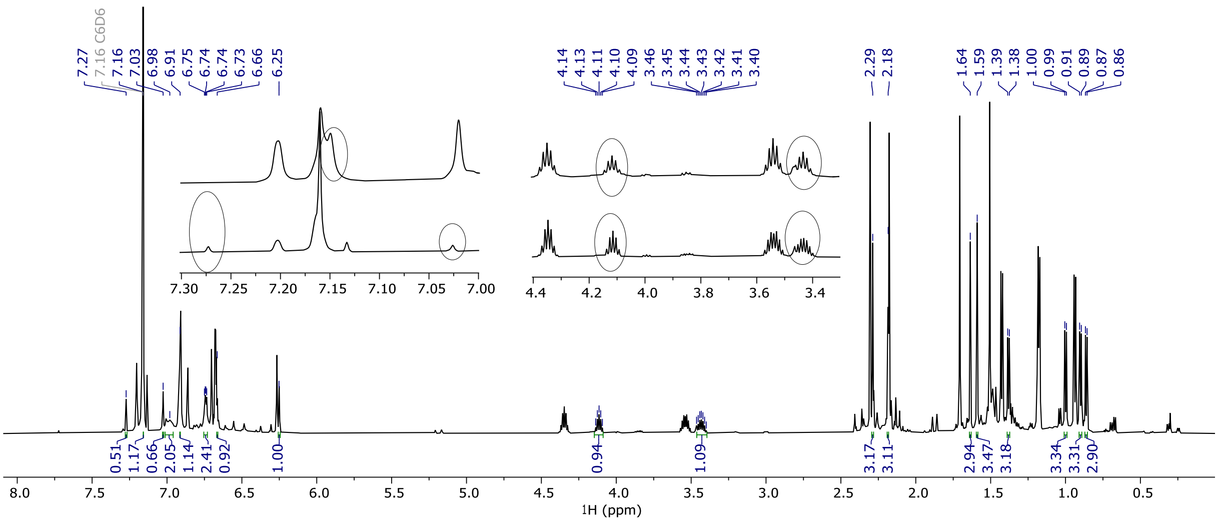

Figure S44: ^{1}H NMR spectrum of **13** in C_6D_6. Only the signals for the minor isomer are integrated and picked. Left inset: top is ^{1}H{^{31}P} and bottom is ^{1}H NMR spectrum enhanced between 7.30 – 7.00 ppm. Right inset: top is ^{1}H{^{31}P} and bottom is ^{1}H NMR spectrum enhanced between 4.40 – 3.40 ppm.

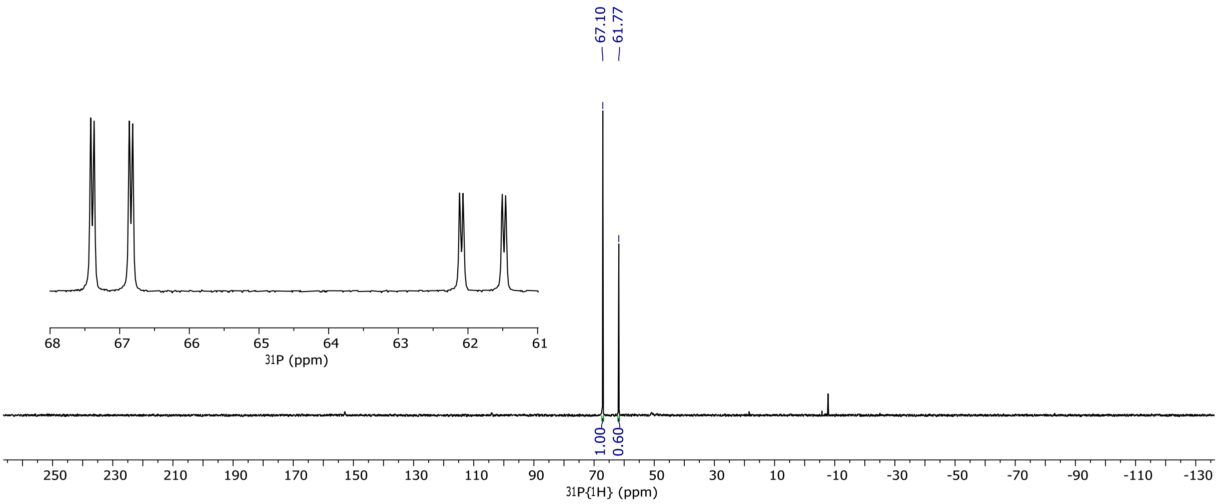

Figure S45: ^{31}P{^{1}H} NMR spectrum of **13** in C_6D_6. Inset: ^{31}P NMR spectrum enhanced between 68.00 – 61.00 ppm.

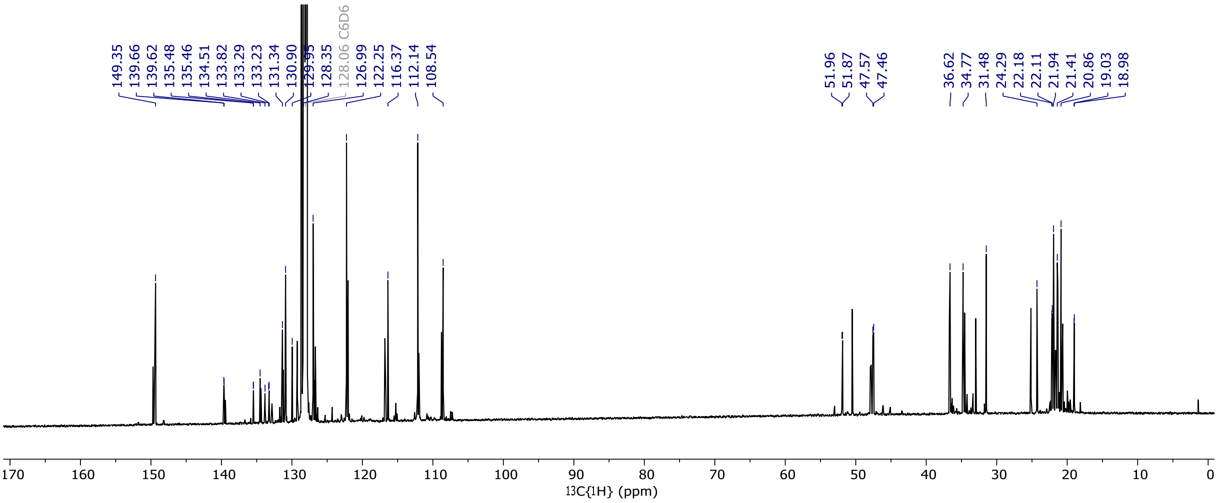

Figure S46: $^{13}C\{^1H\}$ NMR spectrum of **13** in C_6D_6. Only the signals for the major isomer are picked**.**

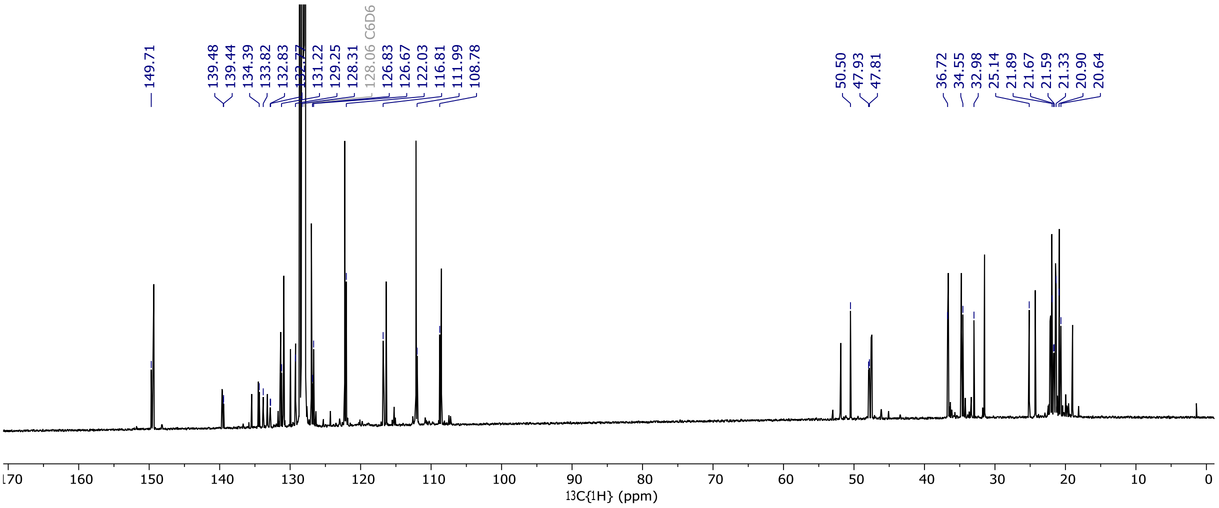

Figure S47: $^{13}C\{^1H\}$ NMR spectrum of **13** in C_6D_6. Only the signals for the minor isomer are picked**.**

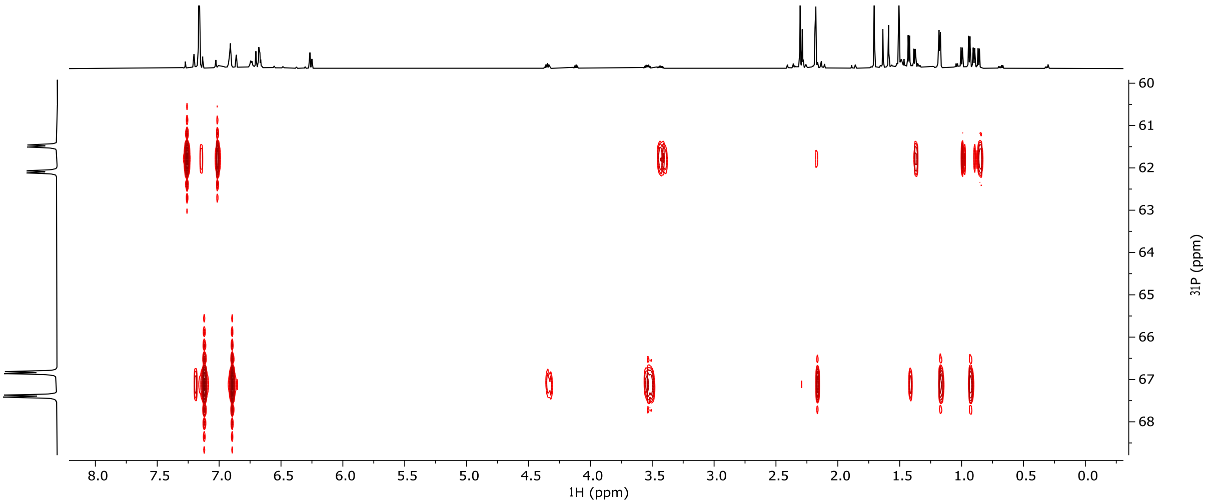

Figure S48: ^{1}H–^{31}P HMBC spectrum of **13** in C_6D_6.

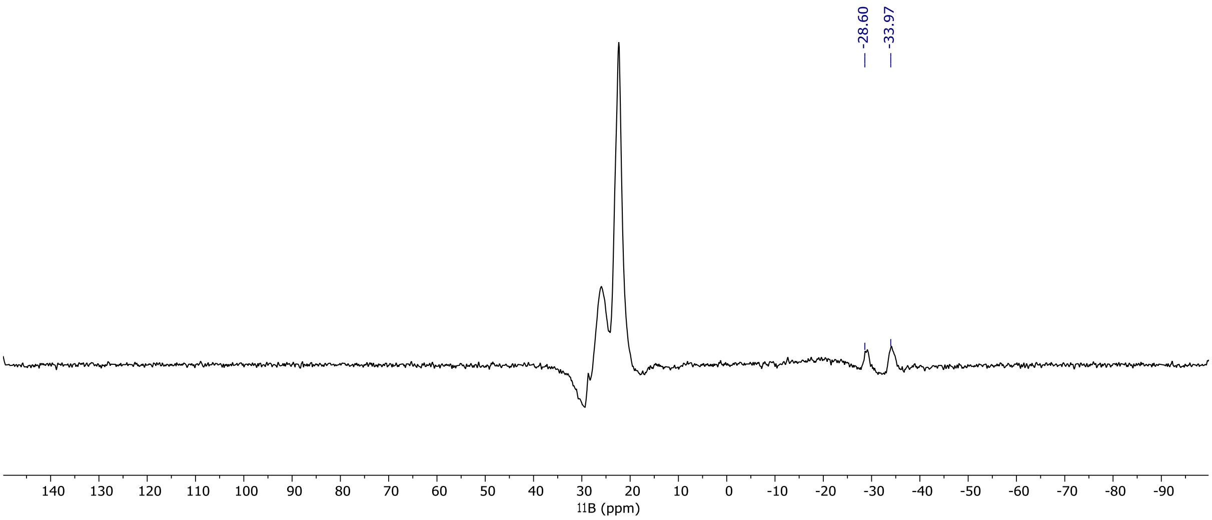

Figure S49: $^{11}B\{^{1}H\}$ NMR spectrum of **13** in C_6D_6.

1.2.15 Synthesis of 14

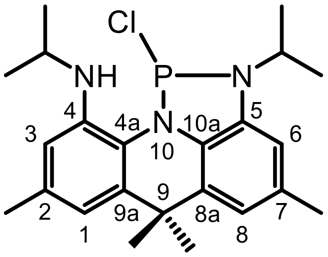

4^{iPr} (10.5 mg, 27.8 µmol, 1.00 eq.) is dissolved in d$_6$-benzene (0.5 mL) in a J. Young NMR tube. Hydrochloric acid (0.2 M in diethyl ether, 0.14 mL, 27.8 µmol, 1.00 eq.) is added, instantly generating a colourless solution. The solvent was removed *in vacuo* and the product dissolved in pentane (0.5 mL). Pale yellow crystals of **14** (8.5 mg, 20.4 µmol, 74%) were grown from a concentrated pentane solution at −30 °C.

^{1}H NMR (500 MHz, C_6D_6): δ = 7.06 (1H, s, C_1H), 6.83 (1H, s, C_8H), 6.58 (1H, s, C_3H), 6.55 (1H, s, C_6H), 3.99 (1H, d-sept, $^3J_{P–H}$ = 11.5 Hz, $^3J_{H–H}$ = 6.7 Hz, $C_5NCH(CH_3)_2$), 3.19 (1H, sept, $^3J_{H–H}$ = 6.2 Hz, $C_4NCH(CH_3)_2$), 2.97 (1H, br s, N–H), 2.33 (3H, s, $C_7(CH_3)$), 2.19 (3H, s, $C_2(CH_3)$), 1.57 (6H, br s, $C_9(CH_3)_2$)*, 1.54 (6H, d, $^3J_{H–H}$ = 6.6 Hz, $C_5NCH(CH_3)_2$), 1.10 ppm (6H, d, $^3J_{H–H}$ = 6.2 Hz, $C_4NCH(CH_3)_2$).

* broad signal indicating overlap between inequivalent methyl groups.

^{1}H{^{31}P} NMR (500 MHz, C_6D_6): as above, except δ = 3.99 ppm (1H, sept, $^3J_{H–H}$ = 6.7 Hz, $C_5NCH(CH_3)_2$).

^{13}C{^{1}H} NMR (151 MHz, C_6D_6): δ = 134.9 (d, $^2J_{C–P}$ = 9.8 Hz, C_5), 134.7 (d, $^2J_{C–P}$ = 3.4 Hz, C_{8a}), 133.6 (s, C_4), 132.4 (s, C^{Ar})**, 132.3 (s, C^{Ar})**, 131.2 (d, $^2J_{C–P}$ = 8.0 Hz, C_{4a}), 130.1 (s, C_{9a}), 129.1 (d, $^2J_{C–P}$ = 12.1 Hz, C_{10a}), 125.2 (s, C_3), 125.0 (s, C_1), 117.9 (s, C_8), 109.1 (s, C_6), 49.0 (d, $^{5TS}J_{C–P}$ = 6.0 Hz, $C_4NCH(CH_3)_2$)***, 47.5 (d, $^2J_{C–P}$ = 15.8 Hz, $C_5NCH(CH_3)_2$), 36.4 (s, C_9), 22.0 (s, $C_7(CH_3)$), 21.1 ppm (s, $C_2(CH_3)$).****

* C_1 assigned by comparison to compounds **13** and **15**.

** due to overlap of 2D spectra, $C_{2/7}$ cannot be distinguished.

*** TS = through space coupling to phosphorus in close proximity.[3]

**** resonance overlap results in a broad signal from 22.3 ppm to 21.4 ppm. 2D spectra indicate resonances corresponding both $C_9(CH_3)_2$ and $C_{4/5}NCH(CH_3)_2$) environments are present in this region.

^{31}P NMR (243 MHz, C_6D_6): δ = 130.6 ppm (m).

^{31}P{^{1}H} NMR (243 MHz, C_6D_6): δ = 130.6 ppm (s).

Elemental analysis calcd. (%) for $C_{23}H_{31}ClN_3P$: C 66.42, H 7.51, N 10.10; found: C 67.21, H 8.24, N 9.01.*

* This is consistent with the presence of 0.5 eq. pentane in the crystalline sample submitted: calcd (%) for $C_{25.5}H_{37.5}Cl_1N_3P_1$: C 67.68, H 8.35, N 9.29.

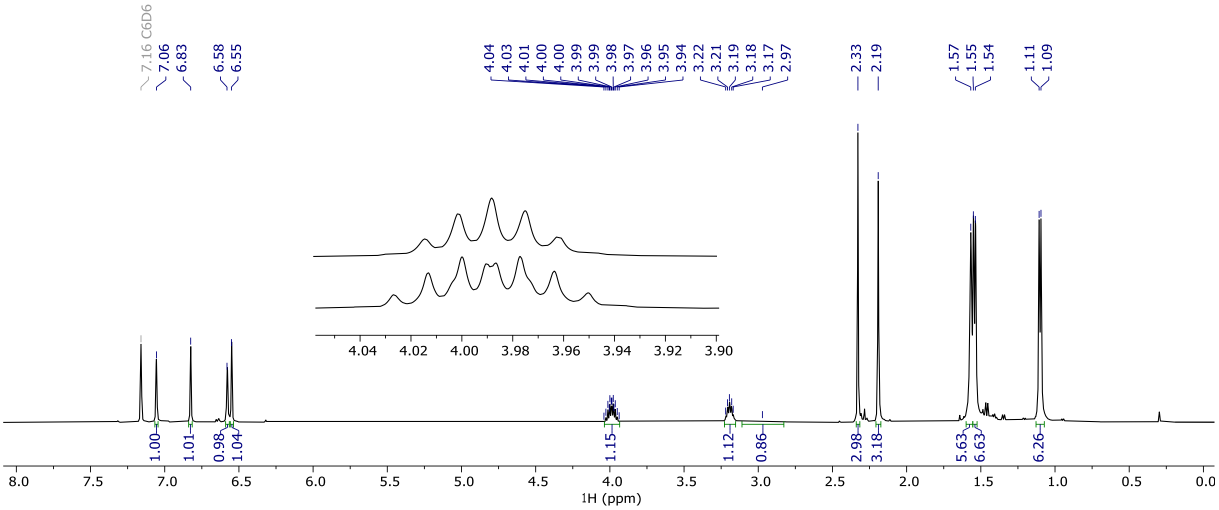

Figure S50: ^{1}H NMR spectrum of **14** in C_6D_6. Inset: top is $^{1}H\{^{31}P\}$ and bottom is ^{1}H NMR spectrum enhanced between 4.05 – 3.90 ppm.

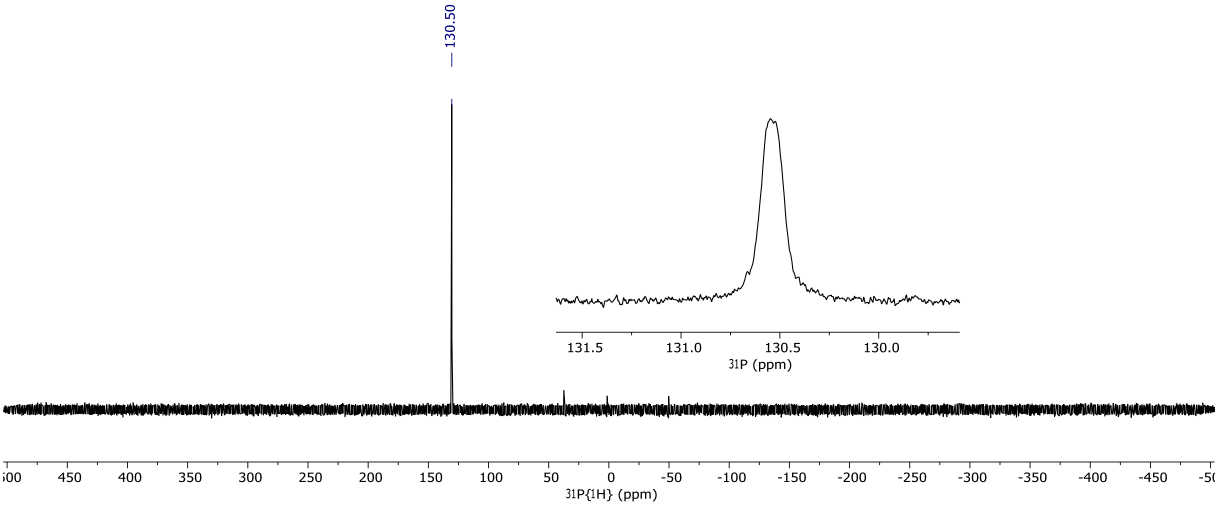

Figure S51: $^{31}P\{^{1}H\}$ NMR spectrum of **14** in C_6D_6. Inset: ^{31}P NMR spectrum enhanced between 131.50 – 129.50 ppm.

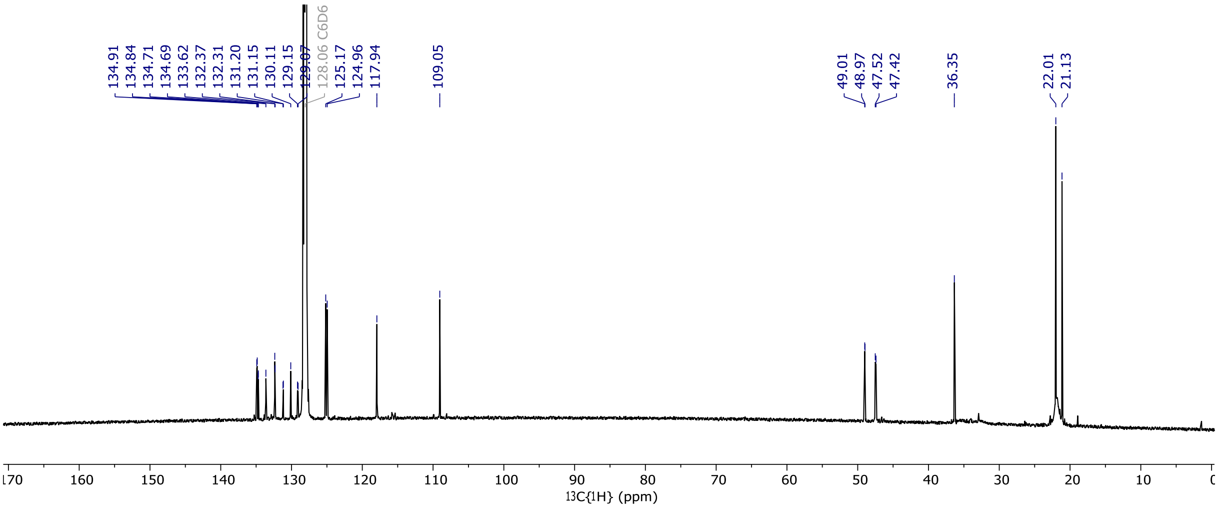

Figure S52: $^{13}C\{^{1}H\}$ NMR spectrum of **14** in C_6D_6.

1.2.16 Synthesis of 15

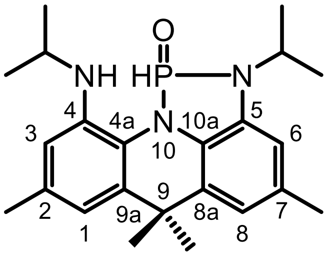

4^{iPr} (31.0 mg, 81.7 µmol, 1.00 eq.) is dissolved in d_6-benzene (0.5 mL) in a J. Young NMR tube. Water (1.5 µL, 81.7 µmol, 1.00 eq.) is added resulting in an immediate colour change to colourless. The solvent is removed *in vacuo* and the remaining residue heated under vacuum to remove traces of water. Lyophilisation from a concentrated benzene solution yields **15** as a colourless powder (25.5 mg, 64.2 µmol, 79%).

^{1}H NMR (400 MHz, C_6D_6): δ = 9.42 (1H, d, $^1J_{P–H}$ = 714.6 Hz, P–*H*), 6.97 (1H, s, C_1H), 6.69 (1H, s, C_8H), 6.64 (1H, s, C_3H), 6.29 (1H, s, C_6H), 3.85 (1H, s, N–H), 3.72 (1H, d-sept, $^3J_{P–H}$ = 18.0 Hz, $^3J_{H–H}$ = 6.7 Hz, $C_5NCH(CH_3)_2$), 3.31 (1H, sept, $^3J_{H–H}$ = 6.3 Hz, $C_4NCH(CH_3)_2$), 2.31 (3H, s, $C_7(CH_3)$), 2.18 (3H, s,$C_2(CH_3)$), 1.64 (3H, s, $C_9(CH_3)$), 1.54 (3H, s, $C_9(CH_3)$), 1.41 (3H, d, $^3J_{H–H}$ = 6.7 Hz, $C_4NCH(CH_3)$), 1.40 (3H, d, $^3J_{H–H}$ = 6.7 Hz, $C_5NCH(CH_3)$), 1.21 (3H, d, $^3J_{H–H}$ = 6.3 Hz, $C_4NCH(CH_3)$), 0.95 ppm (3H, d, $^3J_{H–H}$ = 6.3 Hz, $C_4NCH(CH_3)$).

^{1}H{^{31}P} NMR (400 MHz, C_6D_6): as above, except δ = 9.42 (1H, s, P–H), 3.72 ppm (1H, sept, $^3J_{H–H}$ = 6.7 Hz, $C_5NCH(CH_3)_2$).

^{13}C{^{1}H} NMR (151 MHz, C_6D_6): δ = 133.6 (s, C_4), 133.0 (d, $^2J_{C–P}$ = 8.6 Hz, C_{10a}), 132.9 (d, $^2J_{C–P}$ = 5.7 Hz, C_{8a}), 131.9 (s, C_2), 131.5 (s, C_7), 129.9 (s, C_{4a}), 127.3 (d, $^3J_{C–P}$ = 5.9 Hz, C_{9a}), 125.1 (d, $^2J_{C–P}$ = 10.8 Hz, C_5), 123.9 (s, C_1), 121.8 (s, C_3), 116.3 (s, C_8), 106.6 (d, $^3J_{C–P}$ = 6.16 Hz, C_6), 47.0 (s, $C_4NCH(CH_3)_2$)), 46.3 (d, $^2J_{C–P}$ = 5.0 Hz, $C_5NCH(CH_3)_2$), 36.2 (s, C_9), 34.5 (s, $C_9(CH_3)$), 34.0 (s, $C_9(CH_3)$), 22.6 (s, $C_4NCH(CH_3)$), 22.1 (s, $C_7(CH_3)$), 21.8 (s, $C_5NCH(CH_3)$), 21.2 (s, $C_2(CH_3)$), 21.0 (s, $C_4NCH(CH_3)$), 20.0 ppm (d, $^3J_{C–P}$ = 3.73 Hz, $C_5NCH(CH_3)$).

^{31}P NMR (162 MHz, C_6D_6): δ = 1.3 ppm (dd, $^1J_{P–H}$ = 714.6 Hz, $^3J_{P–H}$ = 18.3 Hz)

^{31}P{^{1}H} NMR (162 MHz, C_6D_6): δ = 1.3 ppm (s)

Elemental analysis calcd. (%) for $C_{23}H_{32}N_3OP$: C 69.50, H 8.11, N 10.57; found: C 68.72, H 7.68, N 9.97.*

* This is consistent with the presence of 0.25 eq. water in the crystalline sample submitted: calcd (%) for $C_{23}H_{32.5}N_3O_{1.25}P_1$: C 68.72, H 8.15, N 10.45.

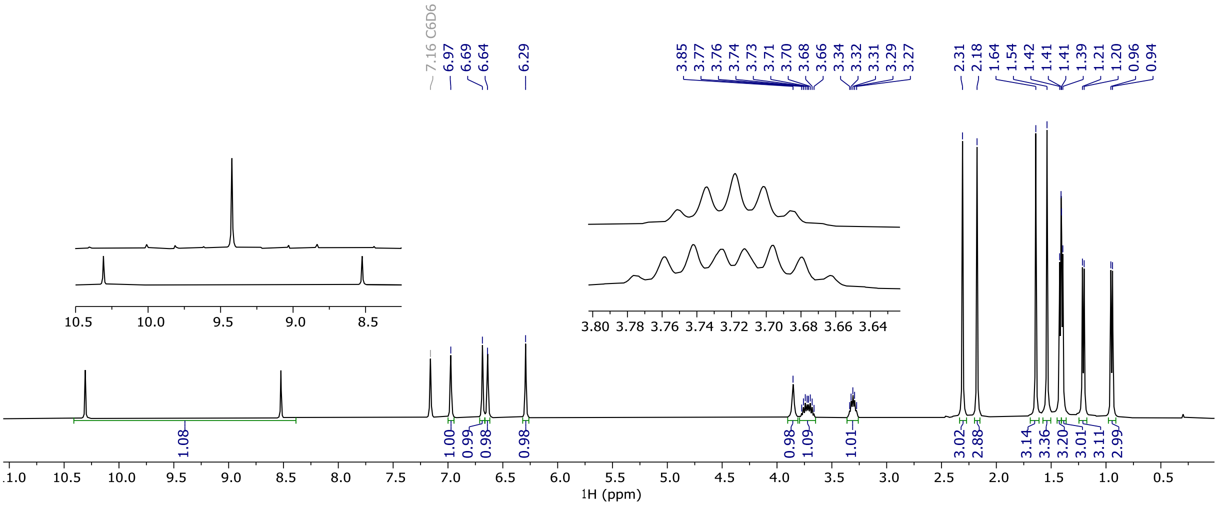

Figure S53: ^{1}H NMR spectrum of **15** in C_6D_6. Left inset: top is $^{1}H\{^{31}P\}$ and bottom is ^{1}H NMR spectrum enhanced between 10.50 – 8.25 ppm. Right inset: top is $^{1}H\{^{31}P\}$ and bottom is ^{1}H NMR spectrum enhanced between 3.80 – 3.62 ppm.

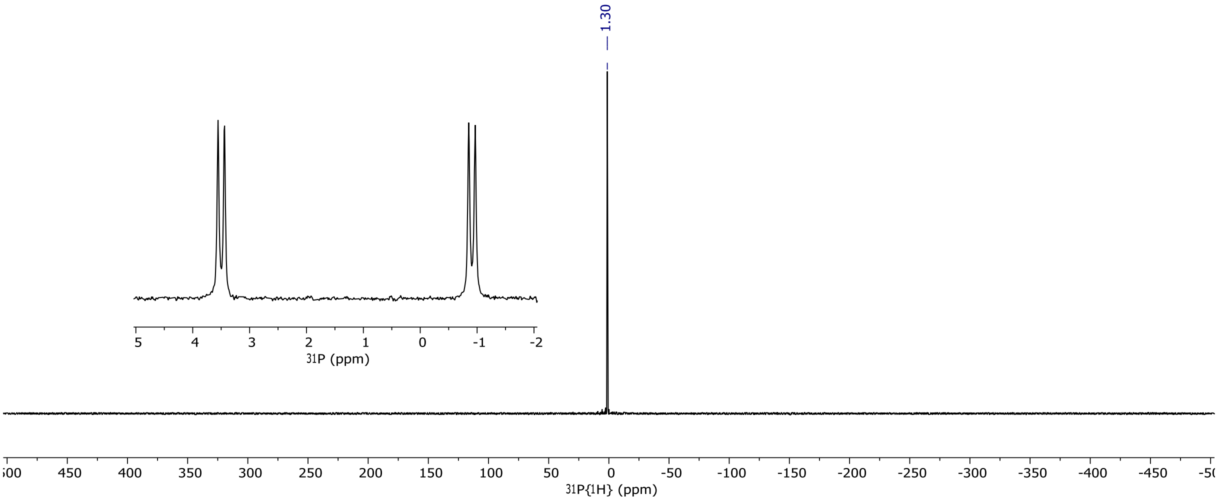

Figure S54: $^{31}P\{^{1}H\}$ NMR spectrum of **15** in C_6D_6. Inset: ^{31}P NMR spectrum enhanced between 5.00 – –2.00 ppm.

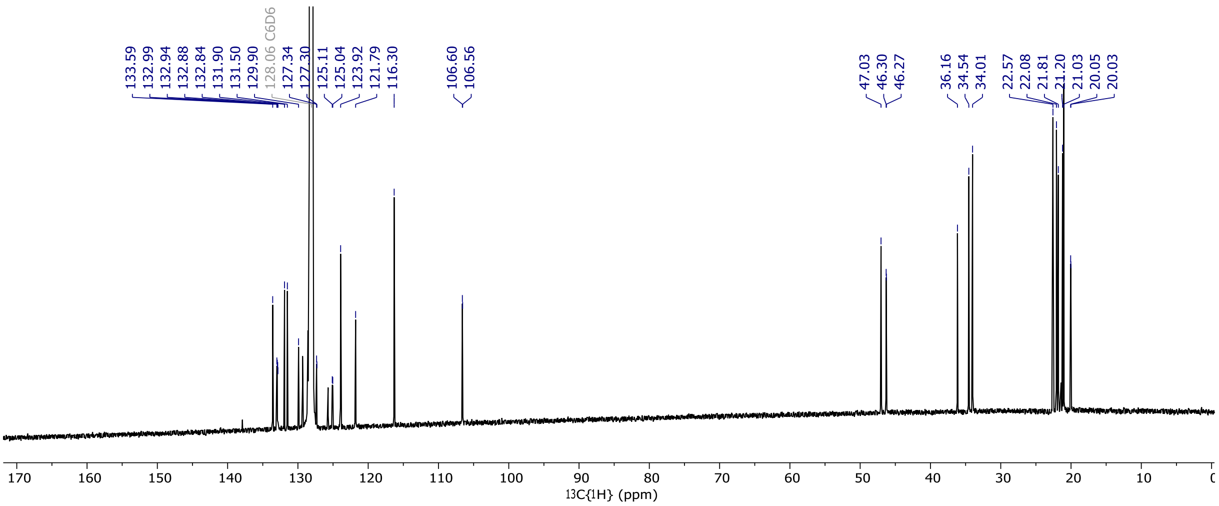

Figure S55: $^{13}C\{^{1}H\}$ NMR spectrum of **15** in C_6D_6.

1.2.17 Synthesis of 16

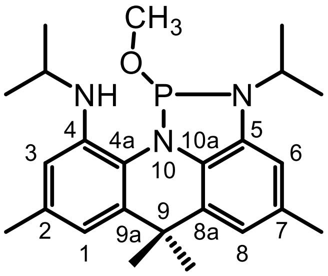

4^{iPr} (10.5 mg, 27.8 µmol, 1.00 eq.) is dissolved in d_6-benzene (0.5 mL) in a J. Young NMR tube. Methanol (2 M in DME, 14.0 µL, 27.8 µmol, 1.00 eq.) is added resulting in the generation of a pale-yellow solution. The solvent is removed *in vacuo* and the residue extracted with pentane (2 × 0.5 mL). Crystals of **16** (8.5 mg, 20.7 µmol, 74%) are grown from a concentrated pentane solution at −30 °C.

^{1}H NMR (600 MHz, C_6D_6): δ = 7.02 (1H, s, C_8H), 6.79 (1H, s, C_1H), 6.57 (1H, s, C_6H), 6.43 (1H, s, C_3H), 3.92 (1H, d-sept, $^3J_{P–H}$ = 10.2 Hz, $^3J_{H–H}$ = 6.7 Hz, $C_5NCH(CH_3)_2$)), 3.47 – 3.39 (2H, overlapping multiplet corresponding to N*H* and $C_4NCH(CH_3)_2$) environments), 2.81 (3H, d, $^3J_{P–H}$ = 7.4 Hz, P–OCH_3), 2.39 (3H, s, $C_2(CH_3)$), 2.28 (3H, s, $C_7(CH_3)$), 1.76 (3H, s, $C_9(CH_3)$), 1.72 (3H, s, $C_9(CH_3)$), 1.43 (3H, dd, $^3J_{H–H}$ = 6.7 Hz, $^4J_{P–H}$ = 1.1 Hz, $C_5NCH(CH_3)$), 1.38 (3H, d, $^3J_{H–H}$ = 6.7 Hz, $C_5NCH(CH_3)$), 1.19 – 1.17 ppm (6H, overlapping doublets corresponding to both $C_4NCH(CH_3)$ environments).

^{1}H{^{31}P} NMR (600 MHz, C_6D_6): as above, except δ = 3.92 (1H, sept, $^3J_{H–H}$ = 6.7 Hz, $C_5NCH(CH_3)_2$)), 2.81 (3H, s, P–OCH_3), 1.43 ppm (3H, d, $^3J_{H–H}$ = 6.7 Hz, $C_5NCH(CH_3)$)).

^{13}C{^{1}H} NMR (151 MHz, C_6D_6): δ = 136.4 (d, $^2J_{C–P}$ = 7.8 Hz, C_5), 134.4 (d, $^3J_{C–P}$ = 1.5 Hz, C_4), 133.4 (d, $^3J_{C–P}$ = 2.1 Hz, $C_{8a/9a}$), 130.8 (s, C_7), 130.7 (s, C_2), 130.1 (d, $^2J_{C–P}$ = 8.8 Hz, C_{4a}), 128.6 (s, C_{10a}), 127.0 (s, $C_{8a/9a}$), 121.3 (s, C_8), 119.0 (s, C_6), 116.2 (s, C_1), 106.1 (s, C_3), 49.7 (d, $^2J_{C–P}$ = 5.4 Hz, P–OCH_3), 46.7 (d, $^2J_{C–P}$ = 19.5 Hz, $C_5NCH(CH_3)_2$), 46.5 (d, $^{5TS}J_{C–P}$ = 5.9 Hz, $C_4NCH(CH_3)_2$),* 36.6 (s, C_9), 34.7 (s, $C_9(CH_3)$), 34.6 (s, $C_9(CH_3)$), 22.7 – 22.6 (overlapping multiplets, $C_5NCH(CH_3)_2$), 22.4 (s, $C_4NCH(CH_3)_2$), 22.1 (s, $C_2(CH_3)$), 21.3 ppm (s, $C_7(CH_3)$).

* TS = through space coupling to phosphorus in close proximity.[3]

^{31}P NMR (243 MHz, C_6D_6): δ = 97.3 ppm (m).

^{31}P{^{1}H} NMR (243 MHz, C_6D_6): δ = 97.3 ppm (s).

Elemental analysis calcd. (%) for $C_{24}H_{34}N_3OP$: C 70.05, H 8.33, N 10.21; found: C 70.15, H 8.44, N 9.80.

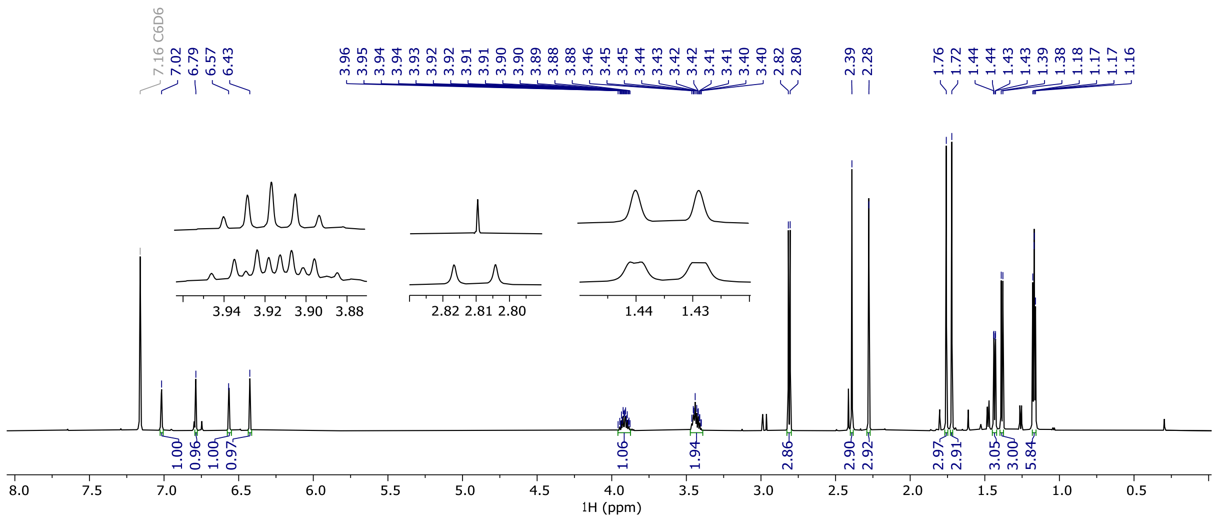

Figure S56: ^1H NMR spectrum of **16** in C_6D_6. Left inset: top is $^1H\{^{31}P\}$ and bottom is ^1H NMR spectrum enhanced between 3.96 – 3.87 ppm. Middle inset: top is $^1H\{^{31}P\}$ and bottom is ^1H NMR spectrum enhanced between 2.83 – 2.79 ppm. Right inset: top is $^1H\{^{31}P\}$ and bottom is ^1H NMR spectrum enhanced between 1.45 – 1.42 ppm.

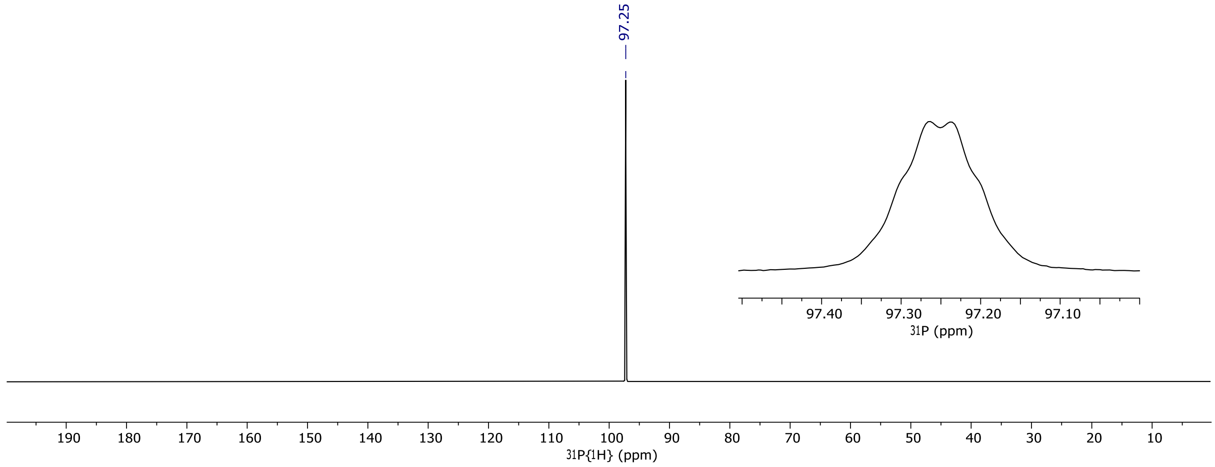

Figure S57: $^{31}P\{^1H\}$ NMR spectrum of **16** in C_6D_6. Inset: ^{31}P NMR spectrum enhanced between 97.50 – 97.00 ppm.

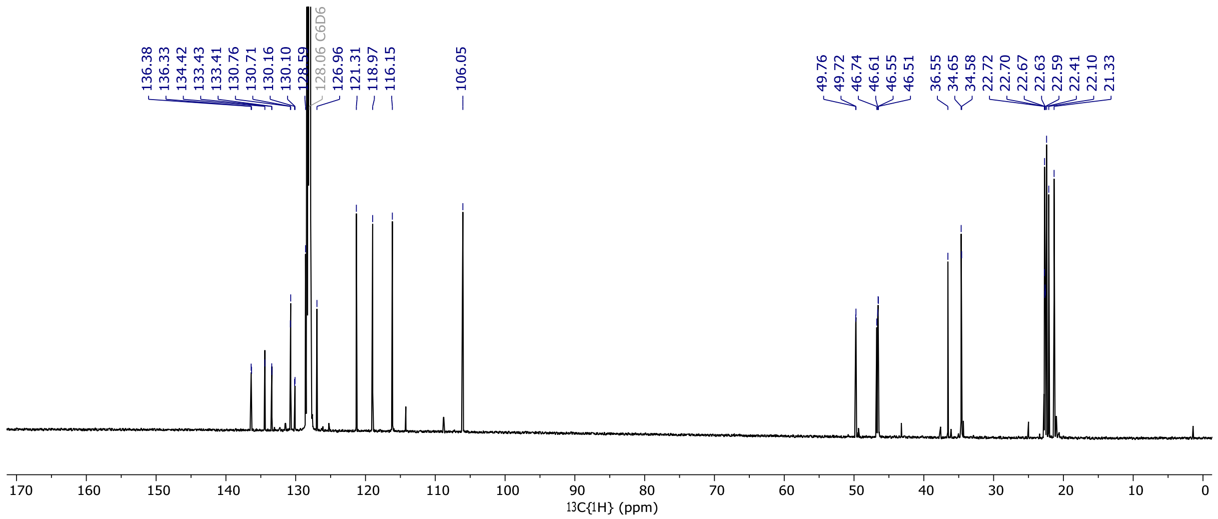

Figure S58: $^{13}C\{^{1}H\}$ NMR spectrum of **16** in C_6D_6.

2. Additional NMR spectra

2.1 Reaction between 3^{Me} and PMe_3

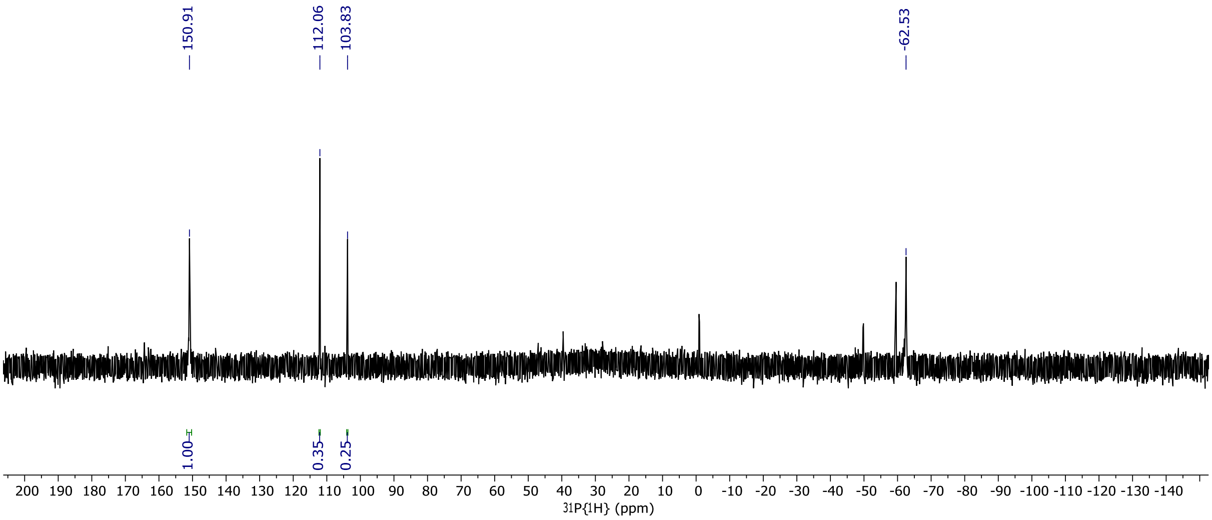

Figure S59: $^{31}P\{^1H\}$ NMR spectrum of the reaction between $\mathbf{3^{Me}}$ and PMe_3 (1.5 eq.) in C_6D_6. This spectrum was obtained immediately after PMe_3 addition whereby a bright orange solution and white precipitate was obtained. The bright orange colour is comparable to the colour of $\mathbf{4^{\mathit{i}Pr}}$, confirmed spectroscopically and crystallographically to be a C_{2v} symmetric phosphine. In addition, the chemical shift of the major product at 150.91 ppm is also comparable to $\mathbf{4^{\mathit{i}Pr}}$ (which has a $^{31}P\{^1H\}$ NMR chemical shift in C_6D_6 of 144.99 ppm (br s)). Minor products at 112.06 and 103.83 ppm are observed, the former of which is dimeric phosphine **2** while the latter remains unknown. The presence of **2** indicates that dimerisation occurs upon dehalogenation/reduction of $\mathbf{3^{Me}}$. Unreacted PMe_3 is also observed at −62.53 ppm. The orange colour dissipates after approximately 10 minutes, suggesting the decomposition of a transiently generated C_{2v} phosphine. The major product of this decomposition is dimeric **2** as shown below.

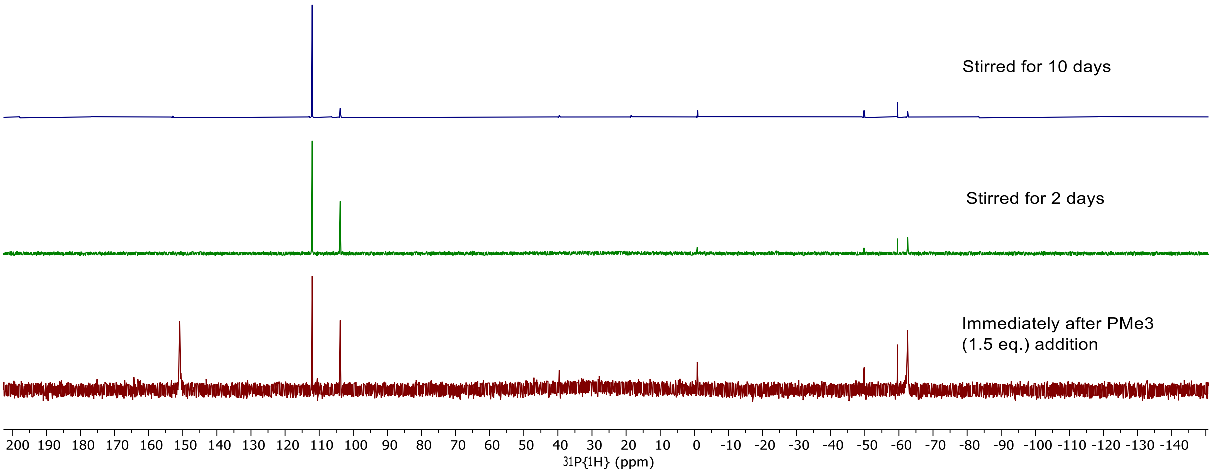

Figure S60: $^{31}P\{^1H\}$ NMR spectrum of the reaction between $\mathbf{3^{Me}}$ and PMe_3 (1.5 eq.) in C_6D_6 at different time intervals. After stirring for 2 days, the peak at 150.91 ppm (corresponding to a suggested C_{2v} symmetric phosphine) disappears and the species at 112.06 and 103.83 ppm grow in intensity. Further stirring for 10 days results in the peak at 103.83 ppm diminishing

and the peak at 112.06 ppm growing. This major product is dimeric **2**, implying that the minor product at 103.83 ppm is a related structure which undergoes slow reaction towards **2**.

2.2 Reaction between acridane derived ligand 1^{iPr} and PCl_3

The experimental procedure is as follows: $\mathbf{1}^{iPr}$ (30.0 mg, 85.3 µmol, 1.00 eq.) is dissolved in toluene (5 mL) and cooled to −78 °C. This solution is added to a separate toluene (5 mL) solution of phosphorus trichloride (0.559 M in hexane, 0.15 mL, 85.3 mmol, 1.00 eq.) and triethyl amine (60.0 µL, 0.427 mmol, 5.00 eq.) at −78 °C. The mixture is gradually warmed to room temperature and stirred for 1 hour, resulting in the generation of a pale-yellow solution and off-white precipitate. The solution is then filtered and the precipitate extracted with toluene (3 × 5 mL). The solvent is removed from the combined extracts *in vacuo* and the residue dissolved in C_6D_6 for NMR analysis.

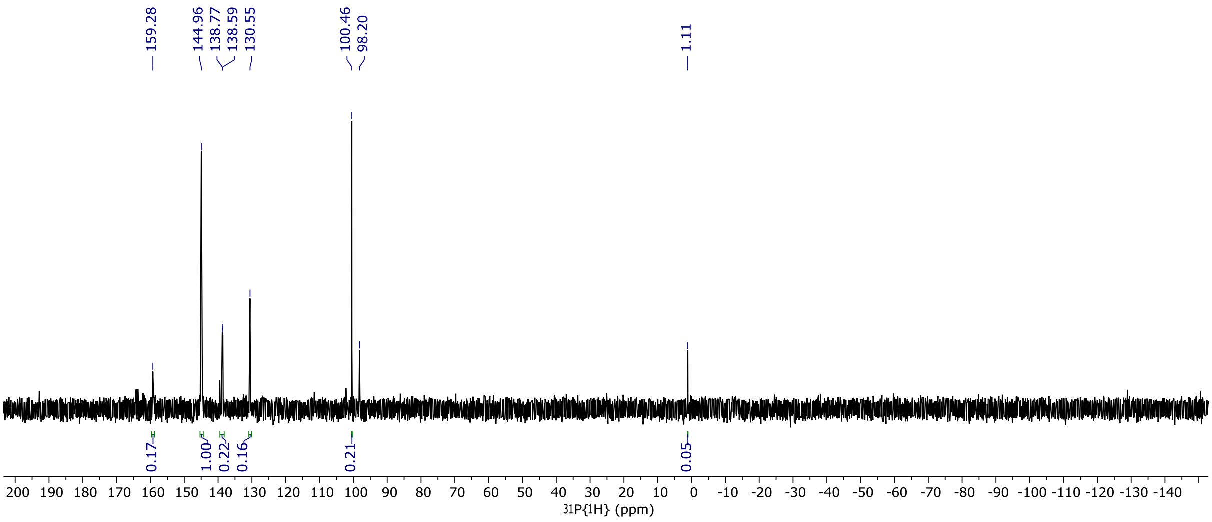

Figure S61: $^{31}P\{^1H\}$ NMR spectrum of the resulting product mixture from reaction between $\mathbf{1}^{iPr}$ and PCl_3 (1.00 eq.) (C_6D_6 is used as the NMR solvent). The major product has a chemical shift of 144.96 ppm corresponding to C_{2v} symmetric phosphine $\mathbf{4}^{iPr}$. The peaks at 130.55 ppm and 1.11 ppm correspond to compounds **14** and **15** respectively, and the peak at 100.46 ppm likely corresponds to the isopropyl substituted ligand analogue of dimeric **2**. Species giving rise to the remaining peaks remain unknown.

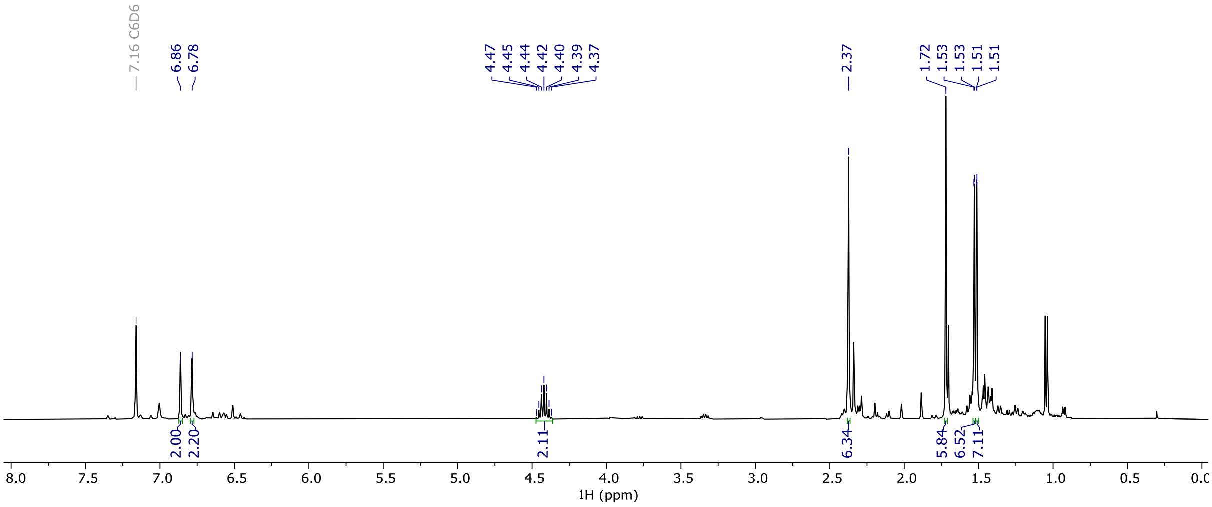

Figure S62: ^1H NMR spectrum of the resulting product mixture from reaction between $\mathbf{1}^{iPr}$ and PCl_3 (1.00 eq.) (C_6D_6 is used as the NMR solvent). The major product has resonances corresponding to C_{2v} symmetric phosphine $\mathbf{4}^{iPr}$ (the picked and integrated signals).

2.3 Dimerisation of 4^{iPr} under ambient conditions

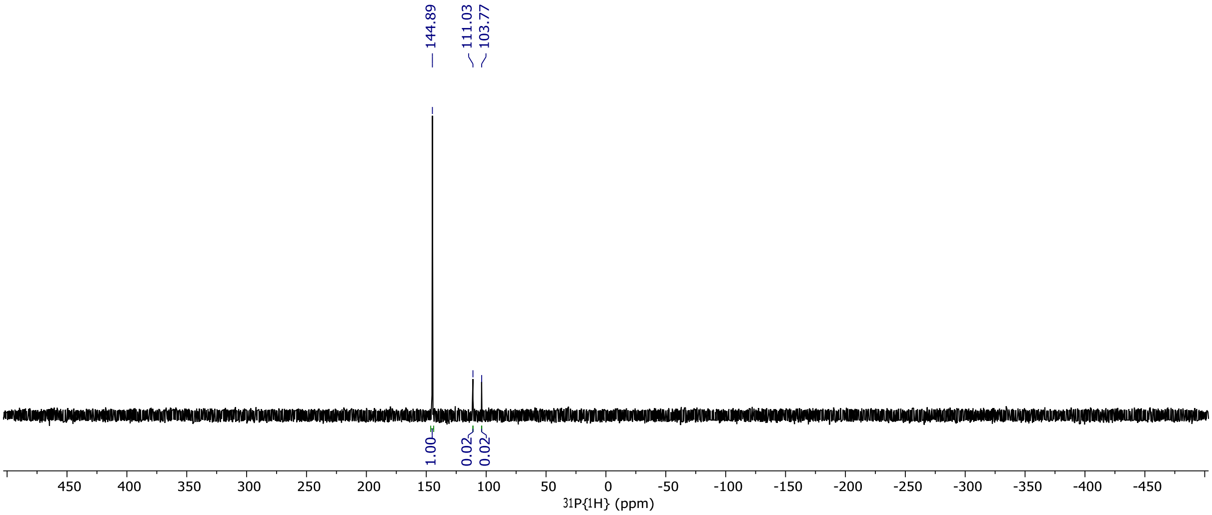

Figure S63: $^{31}P\{^{1}H\}$ NMR spectrum of a sample of $\mathbf{4^{iPr}}$ in C_6H_6 three days after dissolution. The formation of minor quantities (by integration) of new phosphorus-containing products is observed. The chemical shift range of these new signals is similar to that of dimeric **2**. Therefore, we tentatively attribute these new signals as those corresponding to a similar dimeric phosphine baring the isopropyl substituted ligand $\mathbf{1^{iPr}}$ (potentially an isomeric mixture, hence the appearance of two signals).

2.4 Reaction between 4^{iPr} and O_2

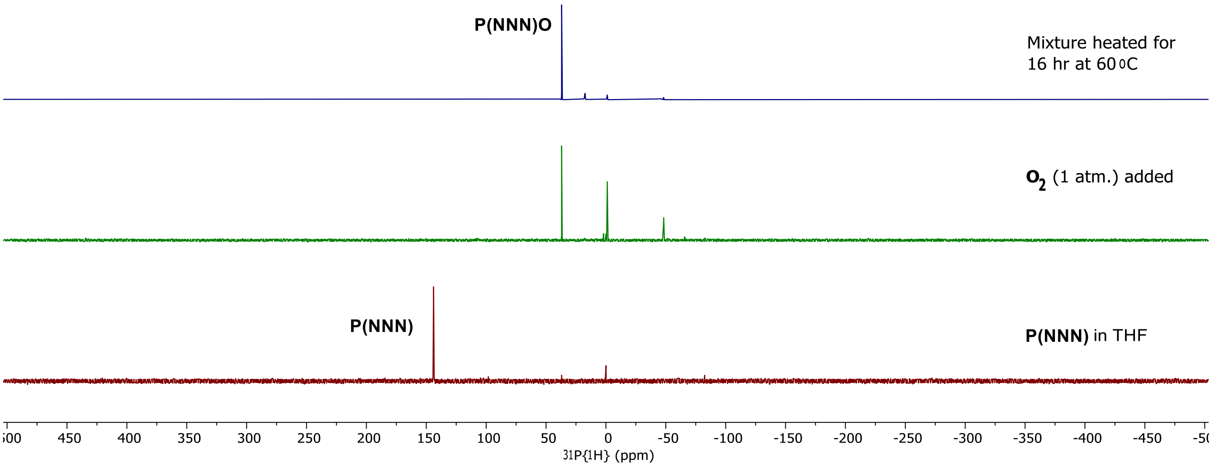

Figure S64: *In-situ* reaction monitoring by $^{31}P\{^{1}H\}$ NMR of the reaction between $\mathbf{4^{iPr}}$ and O_2 (1 atm.) in tetrahydrofuran (THF). **Bottom spectra**: T-shaped phosphine $\mathbf{4^{iPr}}$ in THF, note the presence of a minor peak at 0.05 ppm corresponding to **15** – a result of hydrolysis due to water contamination in the reaction solvent. **Middle spectra**: the result of degassing the reaction solution and charging the NMR tube with 1 atmosphere of O_2. A peak at 36.97 ppm indicates the formation of phosphine oxide **7**. Two additional peaks are observed at −1.22 (d, $^{2}J_{P-P}$ = 30.3 Hz) and −48.20 ppm (d, $^{2}J_{P-P}$ = 30.3 Hz) in a 1:1 ratio by integration. These peaks indicate the formation of a dimeric phosphine oxide, closely resembling the reaction product of a bent geometrically constrained phosphine with O_2 as reported by Goicoechea and

Aldridge and co-workers.[4] **Top spectra**: The result of heating the reaction solution at 60 °C for 16 hours. Full conversion to phosphine oxide **7** is observed.

2.5 Reaction between $\mathbf{4^{\mathit{i}Pr}}$ and S_8

Compositionally pure **8** could not be obtained by reaction between $\mathbf{4^{\mathit{i}Pr}}$ and S_8 on account of the excess S_8 used, resulting in the co-crystallization of sulfur with the product. Therefore, NMR spectra of the reaction mixture are provided to demonstrate the selective reactivity under these conditions towards **8**.

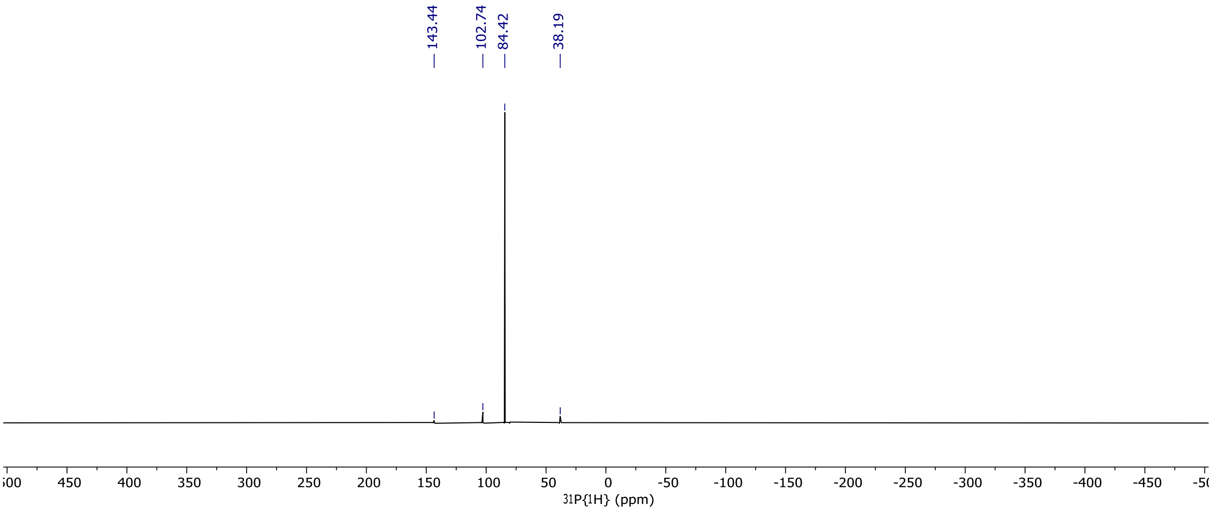

Figure S65: $^{31}P\{^{1}H\}$ NMR spectrum of $\mathbf{4^{\mathit{i}Pr}}$ with S_8 (5 eq.) in CD_2Cl_2 following 4 hours stirring at 25 °C. Major peak at 84.42 ppm corresponds to thiophosphorane **8**, with minor peaks at 143.44, 102.74 and 38.19 ppm corresponding to; unreacted $\mathbf{4^{\mathit{i}Pr}}$, suspected dimeric form of $\mathbf{4^{\mathit{i}Pr}}$, and phosphine oxide **7** respectively.

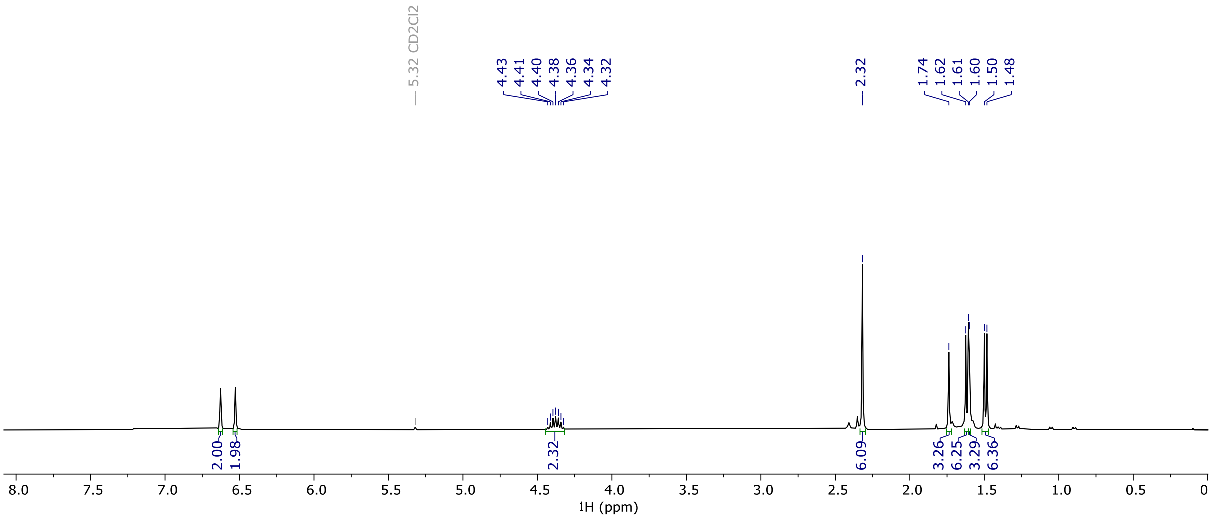

Figure S66: ^{1}H NMR spectrum of $\mathbf{4^{\mathit{i}Pr}}$ with S_8 (5 eq.) in CD_2Cl_2 following 4 hours stirring at 25 °C. Peaks corresponding to **8** are observed as the major product.

2.6 Reaction between 12 and benzaldehyde

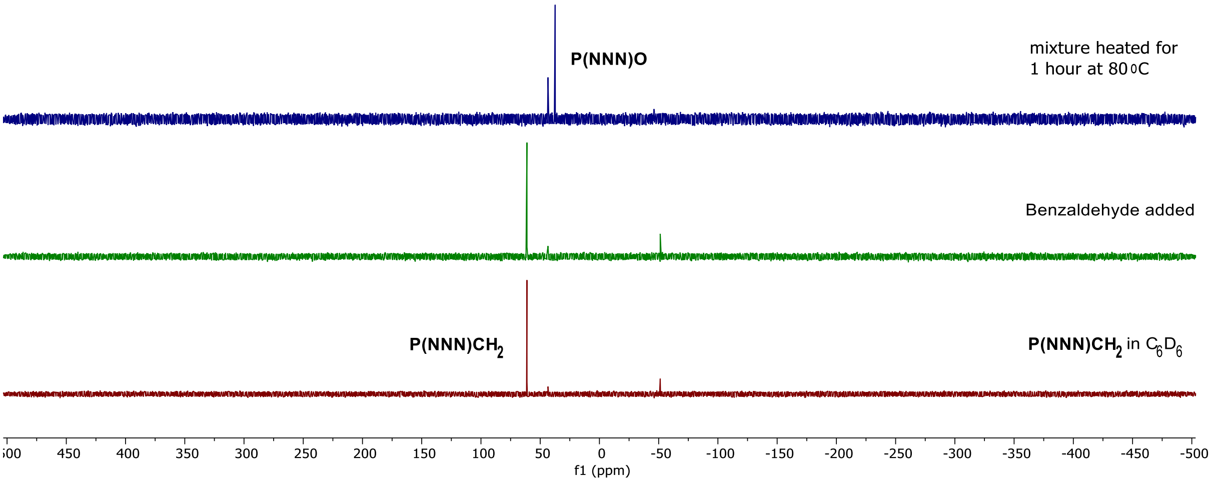

Figure S67: *In-situ* reaction monitoring by $^{31}P\{^{1}H\}$ NMR of the reaction between **12** and benzaldehyde (1 eq.) in C_6D_6. **Bottom spectra**: geometrically constrained ylide **12** in C_6D_6, note the presence of a minor peak at 51.31 ppm of unknown origin. **Middle spectra**: the result of adding benzaldehyde (1 eq.) to the solution. No change is observed. **Top spectra**: The result of heating the reaction solution at 80 °C for 1 hour. Full consumption of the starting material is observed, along with the generation of phosphine oxide **7** as the major product. Unknown product at 43.43 ppm is observed (0.33 integration with respect to **7**). This species is tentatively assigned as the hydrolysis product of **12** as a result of water contamination in the benzaldehyde used.

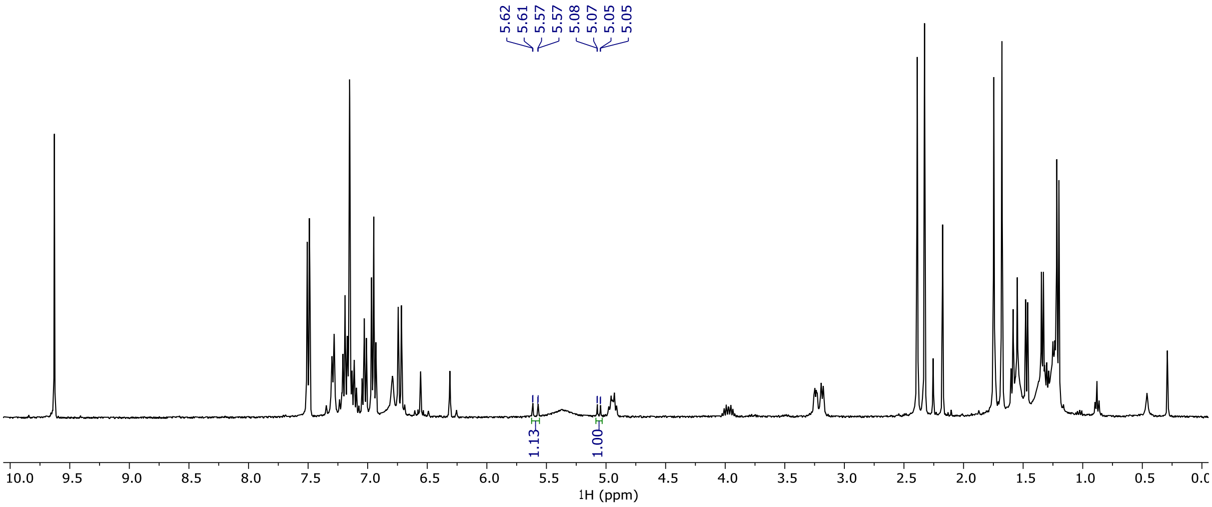

Figure S68: The respective ^{1}H NMR spectra following heating a C_6D_6 solution of **12** with benzaldehyde to 80 °C for 1 hour as described above. The picked and integrated peaks correspond to the terminal alkenic protons of styrene, confirming its formation via Wittig reaction between the starting materials (the remaining styrene peaks are obstructed by overlap therefore could not be integrated). Note the presence of unreacted benzaldehyde, **7** and another unknown species (potentially corresponding to the species with a peak at 44.43 ppm in the $^{31}P\{^{1}H\}$ NMR spectrum).

2.7 Decomposition of 13

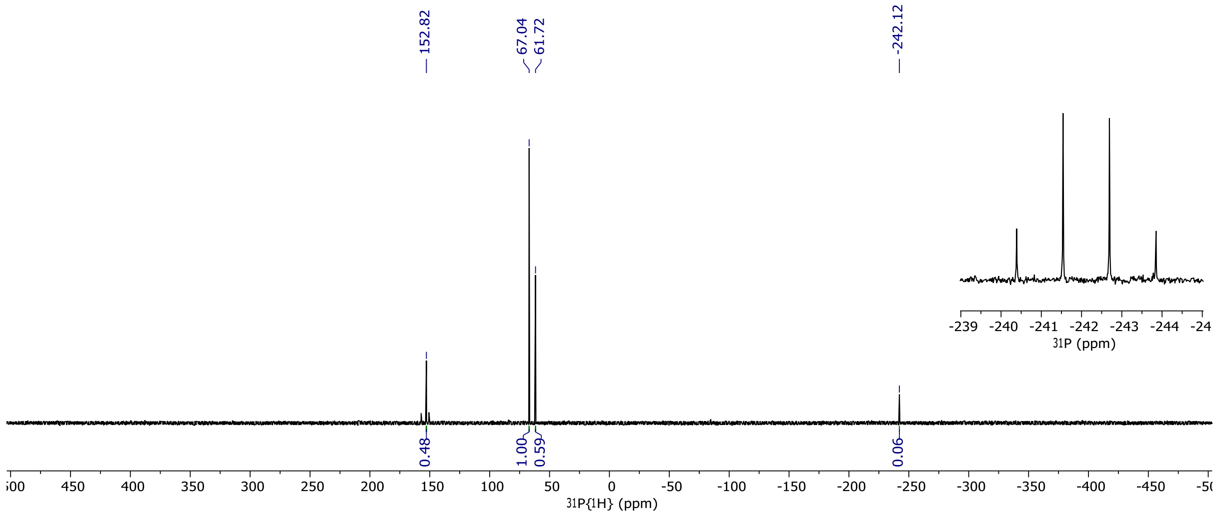

Figure S69: $^{31}P\{^1H\}$ NMR spectra of **13** in C_6D_6 after being left in solution overnight. For comparison, this is the sample that was used for full NMR characterisation of **13** (**Section 1.2.14**, **Figure S43**). The ratio of isomers (peaks with chemical shift 67.04 and 61.72 ppm) remains almost unchanged under these conditions. Additionally, no signals indicative of phosphorus(V) species were generated. Partial decomposition instead occurred; PH_3 is observed at −242.12 ppm which is split into a quartet in the non-proton-decoupled ^{31}P spectra (shown in the spectra inset). An unknown signal at 152.82 ppm is also observed.

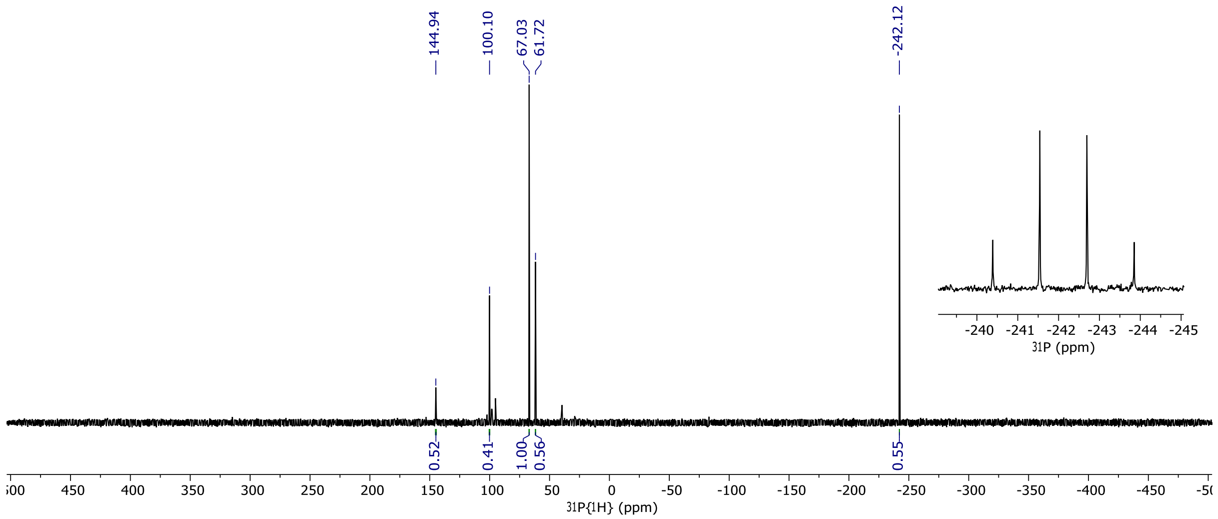

Figure S70: $^{31}P\{^1H\}$ NMR spectra of **13** in C_6D_6 following heating at 80 °C for 4 days. As above, the ratio of isomers (peaks with chemical shift 67.03 and 61.72 ppm) remains almost unchanged under these conditions and no signals indicative of phosphorus(V) species were generated. Decomposition was instead accelerated; PH_3 (at −242.12 ppm) was observed in a greater proportion. A signal at 144.94 ppm corresponding to $\mathbf{4}^{iPr}$ is also observed, along with a signal at 100.10 ppm (potentially corresponding to the dimeric form of $\mathbf{4}^{iPr}$ by comparison to the chemical shift of **2**).

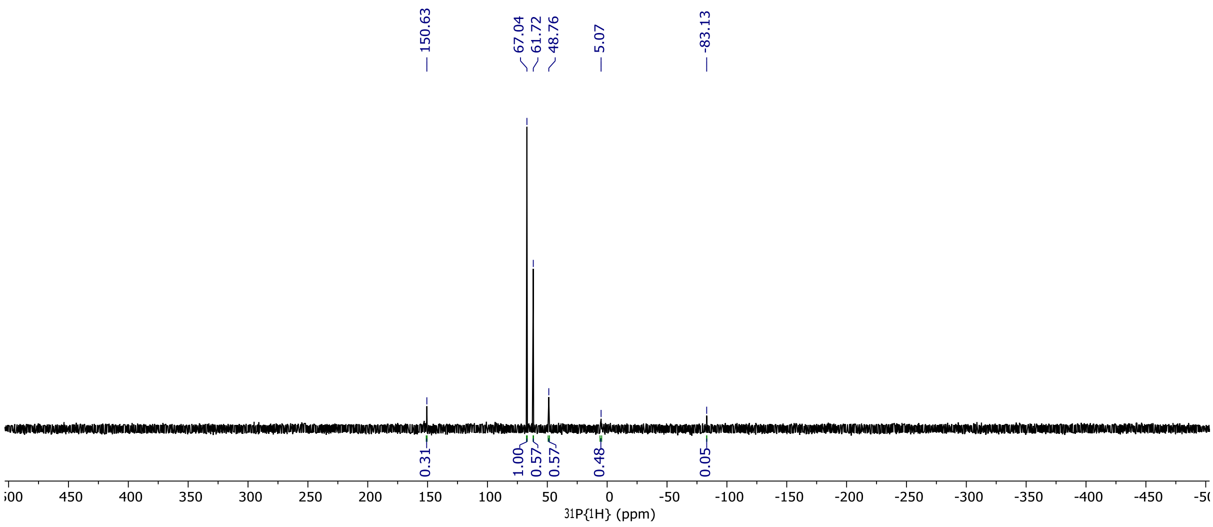

Figure S71: $^{31}P\{^1H\}$ NMR spectra of **13** in C_6D_6 after subjecting a solid sample to vacuum for 1 hour. Significant decomposition to unknown products is observed.

The instability of a solid sample of **13** under vacuum (**Figure S71**), and the previously shown instability in solution (**Figures S69** and **S70**) precluded the preparation of a suitable sample of **13** for elemental analysis. This is further hindered by the inability to obtain a crystalline sample of **13**. This is confirmed by the elemental analysis values obtained from a solid sample of X (9.0 mg, off-white powder):

Elemental analysis calcd. (%) for $C_{29}H_{35}BN_3O_2P$: C 69.75, H 7.06, N 8.41; found: C 67.74, H 6.43, N 7.29.

2.8 D_2O activation product characterisation (15^D)

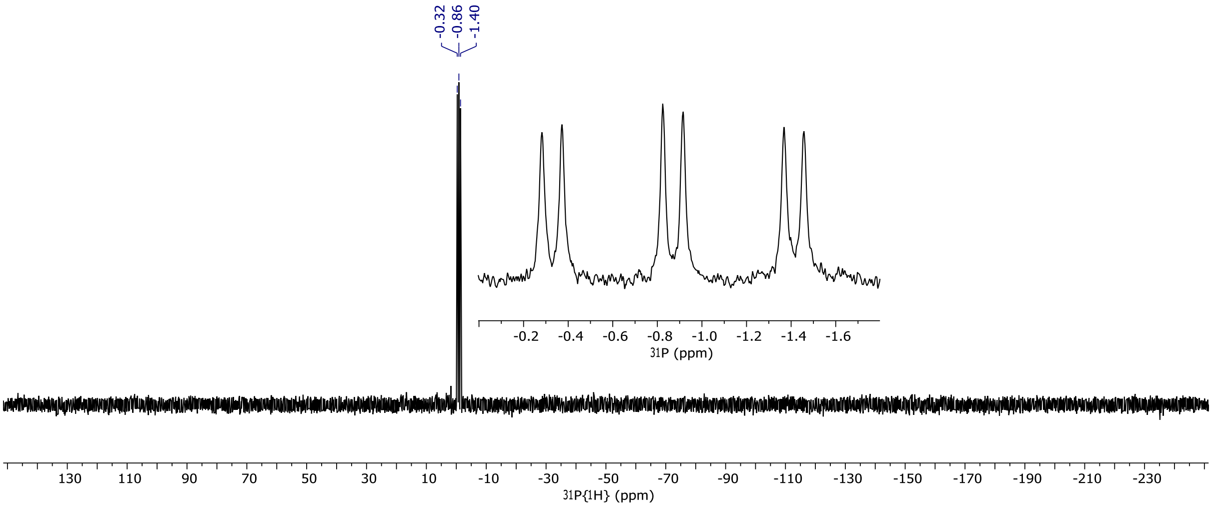

Figure S72: $^{31}P\{^1H\}$ NMR spectrum of $\mathbf{15}^D$ in d_8-THF. Inset: ^{31}P NMR spectrum enhanced between 0.00 – −1.8 ppm.

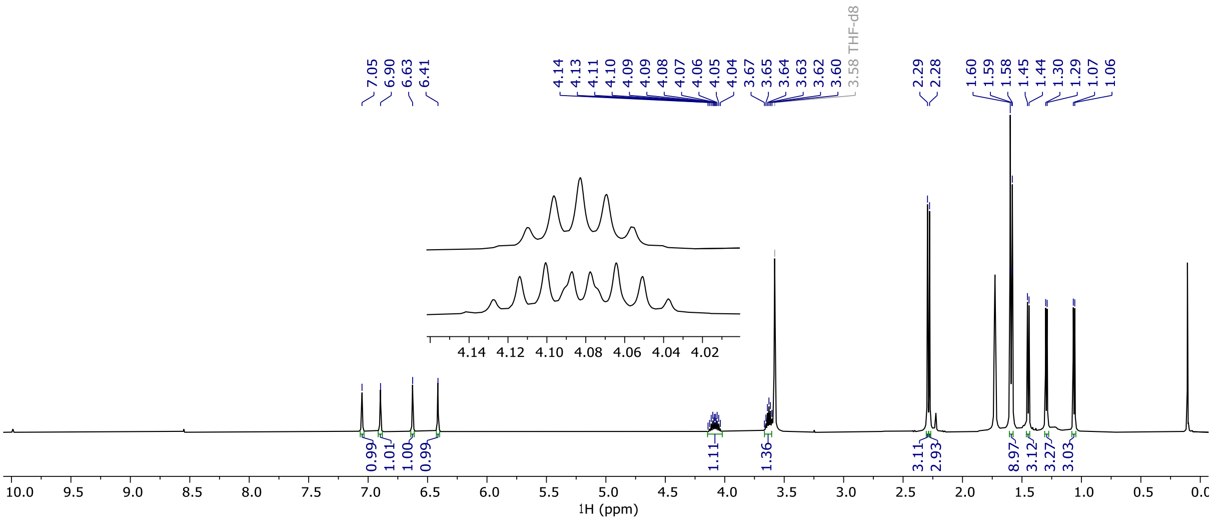

Figure S73: ^1H NMR spectrum of $\mathbf{15}^D$ in d_8-THF. Inset: top is $^1H\{^{31}P\}$ and bottom is ^1H NMR spectrum enhanced between 4.16 – 4.00 ppm. Note the absence signals corresponding to the P–H bond and N–H in **15**.

2.9 Variable temperature NMR study of 2

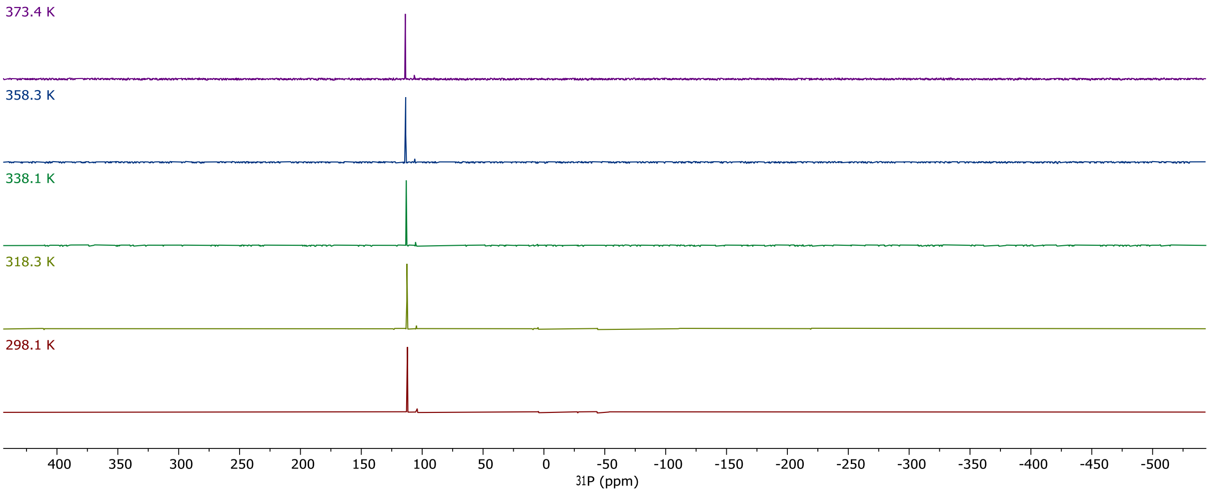

Figure S74: ^{31}P NMR spectrum of dimeric phosphine **2** in *d*$_8$-toluene at increasing temperature. No evidence of dimer dissociation was observed.

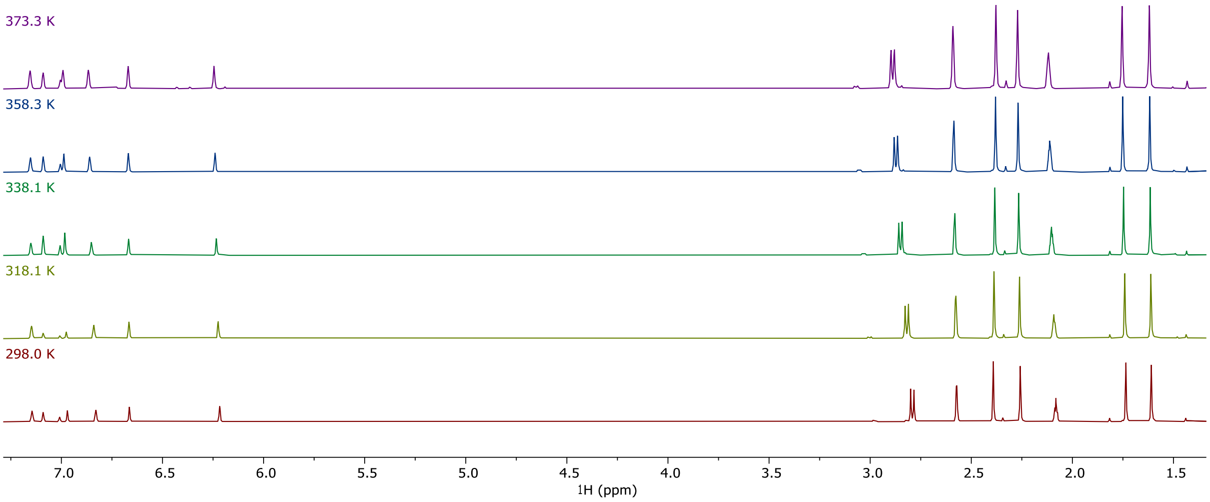

Figure S75: ^{1}H NMR spectrum of dimeric phosphine **2** in *d*$_8$-toluene at increasing temperature. No evidence of dimer dissociation was observed.

2.10 ATIR spectrum of 15

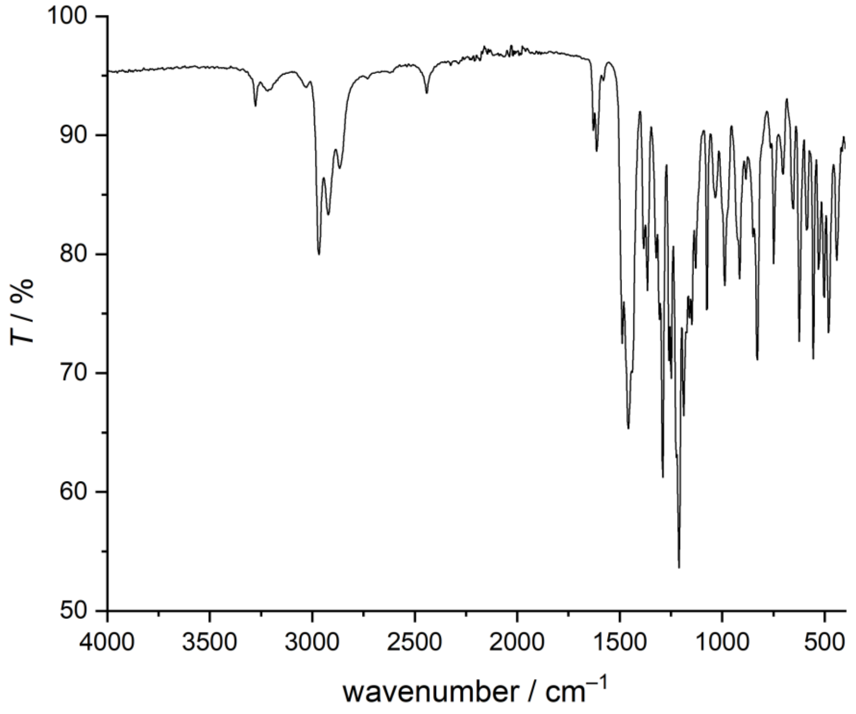

Figure S76: ATIR spectrum of **15**. Characteristic bands corresponding to N–H (3278 cm^{-1}) and P–H (2441 cm^{-1}) stretching vibrations are observed.

3. Single crystal X-ray diffraction data

Single-crystal X-ray diffraction data were collected using an Oxford Diffraction Supernova dual-source diffractometer equipped with a 135 mm Atlas CCD area detector. Crystals were selected under Paratone-N oil, mounted on micro-mount loops and quench-cooled using an Oxford Cryosystems open flow N_2 cooling device. Data were collected at 150 K using mirror monochromated Cu Kα (λ = 1.54184 Å) radiation and processed using the CrysAlisPro package, including unit cell parameter refinement and inter-frame scaling (carried out using SCALE3 ABSPACK).[5] Equivalent reflections were merged and diffraction patterns processed with the CrysAlisPro suite. Structures were subsequently solved using direct methods and refined on F^2 using the SHELXL package.[6]

Table S1. Selected X-ray data collection/refinement parameters for **2, 3$^{\mathbf{Me}}$** and **3$^{\mathbf{\textit{i}Pr}}$**.

	**2**	**3$^{\mathbf{Me}}$**	**3$^{\mathbf{\textit{i}Pr}}$**
Formula	$C_{38}H_{44}N_6P_2$	$C_{19}H_{22}Br_2N_3P$	$C_{23}H_{30}Br_2N_3P$
CCDC	2245882	2245883	2245884
Fw [g mol^{-1}]	646.73	483.18	539.29
Crystal system	monoclinic	monoclinic	monoclinic
Space group	$P2_1/c$	$P2_1/c$	$P2_1/n$
a (Å)	13.6604(1)	10.6582(1)	11.0549(1)
b (Å)	8.6501(1)	20.6927(2)	10.4890(1)
c (Å)	13.9457(1)	17.9575(2)	20.0135(1)
α (°)	90	90	90
β (°)	98.594(1)	103.962(1)	100.466(1)
γ (°)	90	90	90
V (Å^3)	1629.38(3)	3843.46(7)	2282.05(3)
Z	2	8	4
Radiation, λ (Å)	Cu K_α, 1.54184	Cu K_α, 1.54184	Cu K_α, 1.54184
Temp (K)	150(2)	150(2)	150(2)
ρ_{calc} (g cm^{-3})	1.318	1.670	1.570
μ (mm^{-1})	1.503	6.186	5.274
Reflections collected	39989	65795	24074
Indep. reflections	3394	8031	4751
Parameters	214	463	270
R(int)	0.0360	0.0387	0.0566
R1/wR2,[a] I ≥ 2σI (%)	3.38/9.59	3.02/7.99	2.08/5.29
R1/wR2,[a] all data (%)	3.80/9.96	3.22/8.15	2.19/5.38
GOF	1.052	1.046	1.069

[a] $R1 = [\Sigma||F_o| - |F_c||]/\Sigma|F_o|$; $wR2 = \{[\Sigma w[(F_o)^2 - (F_c)^2]^2]/[\Sigma w(F_o^2)^2]\}^{1/2}$; $w = [\sigma^2(F_o)^2 + (AP)^2 + BP]^{-1}$, where $P = [(F_o)^2 + 2(F_c)^2]/3$ and the A and B values are 0.0578 and 0.633 for **2**, 0.0422 and 3.07 for **3$^{\mathbf{Me}}$**, and 0.0291 and 0.95 for **3$^{\mathbf{\textit{i}Pr}}$**.

Table S2. Selected X-ray data collection/refinement parameters for **4^{iPr}, 6** and **7**·hex.

	**4^{iPr}**	**6**	**7**·hex
Formula	$C_{23}H_{30}N_3P$	$C_{28}H_{42}N_3OP$	$C_{26}H_{37}N_3OP$
CCDC	2245885	2245886	2245887
Fw [g mol^{-1}]	379.47	467.61	438.55
Crystal system	orthorhombic	monoclinic	monoclinic
Space group	*Cmc*2$_1$	*C*2/*c*	*C*2/*c*
a (Å)	24.0287(3)	17.5219(2)	30.9090(11)
b (Å)	7.6323(1)	18.0163(2)	9.5766(4)
c (Å)	11.5588(1)	19.4088(2)	34.7072(9)
α (°)	90	90	90
β (°)	90	110.154(1)	106.821(3)
γ (°)	90	90	90
V (Å^3)	2119.82(4)	5751.81(11)	9833.9(6)
Z	4	8	16
Radiation, λ (Å)	Cu K_α, 1.54184	Cu K_α, 1.54184	Cu K_α, 1.54184
Temp (K)	150(2)	150(2)	150(2)
ρ_{calc} (g cm^{-3})	1.189	1.080	1.185
μ (mm^{-1})	1.224	1.008	1.149
Reflections collected	22420	75436	35541
Indep. reflections	2252	6003	10142
Parameters	147	326	621
R(int)	0.0448	0.0374	0.0951
R1/wR2,[a] I ≥ 2σI (%)	2.79/7.29	4.13/12.08	5.88/13.26
R1/wR2,[a] all data (%)	2.95/7.40	4.65/12.62	11.34/15.84
GOF	1.047	1.046	1.020

[a] $R1 = [\Sigma||F_o| - |F_c||]/\Sigma|F_o|$; $wR2 = \{[\Sigma w[(F_o)^2 - (F_c)^2]^2]/[\Sigma w(F_o^2)^2]\}^{1/2}$; $w = [\sigma^2(F_o)^2 + (AP)^2 + BP]^{-1}$, where $P = [(F_o)^2 + 2(F_c)^2]/3$ and the A and B values are 0.0515 and 0.31 for **4^{iPr}**, 0.0729 and 3.46 for **6**, and 0.0576 and 0.00 for **7**·hex.

Table S3. Selected X-ray data collection/refinement parameters for **8, 9** and **10**.

	**8**	**9**	**10**
Formula	$C_{23}H_{30}N_3PS$	$C_{23}H_{30}N_3PSe$	$C_{32}H_{41}N_4P$
CCDC	2245888	2245889	2245890
Fw [g mol^{-1}]	411.53	458.43	512.66
Crystal system	orthorhombic	orthorhombic	monoclinic
Space group	*Pbca*	*Pbca*	$P2_1/c$
a (Å)	19.7422(3)	10.8791(3)	13.3415(1)
b (Å)	10.6844(1)	20.1396(5)	9.0932(1)
c (Å)	20.5719(2)	20.1643(4)	24.9146(2)
α (°)	90	90	90
β (°)	90	90	104.645(1)
γ (°)	90	90	90
V (Å^3)	4339.30(9)	4418.01(19)	2924.36(5)
Z	8	8	4
Radiation, λ (Å)	Cu $K_α$, 1.54184	Cu $K_α$, 1.54184	Cu $K_α$, 1.54184
Temp (K)	150(2)	150(2)	150(2)
$ρ_{calc}$ (g cm^{-3})	1.260	1.378	1.164
μ (mm^{-1})	2.112	3.076	1.023
Reflections collected	4547	47384	72843
Indep. reflections	2252	4630	6107
Parameters	253	261	362
R(int)	0.0483	0.0723	0.0430
R1/wR2,[a] I ≥ 2σI (%)	3.89/11.00	3.62/8.71	4.78/14.29
R1/wR2,[a] all data (%)	4.59/11.63	4.92/9.69	5.22/14.86
GOF	1.049	1.051	1.063

[a] $R1 = [\Sigma||F_o| - |F_c||]/\Sigma|F_o|$; $wR2 = \{[\Sigma w[(F_o)^2 - (F_c)^2]^2]/[\Sigma w(F_o^2)^2]\}^{1/2}$; $w = [\sigma^2(F_o)^2 + (AP)^2 + BP]^{-1}$, where $P = [(F_o)^2 + 2(F_c)^2]/3$ and the A and B values are 0.0629 and 3.49 for **8**, 0.0421 and 3.48 for **9**, and 0.0838 and 1.75 for **10**.

Table S4. Selected X-ray data collection/refinement parameters for **11, 12** and **14**.

	**11**	**12**	**14**
Formula	$C_{25}H_{33}F_3N_3O_3PS$	$C_{24}H_{32}N_3P$	$C_{26}H_{38}ClN_3P$
CCDC	2245891	2245892	2245893
Fw [g mol^{-1}]	543.57	393.49	459.01
Crystal system	tetragonal	monoclinic	monoclinic
Space group	*I*4/*m*	*P*2$_1$/*n*	*I*2/*m*
a (Å)	19.7584(1)	11.5140(3)	9.7169(2)
b (Å)	19.7584(1)	9.5806(3)	14.3405(3)
c (Å)	13.4166(2)	19.8338(7)	18.4709(5)
α (°)	90	90	90
β (°)	90	93.615(3)	100.148(2)
γ (°)	90	90	90
V (Å^3)	5237.77(9)	2183.53(12)	2533.57(10)
Z	8	4	4
Radiation, λ (Å)	Cu K_α, 1.54184	Cu K_α, 1.54184	Cu K_α, 1.54184
Temp (K)	150(2)	150(2)	150(2)
ρ_{calc} (g cm^{-3})	1.379	1.197	1.203
μ (mm^{-1})	2.145	1.205	2.053
Reflections collected	19103	24205	26919
Indep. reflections	2850	4543	2759
Parameters	178	269	175
R(int)	0.0317	0.0800	0.0425
R1/wR2,[a] I ≥ 2σI (%)	3.54/9.76	4.38/9.60	4.25/11.75
R1/wR2,[a] all data (%)	4.19/10.30	7.27/11.00	4.92/12.31
GOF	1.044	1.020	1.051

[a] $R1 = [\Sigma||F_o| - |F_c||]/\Sigma|F_o|$; $wR2 = \{[\Sigma w[(F_o)^2 - (F_c)^2]^2]/[\Sigma w(F_o^2)^2]\}^{1/2}$; $w = [\sigma^2(F_o)^2 + (AP)^2 + BP]^{-1}$, where $P = [(F_o)^2 + 2(F_c)^2]/3$ and the A and B values are 0.0552 and 5.79 for **11**, 0.0439 and 0.79 for **12**, and 0.0674 and 2.00 for **14**.

Table S5. Selected X-ray data collection/refinement parameters for **15** and **16**.

	**15**	**16**
Formula	$C_{23}H_{32}N_3OP$	$C_{24}H_{34}N_3OP$
CCDC	2245894	2245895
Fw [g mol^{-1}]	397.48	411.51
Crystal system	triclinic	triclinic
Space group	*P*–1	*P*–1
a (Å)	9.7475(2)	9.7300(4)
b (Å)	11.4509(2)	11.7503(5)
c (Å)	19.9436(4)	11.7696(5)
α (°)	96.500(2)	119.932(5)
β (°)	101.259(2)	90.877(3)
γ (°)	98.232(2)	101.004(4)
V (Å^3)	2137.75(7)	1134.99(10)
Z	4	2
Radiation, λ (Å)	Cu K_α, 1.54184	Cu K_α, 1.54184
Temp (K)	150(2)	150(2)
ρ_{calc} (g cm^{-3})	1.235	1.204
μ (mm^{-1})	1.271	1.213
Reflections collected	48132	15691
Indep. reflections	8868	4671
Parameters	537	266
R(int)	0.0440	0.0280
R1/wR2,[a] I ≥ 2σI (%)	4.46/11.79	4.98/14.13
R1/wR2,[a] all data (%)	5.47/12.57	5.41/14.52
GOF	1.028	1.076

[a] $R1 = [\Sigma||F_o| - |F_c||]/\Sigma|F_o|$; $wR2 = \{[\Sigma w[(F_o)^2 - (F_c)^2]^2]/[\Sigma w(F_o^2)^2]\}^{1/2}$; $w = [\sigma^2(F_o)^2 + (AP)^2 + BP]^{-1}$, where $P = [(F_o)^2 + 2(F_c)^2]/3$ and the A and B values are 0.0666 and 0.97 for **15** and 0.0779 and 0.70 for **16**.

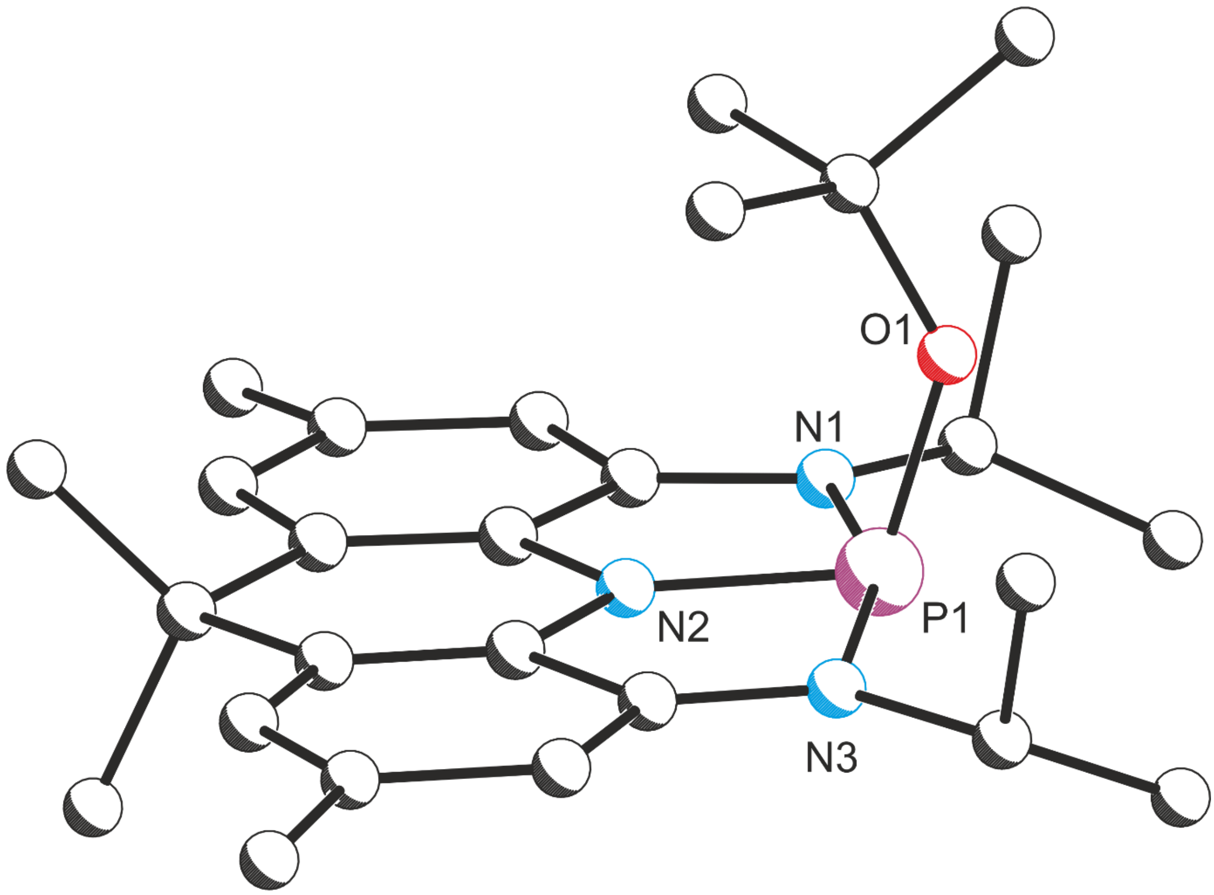

Figure S77: Ball-and-stick diagram of a low-resolution single crystal X-ray structure obtained for **5**. The structure highlights the planarity of the P(NNN) moiety.

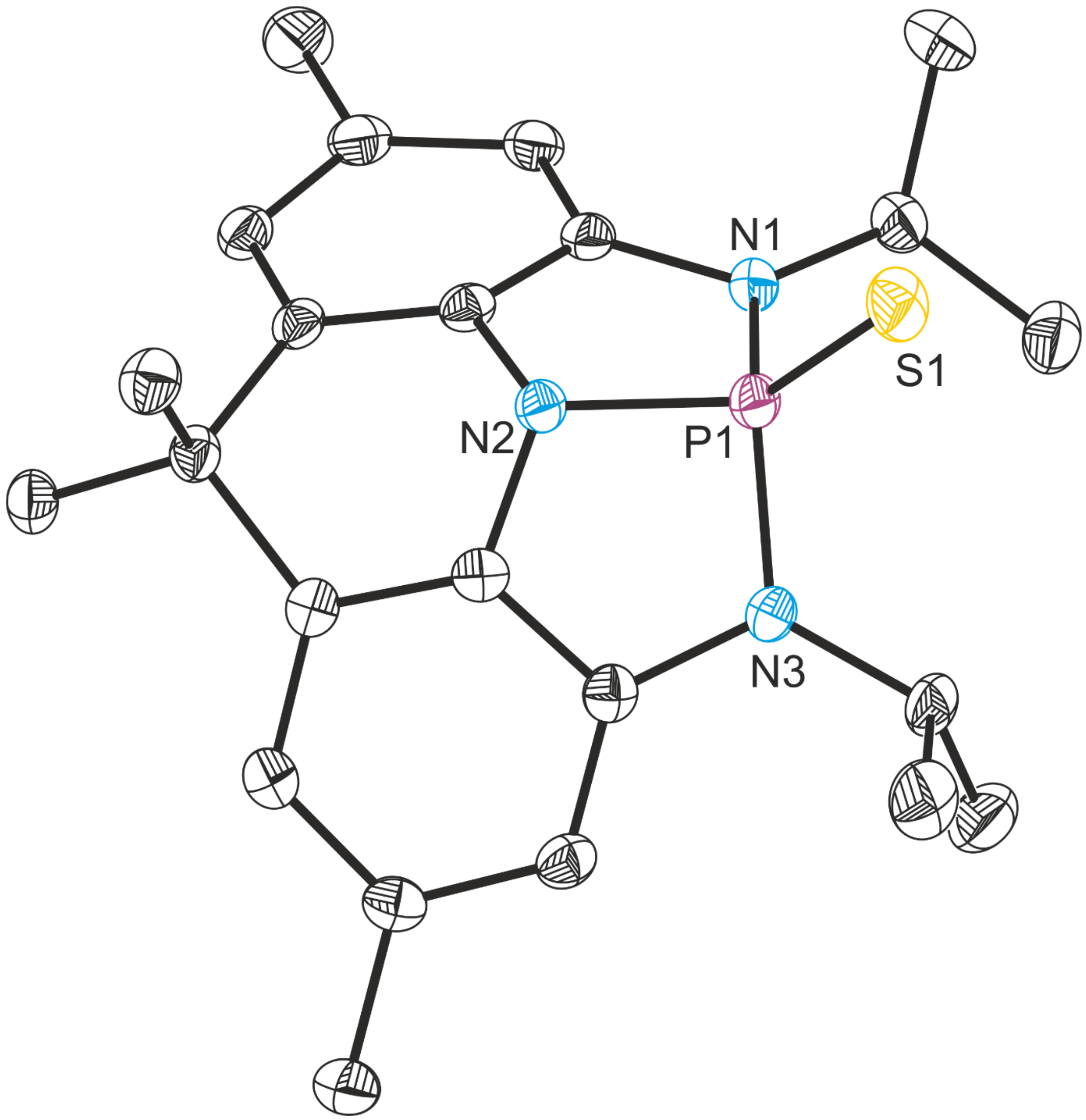

Figure S78: Single crystal X-ray structure of **8**. Thermal ellipsoids pictured at 50% probability level. Hydrogen atoms removed for clarity.

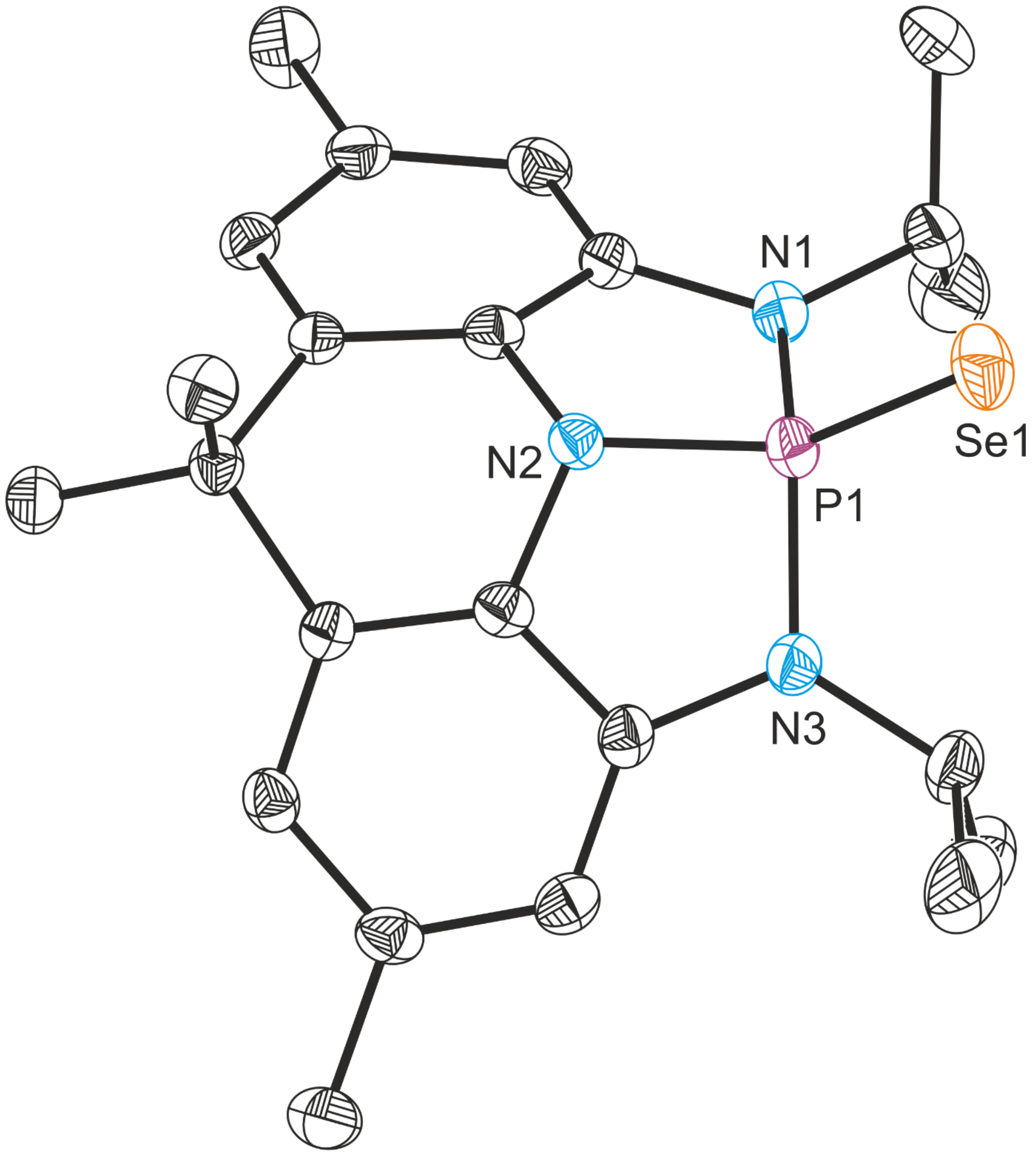

Figure S79: Single crystal X-ray structure of **9**. Thermal ellipsoids pictured at 50% probability level. Hydrogen atoms removed for clarity.

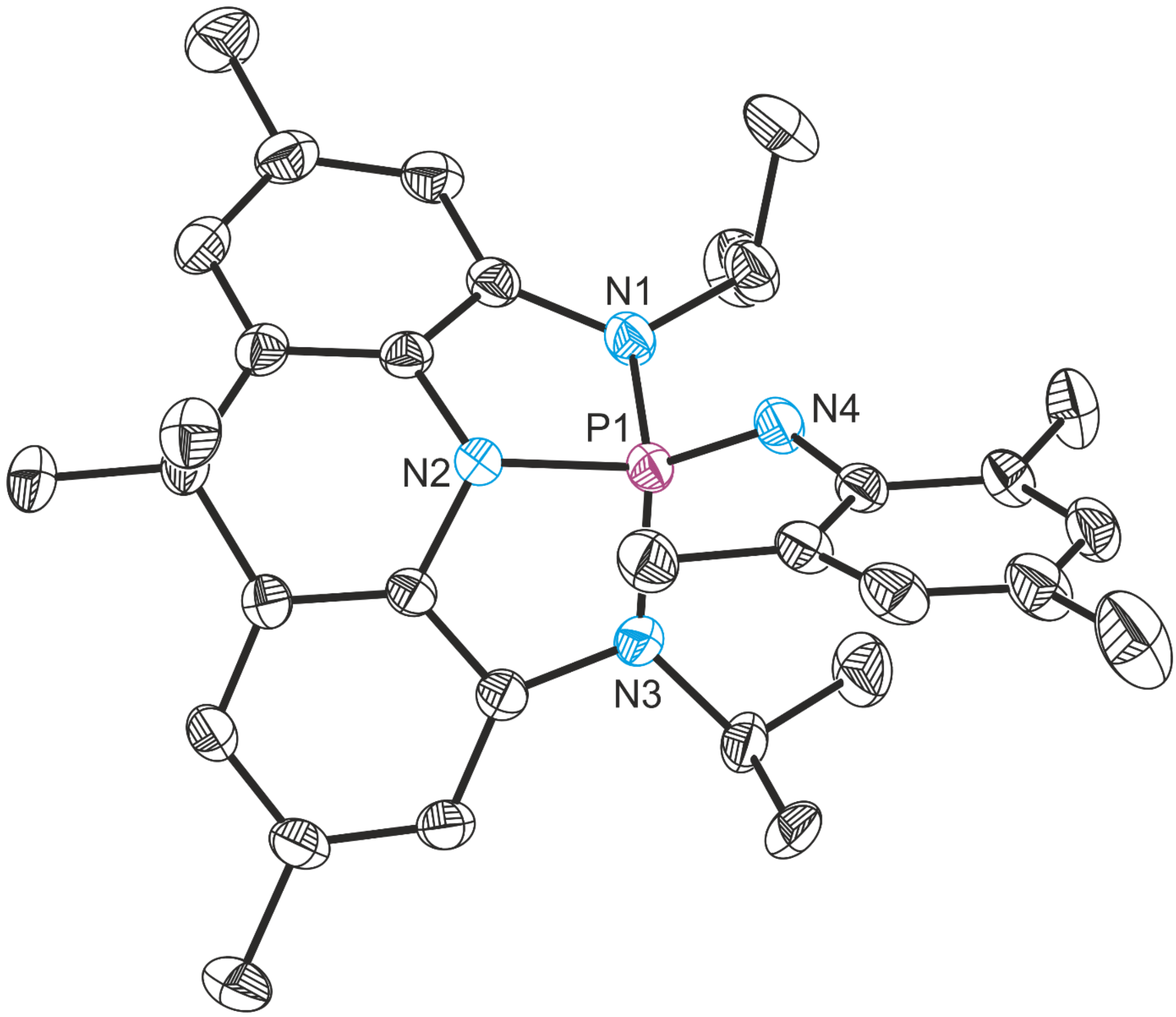

Figure S80: Single crystal X-ray structure of **10**. Thermal ellipsoids pictured at 50% probability level. Hydrogen atoms removed for clarity.

4. References

[1] J. Underhill, E. S. Yang, T. Schmidt-Räntsch, W. K. Myers, J. M. Goicoechea, J. Abbenseth, *Chem. – Eur. J.* **2023**, *29*, e202203266.

[2] A. Hubbard, T. Okazaki, K. K. Laali, *J. Org. Chem.* **2008**, *73*, 316–319.

[3] J.-C. Hierso, *Chem. Rev.* **2014**, *114*, 4838–4867.

[4] T. P. Robinson, D. De Rosa, S. Aldridge, J. M. Goicoechea, *Chem. Eur. J.* **2017**, *23*, 15455–15465.

[5] CrysAlisPro, Agilent Technologies, Version 1.171.41.117a.

[6] (a) G. M. Sheldrick in SHELXL97, *Programs for Crystal Structure Analysis* (Release 97-2), Institut für Anorganische Chemie der Universität, Tammanstrasse 4, D-3400 Göttingen, Germany, 1998; (b) G. M. Sheldrick, *Acta Crystallogr. Sect. A*, **1990**, *46*, 467−473; (c) G. M. Sheldrick, *Acta Crystallogr. Sect. A*, **2008**, *64*, 112−122.
